# Secreted filarial acetylcholine analogs modulate parasite reproduction and host physiology

**DOI:** 10.1038/s41467-026-77090-2

**Published:** 2026-08-29

**Authors:** Alexandra Grote, Lia Ficaro, Joseph Koussa, Rita A. Boateng, Daniela Chow, Johannes Morstein, Morgan E. Brisse, Jennifer Goldstein, Hongyu Wu, Yen-Chih Chen, Matthew Chung, Christopher Mederos, Niels Ringstad, Eric V. Dang, Alexander Aksenov, Heather D. Hickman, Denis Voronin, Dirk Trauner, Drew R. Jones, Elodie Ghedin

**Affiliations:** 1 Center for Genomics & Systems Biology, Department of Biology, New York University, New York, NY USA; 2 Division of Infectious Diseases, Department of Medicine, Feinberg School of Medicine, Northwestern University, Chicago, IL USA; 3 Department of Biochemistry, NYU Langone Health, New York, NY USA; 4 Laboratory of Parasitic Diseases, National Institute of Allergy and Infectious Diseases, National Institutes of Health, Bethesda, MD USA; 5 Department of Chemistry, New York University, New York, NY USA; 6 Laboratory of Viral Diseases, National Institute of Allergy and Infectious Diseases, National Institutes of Health, Bethesda, MD USA; 7 Department of Chemistry, University of Connecticut, Storrs, CT USA; 8 Laboratory of Host Immunity and Microbiome, National Institute of Allergy and Infectious Diseases, National Institutes of Health, Bethesda, MD USA; 9 Department of Cell Biology, NYU Langone Health, New York, NY USA; 10 Department of Chemistry, University of Pennsylvania, Philadelphia, PA USA

**Keywords:** Parasite biology, Biochemical networks

## Abstract

Lymphatic filariasis, caused by *Brugia malayi*, leads to chronic lymphatic dysfunction and lymphedema. The molecular mechanisms underlying interactions between filarial worms and their hosts remain poorly understood. Using untargeted metabolomics, we identify previously unknown acetylcholine analogs synthesized from leucine and isoleucine that are secreted by *Brugia* adult males. In the mammalian host, these metabolites selectively activate host M2 muscarinic acetylcholine receptors, alter lymphatic endothelial cell integrity, and trigger constriction of lymphatic vessels. These metabolites added to cultures also reduce microfilarial production in *B. malayi* and egg-laying in *Caenorhabditis elegans*, implying a role in regulating nematode reproduction. Their production is potently inhibited by ethacrynic acid, an existing FDA-approved drug. Our findings reveal a previously unexplored role for filarial-derived cholinergic signaling in parasite reproduction that impacts host lymphatic homeostasis. This work offers insights into the biochemical adaptations that function in the worm life cycle and reveals potential metabolic vulnerabilities in filarial worms.

## Abstract (web summary)

Filarial worms secrete newly discovered metabolites that affect human lymphatic cells, narrow vessels in mice, and regulate worm reproduction. An FDA-approved drug blocks their production, revealing a potential treatment target.

## Introduction

Parasitic nematodes are the most common parasitic infections of humans worldwide^1^. Filarial worms cause the most severe pathologies, leading to chronic diseases such as lymphatic filariasis, characterized by severe lymphedema, elephantiasis, and hydrocele, which plagues close to 57 million people due to *Brugia malayi, Brugia timori*, or *Wuchereria bancrofti* infections^2^. Filarial parasites develop through three larval stages (L1, L2, and L3) within a mosquito vector before being transmitted to the human host. Transmitted L3 larvae develop into adults in the lymphatic system of the host, where adult female worms release millions of microfilariae per day into the bloodstream^3^. In the lymphatic system, lymphatic endothelial cells (LECs) interact directly with filarial worms and their secreted effectors. Parasites cause morphological and functional changes to the lymphatic endothelial cells in human hosts, and this in turn can damage lymphatic valves, impact lymphatic flow, and lead to a remodeling of the lymphatic vessels because of the proliferation of endothelial cells and connective tissues^4,5^. There is no indicated treatment for lymphedema caused by filarial infection.

Current control and elimination programs for filarial parasites rely on mass administration of a limited set of drugs targeting the microfilarial stage of the parasites^6,7^. No drugs currently exist that permanently sterilize or kill adult worms or are safe to use in areas where other filarial parasites such as *Onchocerca volvulus* (River Blindness) and *Loa loa* (African eye worm) are co-endemic, because rapid killing of microfilariae can cause severe side effects^8–10^. An alternative treatment strategy involves using traditional antibiotics to target the endosymbiotic bacteria from the genus *Wolbachia* that live within most filarial nematodes^11^.

Adult worms can live up to 8 years, and lymphatic filariasis is typically asymptomatic in the chronic phase, characterized by numerous microfilariae in the blood and adult worms in the lymphatic system. The remarkable persistence of these parasites within the human host suggests the evolution of immunomodulatory and metabolic strategies to adapt to the host environment. Significant progress has been achieved in understanding how helminth molecules contribute to evasion and adaptation within the host. Special attention has been given to examining the excretory and secretory (ES) molecules produced at patency, when microfilariae (first-stage larvae) are released from fertile adult filarial female worms^12^. ES products from infective-stage larvae during early infection and initial human contact induce the secretion of IL-10 and differentiation of Treg cells. As a result, several ES molecules have been found to modulate the immune response and promote tissue repair. The best studied ES product is a phosphorylcholine-containing molecule called ES-62 from a rodent filarial worm, *Acanthocheilonema viteae*. ES-62 inhibits the proliferation of CD4+ T cells and B cells, decreases IL-4 and IFNγ production, promotes IL-10 production by B1 B cells, and conditions antigen-presenting cells (APCs) to inhibit Th1 responses^13,14^. ES-62 has also been shown to exhibit bystander anti-inflammatory activity in animal models of arthritis, skin sensitization, and lung airway hyper-reactivity. Effects of ES-62 on the host highlight how filarial worms have evolved a co-adaptation with their hosts, allowing for manipulation of the host immune response. Importantly, the number of ES products is vast, indicating that other molecules with similar effects on immunity and physiology remain to be discovered.

Drug therapies that target adult filarial worms are needed to treat these painful and disfiguring diseases. Parasite-specific metabolic pathways have been attractive targets for drug development programs. A key challenge is to identify which, among the hundreds of enzymes involved in these pathways, can be successfully targeted for therapeutic intervention. One way to explore how the filarial worms adapt to their environment is to characterize the metabolites that are secreted and unique to the worm for survival within the host. With this in mind, we performed a life stage-specific metabolic analysis and identified novel acetylcholine analog metabolites secreted by *Brugia* adult male worms. We found that these molecules were also produced by other filarial species. Acetylcholine is a major excitatory neurotransmitter in nematodes, where it controls many behaviors, including locomotion, egg laying, feeding, and male mating^15^. We show that when chemically synthesized versions of these filarial acetylcholine analogs were used to treat fertile female *B. malayi* worms, there was a significant reduction in microfilariae production. On the host side, the synthesized filarial metabolites specifically activated the M2 muscarinic receptors and could compete with host acetylcholine. When injected into the mouse afferent popliteal lymphatics, the metabolites caused constriction of the lymphatic vessels and increased lymph flow. These findings provide insights into the biochemical adaptations that contribute to parasite survival in their mammalian hosts and reveal an unexplored role for filarial-derived cholinergic signaling in parasite reproduction.

## Results

### Metabolic profiling of filarial worms identifies a unique sex-specific metabolite

**Fig. 1:**
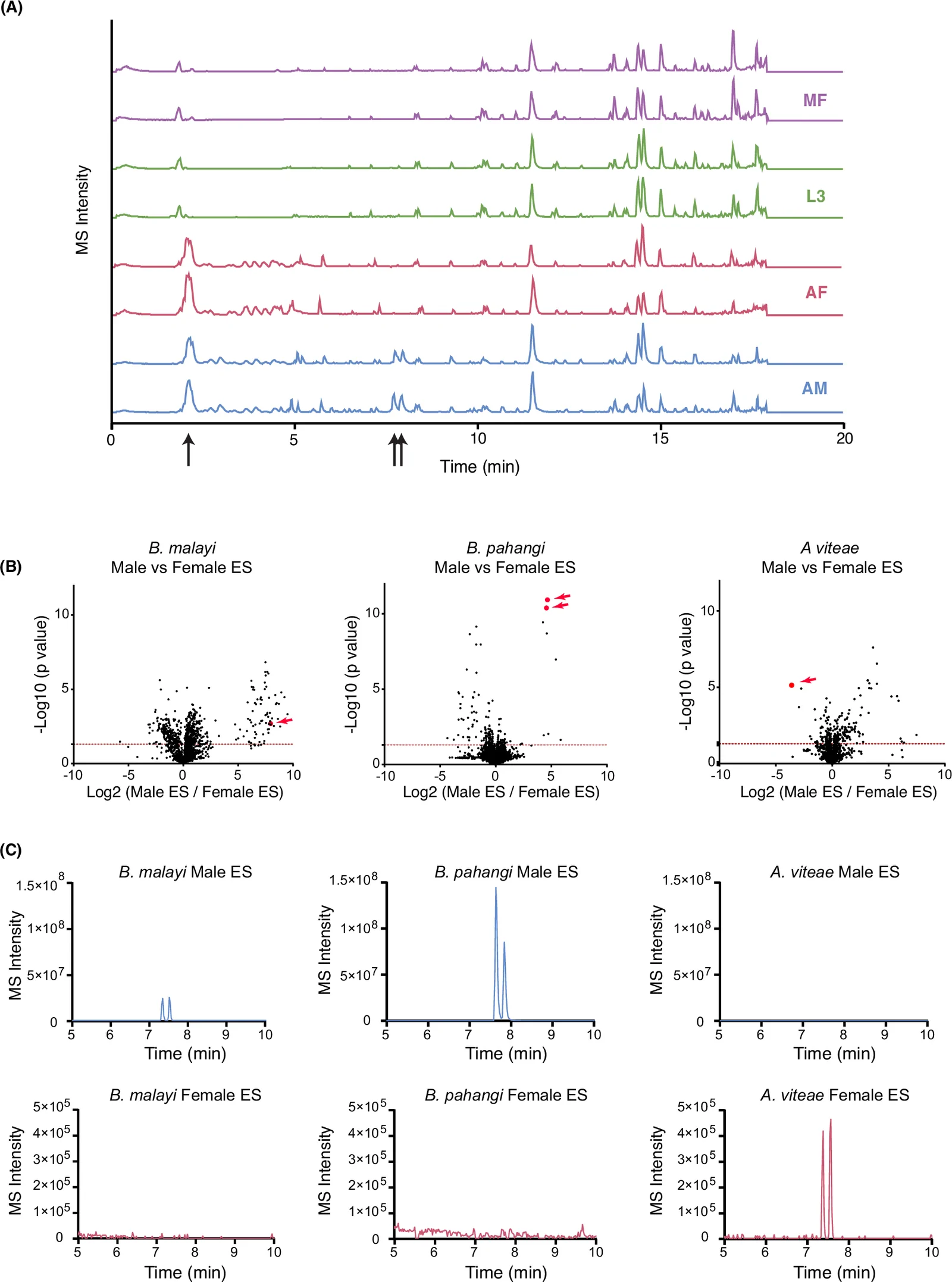
Metabolic profiling of *filarial worms* identifies unique sex-specific metabolites. **A** Global MS profiles (base peak chromatograms) of *B. malayi* developmental stages, two representative replicate samples shown for each, experiments performed in triplicate. Single arrow (at ~2 min) indicates adult-stage specific peak manually annotated as glycerophosphocholine; double arrows (~7.5 min) indicate male-specific peaks corresponding to molecules with no molecular candidates in the available databases. MF microfilariae, L3 larvae stage 3, AF adult females, AM adult males. See also Supplementary Data 1 and 2.** B** Volcano plots of untargeted metabolomics of ES products comparing male and female *B. malayi, B. pahangi*, and *A. viteae* worms (two-tailed *t*-test, −log10 transformed raw *p* values for *y*-axis). Red circles with red arrows indicate metabolite features of interest (188.1645 *m*/*z*) or their natural isotopes. **C** Representative extracted ion chromatograms showing relative quantification of the 2 MS peaks of interest in male and female *B. malayi, B. pahangi*, and *A. viteae* worm ES. See also Supplementary Fig. 1 and Supplementary Data 4–6.

*B. malayi* worms undergo a life cycle that involves drastic metabolic changes that allow the parasites to adapt to their host niche. To better understand parasite metabolism at each stage of development, we compared global metabolic profiles of microfilariae (mf; corresponding to larval stage 1, L1), infective L3 larvae, and adult male and female worms at 120 days (Fig. 1A). Visually distinct, but stage-specific, metabolite profiles (Fig. 1A) were discriminated between life-stages by several metabolite features. For example, a metabolite eluting at 2 min was manually annotated on the basis of MS2 spectra as glycerophosphocholine and was only observed in adult worms. Another pair of isobaric peaks eluting around 7.5 min was visually unique to adult males, but the metabolites could not be identified by MS2 library search. Overall, 313 metabolites were putatively identified by spectral library search against the NIST17 library of standards (Supplementary Data 1) from 2384 metabolite peaks (Supplementary Data 2).

Intrigued by these distinguishing features of male and female worms—and considering the chronic presence of adult worms in the lymphatic system—we set out to assess which metabolites the worms were excreting or secreting into their host environment. We cultured in triplicate adult *B. malayi* worms for 3 days and collected the resulting culture media, which model the excretory/secretory (ES) products. Overall, 266 metabolites were assigned identities using an MS2-based library approach (Supplementary Fig. 1A and Supplementary Data 3). A principal component analysis of this metabolic profile efficiently clustered the sample groups by male spent media, female spent media, and control (unspent media) (Supplementary Fig. 1B). Of the metabolites that most strongly separated the 3 clusters, folic acid was significantly depleted in male ES compared to control media (~3-fold change; two-tailed *t*-test, raw *p* values, *p* = 0.007, Supplementary Data 3, row 59), while female ES levels of folate were not significantly different than control media (*p* = 0.3). This may suggest that *B. malayi* males specifically require folic acid compared to females. Folate is critical in the biosynthesis of purine nucleotides, thymine, and other metabolic reactions, e.g., methylation. Folic acid is also a known product of microbial metabolism and could be linked to the presence of the *Wolbachia* endosymbiont.

A comparison between male and female *B. malayi* ES identified 77 metabolites that differentiated male- and female-conditioned media samples (Supplementary Fig. 1C). Notably, pipecolic acid was significantly up-regulated in female ES compared to male ES (female/male = 1.8-fold change; two-tailed *t*-test, *p* = 0.001) along with lysine (a precursor to pipecolic acid) (Supplementary Data 3, rows 67 and 70, respectively). Further, pyridoxine (B6) was significantly down-regulated in female ES compared to male ES (male/female = 1.5-fold change; two-tailed *t*-test, *p* = 0.0057; Supplementary Data 3, row 243), which may be linked to pipecolic acid function.

An untargeted feature-based analysis was further done to expand the potential scope of discovery (Supplementary Data 4). Overall, 5142 features were quantified across the 3 groups (male ES, female ES, and unspent media). The male ES vs. female ES volcano plot was examined (Fig. 1B), and the same pair of peaks indicative of adult males was detected as significantly (*p* = 0.001) and dramatically higher than in control unspent media (Supplementary Data 4; feature 188.1644, row 1473 for polar LC, row 3451 for hydrophobic LC). These peaks were not detected in the control media, resulting in an estimated fold change of 605 based on the limit of detection.

To determine whether this pair of male-enriched metabolites was *B. malayi*-specific, we repeated the analysis with conditioned media from biological replicates of cultured *B. pahangi* worms, a related species that infects lymph vessels of cats, and *Acanthocheilonema viteae*, a rodent filarial worm. Again, a highly significant pair of metabolite peaks (two-tailed *t*-test, *p* = 4.2e−11) was found in the *B. pahangi* male-conditioned media when compared to the female media (23.5-fold higher in male ES) (Supplementary Data 5, row 2825). For *A. viteae*, the metabolite features were manually added because they were too low in intensity to be automatically picked as an untargeted feature. However, once quantified, the reverse trend was observed for *A. viteae*, with female-conditioned media having higher levels of the two metabolite features compared to the male-conditioned media (12.1-fold higher in females; two-tailed *t*-test, *p* = 7.38e−6) (Supplementary Data 6, row 1712). However, upon inspection, although the pair of peaks was detectable in *A. viteae*, they appear to be minor constituents in this species based on the raw signal values, which were ~300-fold lower than for *B. pahangi* (Fig. 1C). The spectral data from *B. pahangi* and *A. viteae* were compared to the analysis of *B. malayi*, and we confirmed that across these studies, peaks had matching accurate masses, retention times, and MS2 patterns, indicating consistent molecular structures.

### The filarial metabolites are acetylcholine analogs

To determine the structure of the male-specific *Brugia* double-peak from the ES and whole worm studies, we manually annotated the available MS1 and MS2 data to identify a possible match. However, a stringent mass search of common databases such as HMDB and PubChem provided no results at the time of the search, and an MS2 search against the NIST and METLIN libraries produced no candidates. Inspection of the chromatographic data for the feature revealed a pair of closely eluting but separated isobaric peaks, possibly indicating two isomeric structures. The two peaks also had nearly identical MS2 spectra, indicating their overall structural similarity, with only minor fragments distinguishing them. To determine whether we could identify other molecules structurally related to these features of interest, we generated a molecular network. The molecular networking approach^16^ organizes molecular space and connects metabolites with similar MS/MS. Spectral similarity is typically reflective of structural similarity, as the same parts of the molecular structures tend to give rise to the same peaks in MS/MS^16^. An advanced version of molecular networking using the GNPS (Global Natural Products Social Molecular Network) platform allows for capturing connections that may be missed by a conventional networking approach^17^. The network provides an overview of the metabolome produced by the *Brugia* ES, including any novel molecules such as several female-enriched metabolites, which showed structural similarity to DEET (N,N-diethyl-meta-toluamide) that were found predominantly in *Brugia* female ES (Supplementary Fig. 2A, left inset). We observed that the two male-specific isobars of interest only showed similarity to two other network nodes, which were just the natural carbon isotopes of the novel isomers (Supplementary Fig. 2A, right inset).

Therefore, we attempted to solve the structure manually. The MS2 fragment sub-formulas and neutral loss deltas were uniquely assigned by accurate mass using common biological element search ranges (C_0-30_H_0-60_N_0-10_O_0-15_P_0-3_S_0-3_). The fragment formula plus neutral loss formula sum supported a putative intact formula of [C_10_H_22_NO_2_]^+^ for both peaks, supporting the conclusion that they are isomers. Interestingly, despite very high MS1 signal intensities (>10^8^ NL), no other adducts and no correlated negative mode parent ions were found, likely indicating an intrinsic positive charge on the structure. Both MS2 spectra showed a neutral loss of 59.0731 (unambiguously identified as C_3_H_9_N) from the parent ion, which would be consistent with a trimethyl quaternary amine fragment and an intrinsic positive charge on the parent ion. Using this putative substructure information, we proposed fragment skeletons and assembled several candidate scaffolds by hand. We observed that one scaffold had a high degree of similarity to the neurotransmitter acetylcholine, comprising a choline group in an ester linkage to an aliphatic tail. To test whether this was indeed the case, and since acetylcholine did not appear to be present in the *Brugia* ES data, we performed another molecular networking analysis, but this time adding all acetylcholine reference spectra from GNPS. We found that the novel metabolites clustered with acetylcholine and did so via a direct edge, indicating that acetylcholine was the most similar molecule available in the network (Supplementary Fig. 2B).

With this scaffold, three configurations were possible, which we describe with standard nomenclature: a straight chain resulting in pentanoylcholine, and two branched chains resulting in 2-methylbutyryl choline (2-MBC) and isovalerylcholine (IVC). To validate our prediction, we synthesized these compounds (Supplementary Information) and used a spike-in approach to determine if the synthetic compounds were the same as the endogenous peaks (Supplementary Fig. 3). The endogenous peaks are from the male *B. pahangi* ES samples, which we used for this spike-in experiment because this species appeared to secrete the highest amounts of the novel metabolites. These results indicated an identical structure match between the two endogenous metabolites and the two branched-chain synthetic structures (i.e., the “valerylcholines”). This result is supported because the spiked-in synthetic molecules co-eluted with the endogenous peaks but did not change the MS1 or MS2 pattern and added to the signal intensity of the respective peak. Further, a comparison with the synthetic standards alone confirmed that the chromatographic pattern (Supplementary Fig. 4) and the characteristic minor fragments of the MS2 spectra were identical to the endogenous spectra (Supplementary Figs. 4A and 5A, B).

### Acetylcholine analogs are produced by male and female *B. malayi*, but are preferentially secreted by males

**Fig. 2:**
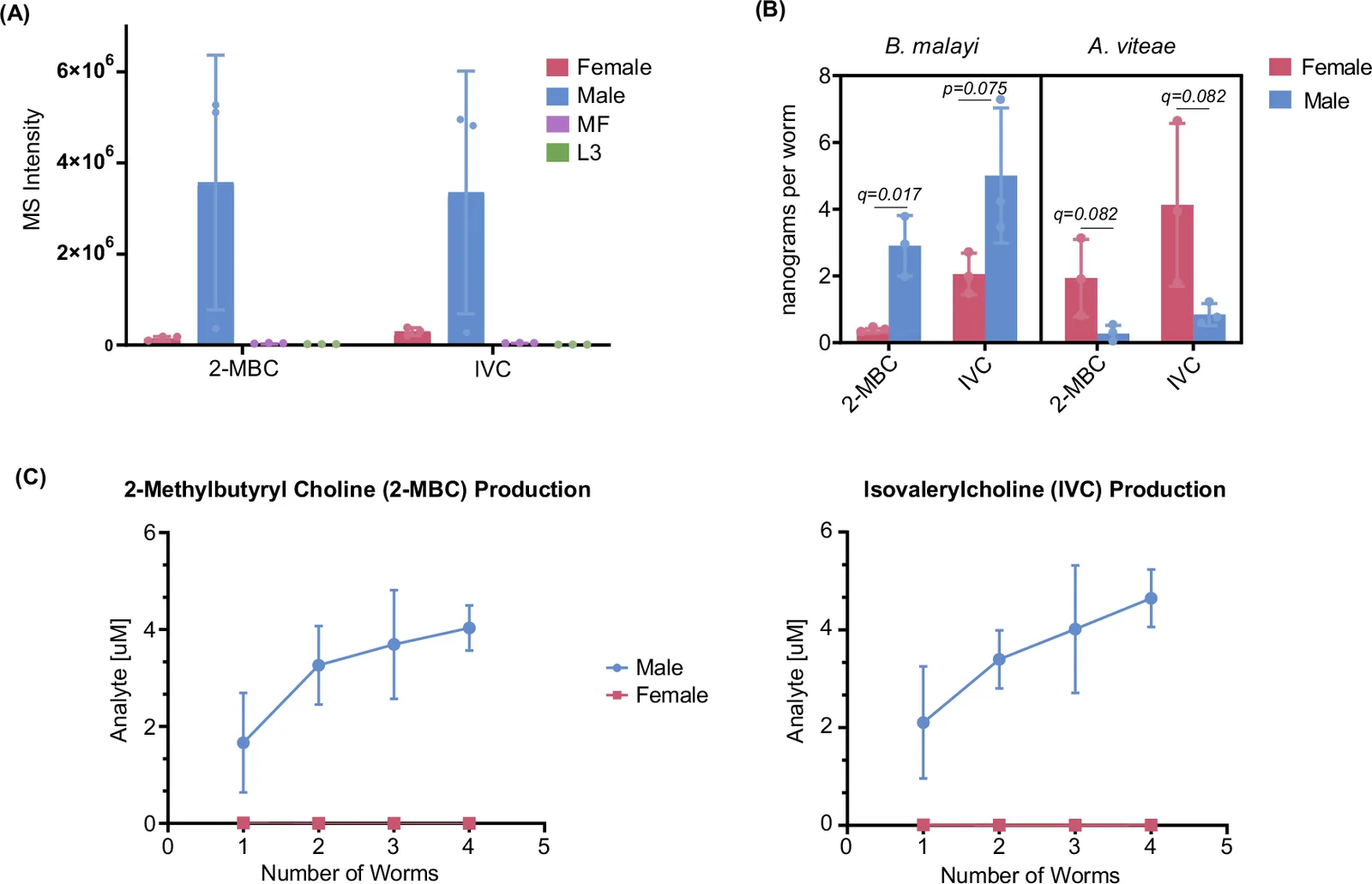
Acetylcholine analogs are produced by male and female *B. malayi* but preferentially secreted by males. **A** Untargeted MS intensity values for *B. malayi* whole worm samples across different life stages. Samples include 20 adult females, 40 adult males, 2 × 10^6^ microfilariae (MF), or 200 L3 per biological replicate, *n* = 3. **B** Targeted quantification of 2-methylbutyryl choline (2-MBC) and isovalerylcholine (IVC) from whole male and female *B. malayi* and *A. viteae* worms (10 worms per biological replicate, *n* = 3), shown as mean nanograms ± standard deviation. Group comparisons were performed using multiple unpaired *t*-tests, with correction for multiple comparisons using the two-stage step-up method of Benjamini, Krieger, and Yekutieli, with the desired false discovery rate set at 1% (*Q* = 0.01); *q*-values are reported in the figure. **C** Targeted quantification from 100 µL each of conditioned media with increasing numbers of *B. malayi* worms, male or female, from 1 to 4 total, in increments of 1, with the spent culture media (ES) collected at 24 h. Experiments were performed 3 times for each. Mean and standard deviation bars are included for each graph. All female data points reached detection, but the data are shown to scale. Source data are provided as a Source Data file.

A standard curve of 2-MBC and IVC was prepared and extracted side-by-side with whole worm samples to quantify the valerylcholines using the same LCMS method (reverse-phase), but with a dedicated parallel reaction monitoring scan (PRM) to target both 2-MBC and IVC at 188.1645 *m*/*z* ± 0.2 *m*/*z* isolation window with quantification of the 129.0910 *m*/*z* fragment ion (Supplementary Fig. 4A). The relative peak intensities of the 2-MBC and IVC transitions were then measured across all samples and standards. The standard curve spanned from a high of 55 μM to a low of 55 nM based on the previous experimental ranges (Supplementary Fig. 4B, C). The standards recapitulated the endogenous response, and the curve samples gave an intense response with good linearity and virtually no background signal or carryover between samples. A reassessment of the original *B. malayi* whole worm samples across different life stages indicated that IVC and 2-MBC were not exclusively produced by males but were detectable (albeit very low) in male, female, and microfilariae (mf) samples, but not L3 (Fig. 2A). We then assessed 2-MBC and IVC in males and females of *B. malayi* and *A. viteae* based on a count of worms (*n* = 10 per sample; biological triplicates). Interestingly, both 2-MBC and IVC were detected above the background threshold in all samples, including males and females from *B. malayi* and *A. viteae*. For *B. malayi*, 2-MBC and IVC were enriched in males by ~2.5 to 8-fold compared to females, while in *A. viteae* the compounds were more abundant in females by 5 to 7-fold compared to males. However, none reached statistical significance (multiple unpaired *t*-tests, FDR at 1%) (Fig. 2B). We then performed a targeted analysis of conditioned media with increasing numbers of *B. malayi* worms from 1 to 4 total (Fig. 2C). There was a clear increase in metabolite production with increased numbers of male *B. malayi* worms. Therefore, while the valerylcholines are present in most whole-worm materials, it is the *Brugia* males that secrete large amounts into their environment.

### Biosynthetic pathways of filarial acetylcholine analogs use leucine and isoleucine as precursors

**Fig. 3:**
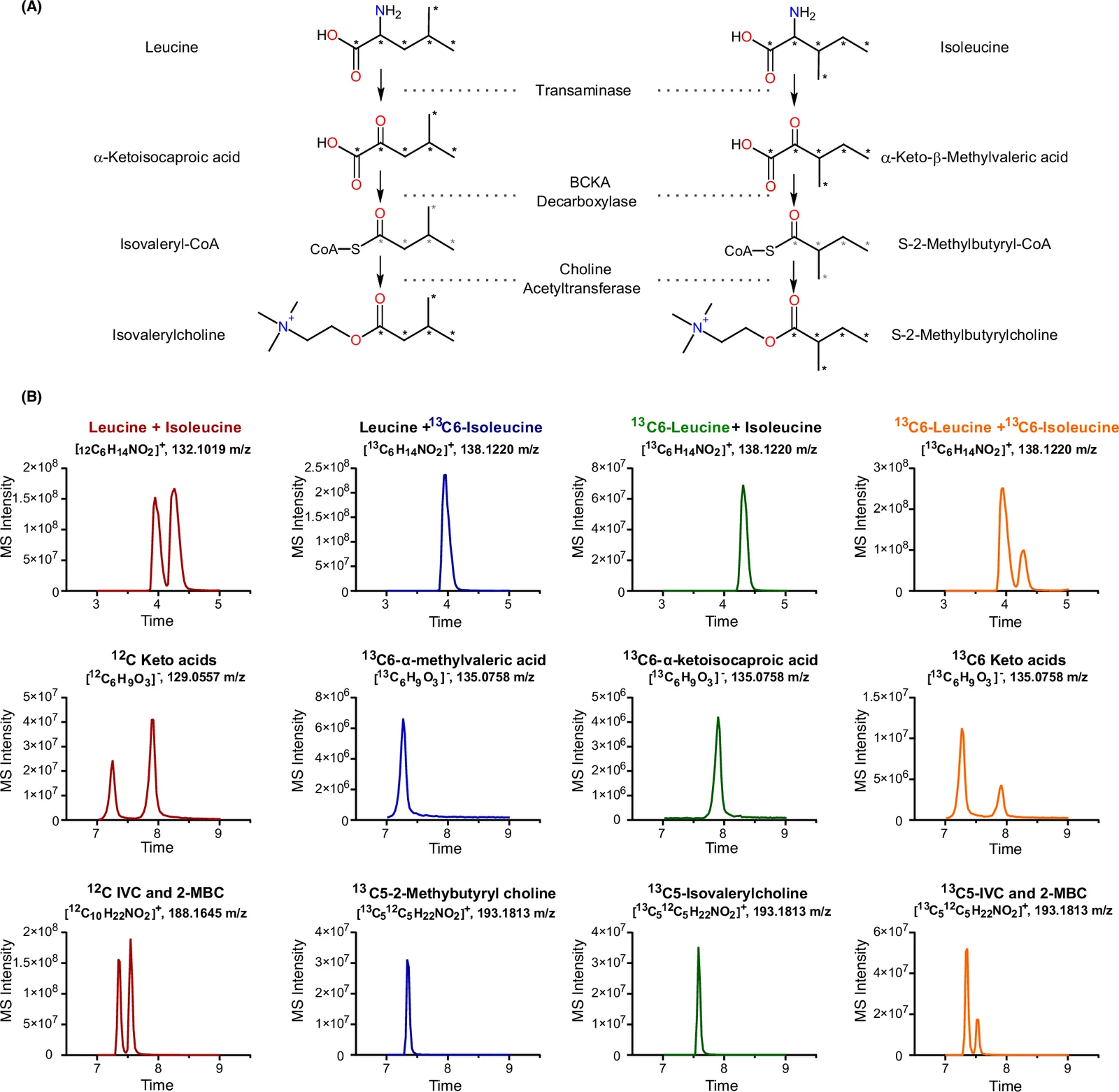
Biosynthetic pathways of filarial acetylcholine analogs use leucine and isoleucine as substrates. **A** Hypothesized pathway for creation of isovalerylcholine (IVC) from leucine and 2-methylbutyryl choline (2-MBC) from isoleucine. The final product retains 5 carbons from the initial leucine or isoleucine pathway. * Indicates detected carbon-13 isotope data, * (gray) indicates inferred but not detected. **B** Extracted ion chromatograms (MS1) for isovalerylcholine and 2-methylbutyryl choline pathways from spent media of cultured male worms. Representative MS peaks were hand-annotated and transferred to GraphPad Prism. The top row shows MS intensity as a function of retention time for leucine and isoleucine accurate mass, the middle row shows keto acid intermediates for the hypothesized pathways, and the lower row shows the valerylcholine ester products. See also Supplementary Fig. 5. Source data are provided as a Source Data file.

Having confirmed that the two filarial valerylcholines are analogs of acetylcholine, we hypothesized that the filarial biosynthesis pathway of 2-MBC and IVC may also be partially conserved with acetylcholine biosynthesis. Across bilaterians, the final step of acetylcholine synthesis is carried out by the enzyme choline acetyltransferase (ChAT), which uses coenzyme A (CoA) charged with an acetyl group to esterify with choline. Working under the model that the filarial ChAT was more tolerant of longer chain aliphatic groups, we sought to explain the CoA-linked precursor of the branched aliphatic tails on 2-MBC and IVC. With a literature review^18^, we realized that the branched-chain fatty acid synthesis pathway produces a CoA-charged acyl intermediate through the enzyme branched-chain keto acid decarboxylase (BCKA). Further, the branched tail of the valerylcholines was highly reminiscent of the R group of the branched-chain amino acids leucine and isoleucine. Canonically, leucine and isoleucine can be metabolized by a transaminase to generate their respective keto-acids, which are then charged onto CoA by the BCKA. We hypothesized that leucine and isoleucine may be the precursors of IVC and 2-MBC through the action of this proposed pathway (Fig. 3A). Therefore, we carried out an isotope fate study in *B. malayi* adult male worms in culture to trace uniformly carbon-13-labeled leucine and isoleucine into IVC and 2-MBC. We used leucine/isoleucine dropout media and systematically replaced these components to determine their metabolic fate (Fig. 3B). We predicted a +5 mass shift in IVC and 2-MBC deriving from leucine and isoleucine (6-carbon structures), respectively, because the proposed reactions involve loss of one labeled leucine/isoleucine carbon in the second step of the pathway by decarboxylation to generate the CoA intermediate. By targeted MS, we indeed found a shift of +5 (but not +6) for both IVC and 2-MBC, and also observed labeled versions for each of the proposed keto-acid intermediates. Labeled leucine exclusively shifted the mass of IVC, confirming leucine as the precursor of IVC, while labeled isoleucine exclusively shifted the mass of 2-MBC, confirming isoleucine as the precursor of 2-MBC. We also observed carbon-13 specific mass shifts (delta = 1.00335) at the MS2 level, which were consistent with our original substructure assignments when we proposed the structures (Supplementary Fig. 5C–F). Further, because the pathway reactions do not alter the aliphatic tail, 2-MBC must retain the chirality of the isoleucine precursor, thereby confirming the 2-MBC stereocenter to be in the S-configuration (S-2-MBC). Notably, no putative peak was detected for the CoA intermediate, either labeled or unlabeled. However, non-detection is common for many CoA metabolites as they can be covalently bound to the enzyme without extensive sample processing and are generally considered unstable and may degrade during sample preparation.

### Doxycycline treatment increases, and ethacrynic acid treatment decreases, filarial acetylcholine analog production by male *B. malayi* worms

**Fig. 4:**
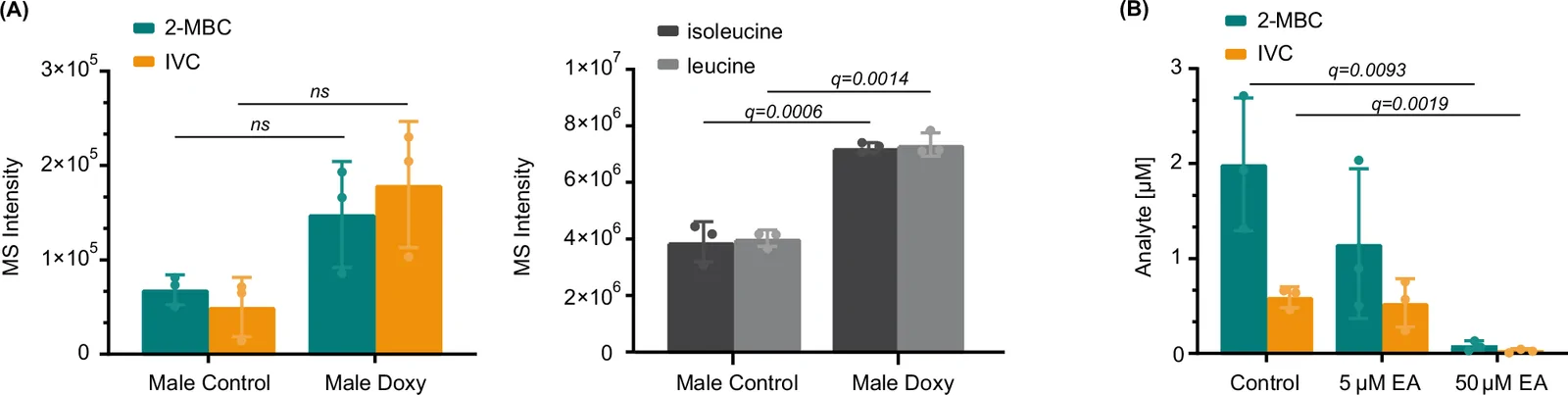
Doxycycline treatment increases, and ethacrynic acid treatment decreases, filarial acetylcholine analog production by male *B. malayi* worms. **A** Effect of doxycycline treatment on production of 2-methylbutyryl choline (2-MBC) and isovalerylcholine (IVC) (left panel), and isoleucine and leucine (right panel) by male *B. malayi* adult worms (whole) treated with 12.5 µM of doxycycline, shown as mean MS intensity ± standard deviation. Group comparisons were performed using multiple unpaired *t*-tests, with correction for multiple comparisons using the two-stage step-up method of Benjamini, Krieger, and Yekutieli, with the desired false discovery rate set at 1% (*Q* = 0.01); significant *q*-values are reported in the figure. Fold increase of 2-MBC and IVC in whole worms in response to doxycycline treatment is not significant (ns, *q* = 0.077). Experiments were performed with ten males for each biological replicate (*n* = 3). **B** Effect of ethacrynic acid (EA) treatment on quantities of 2-MBC and IVC in the culture media (ES) of male *B. malayi* adult worms, shown as mean µM of the analyte ± standard deviation. Experiments were performed with 3 males for each biological replicate (*n* = 3). Multiple unpaired *t*-tests, as in (**A**), with significant *q*-values reported in the figure for comparisons with the control. Source data are provided as a Source Data file.

Filarial nematodes such as *B. malayi* have evolved a mutualistic association with the bacterial endosymbiont *Wolbachia*. These bacteria are essential for worm development, reproduction, and survival^19^ and can be eliminated from the worms by treating infected hosts with doxycycline^20^. To evaluate if doxycycline treatment of cultured *B. malayi* would impact the synthesis of IVC and 2-MBC in adult male worms, groups of 10 adult male worms were cultured with a concentration of 12.5 µM doxycycline for 3 days. This dose and time course were previously shown in other studies to impact *Wolbachia* while avoiding killing adult *Brugia* worms^21,22^. The experiment was performed in triplicate, and an untargeted feature-based analysis was carried out on whole worms. Compared to controls, the doxycycline-treated males showed changes in the ratio of the two isomers with an overall up-regulation of both valerylcholines in the doxycycline-treated samples (~2.2-fold for 2-MBC, 3.6-fold for IVC; multiple unpaired *t*-tests, FDR at 1%, *q* = 0.077) (Fig. 4A, left panel). We also observed that doxycycline treatment led to a significant increase of 1.8-fold in both leucine (*q* = 0.0006) and isoleucine (*q* = 0.0014), the precursors of the valerylcholines (Fig. 4A, right panel). These results suggest *Wolbachia*, which is impacted by the antibiotic treatment, may be competing with the worms for the amino acids or nutrients, including leucine and isoleucine, leading to increased availability for other downstream metabolite products such as the valerylcholines. An alternative interpretation is that doxycycline is inhibiting acetylcholinesterase activity, as seen in other studies^23^, which would lead to an increase in acetylcholine analogs in the treated worms because of less degradation.

We identified Bma-unc-17 (WBGene00227648) as the *B. malayi* choline acetyltransferase (ChAT), an ortholog of *C. elegans* cha-1. Analysis of our previously published life-stage-specific RNA-seq data^24^ revealed that Bma-unc-17 expression is significantly upregulated in both juvenile (30 DPI) and adult (120 DPI) males compared to females (FC = 5.6 and 1.8, respectively, FDR < 0.02 for both comparisons), providing a molecular basis for the observed sex-specific differences in ChAT activity and acetylcholine analog secretion. Additionally, a prior study reported unusually high ChAT in *Brugia*, and further noted males had ~5-fold higher ChAT activity than females^25^. The authors proposed that ChAT may be critical for regulating worm motility, and therefore sought to inhibit the filarial ChAT, ultimately reporting the finding of ethacrynic acid (EA) as a potent and irreversible inhibitor of the enzyme, resulting in worm paralysis. We tested the impact of EA on the biosynthesis of 2-MBC and IVC to measure the activity of the filarial ChAT. When EA was added at 50 μM in the male worm culture media, we observed a profound decrease in the levels of both 2-MBC (18-fold decrease; unpaired *t*-test, *q* = 0.0093) and IVC (23-fold decrease; unpaired *t*-test, *q* = 0.0019) when compared with the vehicle control (Fig. 4B).

### Filarial acetylcholine analogs impact genes involved in worm reproduction

**Fig. 5:**
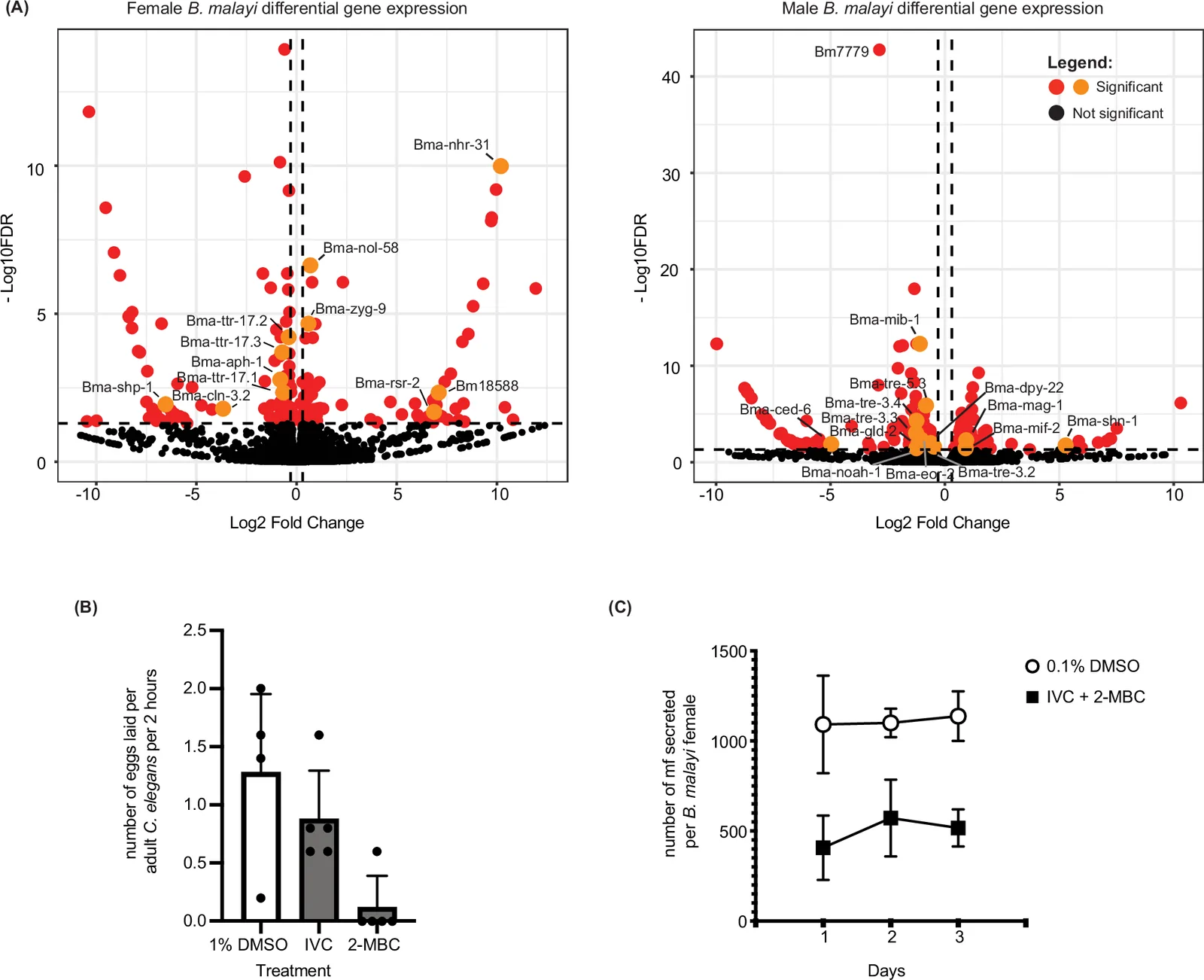
Filarial acetylcholine analogs impact genes involved in worm reproduction. **A** Volcano plot of gene expression in treated female and male worms, genes are plotted by log_2_(FoldChange) by −log_10_(FDR). Enrichment was assessed using a one-tailed hypergeometric test, with *p* values adjusted for multiple testing using the Benjamini–Hochberg procedure. Terms with an adjusted *p* value < 0.05 were considered significant. Significantly differentially expressed genes are plotted in red and orange; genes that are not significantly differentially expressed are plotted in black. Orange denotes genes that have an annotation. Dashed lines indicate a −log2FC < −0.3 or >0.3. Source data are provided in Supplementary Data 7 and 8. **B** Effect of each metabolite at 10 µM on *C. elegans* egg laying, compared to control (1% DMSO) shown as mean number of eggs laid per *C. elegans* adult worm per 2 h, ±standard error for *n* = 5 per group. One-way ANOVA with adjusted *p* value = 0.0306 for isovalerylcholine (IVC) and 0.0002 for 2-methylbutyryl choline (2-MBC). **C** Effect of IVC and 2-MBC combined at 10 µM compared to control (0.1% DMSO) on *B. malayi* microfilaria (mf) production by females on day 1 (control *n* = 6; treated *n* = 7), day 2 (control *n* = 5, treated *n* = 7), and day 3 (control *n* = 4, treated *n* = 6). Data are presented as mean number of mf ± standard deviation. Unpaired *t*-test: day 1, *p* = 0.0002; day 2: *p* = 0.0004; day 3: *p* < 0.0001. Source data are provided as a Source Data file.

To determine the effects of valerylcholine metabolites on filarial parasites, we profiled the transcriptomes of untreated and metabolite-treated male and female *B. malayi* worms. We treated 5 male and 5 female worms with 10 µM of synthetic 2-MBC and IVC for 5 days, replacing 50% of the spent media at day 2. The experiment was performed in duplicate, and mRNA was extracted from whole worms for RNA sequencing (RNA-seq). For female worms, we identified 217 differentially expressed genes (83 upregulated, 134 downregulated) using an FDR of <0.05 (Fig. 5A and Supplementary Data 7). GO term enrichment analysis of all differentially expressed genes revealed an enrichment of GO:0009986, cell surface, *p* value = 2.716E−2 using a hypergeometric test. Among the differentially expressed genes with this GO term were three Transthyretin-like (ttl) family genes, one of the largest conserved nematode-specific gene families, which are thought to have diverse functions and have been shown to be the dominant inducer of the filaria-specific IgE antibody response^26^. Among the other most significantly upregulated genes in female worms upon treatment with the valerylcholines was *Bma-nhr-31*, the homolog of which is known to be involved in embryo development in *C. elegans*^27^, as well as several other genes with homologs involved in embryo development and sex determination in *C. elegans*, including *nol-58*, *zyg-9*, and *rsr-2*. Among the downregulated genes were two of the three ttl-family genes as well as *Bma-shp-1*, a gene generally found to be upregulated in adult female *B. malayi* worms and microfilariae and thought to play a major role in host immune modulation^28^. Also downregulated was gene *Bma-aph-1*, the homolog of which is essential for Notch signaling events throughout development in *C. elegans*^29^, and *Bma-cln-3.2*, which encodes the protein Battenin that is involved in the regulation of oviposition in *C. elegans*^30^.

For treated male worms, we found 318 differentially expressed genes (142 upregulated, 239 downregulated) using an FDR of <0.05 (Fig. 5A and Supplementary Data 8). Among the downregulated genes, we found the GO term GO:0015927 Trehalose activity to be significantly enriched (FDR = 2.412E−2) using a hypergeometric test and FDR correction. In nematodes, trehalose is known to act as a stress protectant and energy source and is known to assist in egg hatching. When male and female worms have diminished or impaired synthesis of trehalose, they show reduced motility and viability^28^. Among the other downregulated genes were *Bma-mib-1* and *Bma-trr-*1, the homologs of which are both required for spermatogenesis in *C. elegans*^29,30^. Also downregulated were *Bma-eor-2* and *Bma-dpy-22*, the homologs of which are required for ras-dependent vulval fate specification in *C. elegans*^31,32^, *Bma-gld-2* and *Bma-noah-1*, required for gametogenesis and embryogenesis, respectively, in *C. elegans*^33,34^, and *Bma-ced-6*, the homolog of which is required for the engulfment of apoptotic cells in *C. elegans*^35^. Among the upregulated genes in treated males was *Bma-mag-1*, which is associated with male-promoting genes resulting in feminization of the hermaphroditic germline in *C. elegans*^36^, *Bma-shn-1*, which regulates defecation, pharyngeal pumping, and putatively male fertility^37^, and *Bma-mif-2*, involved in cellular maintenance during periods of adverse conditions that lead to developmental arrest in *C. elegans*^38^.

Based on the *B. malayi* RNA-seq results, we hypothesized that the valerylcholines would have effects on reproduction. We therefore tested the metabolites on *C. elegans* and *B. malayi* and quantified egg laying (Fig. 5B) and microfilarial production (Fig. 5C), respectively. We found that both IVC and 2-MBC led to a significant decrease in egg laying in *C. elegans* compared to the DMSO control (One-way ANOVA, adjusted *p* value < 0.05 for both metabolites) and a significant decrease in *B. malayi* microfilarial production over all 3 days tested (unpaired *t*-test, *p* < 0.0005).

### Filarial acetylcholine analogs activate mammalian muscarinic acetylcholine receptor M2

**Fig. 6:**
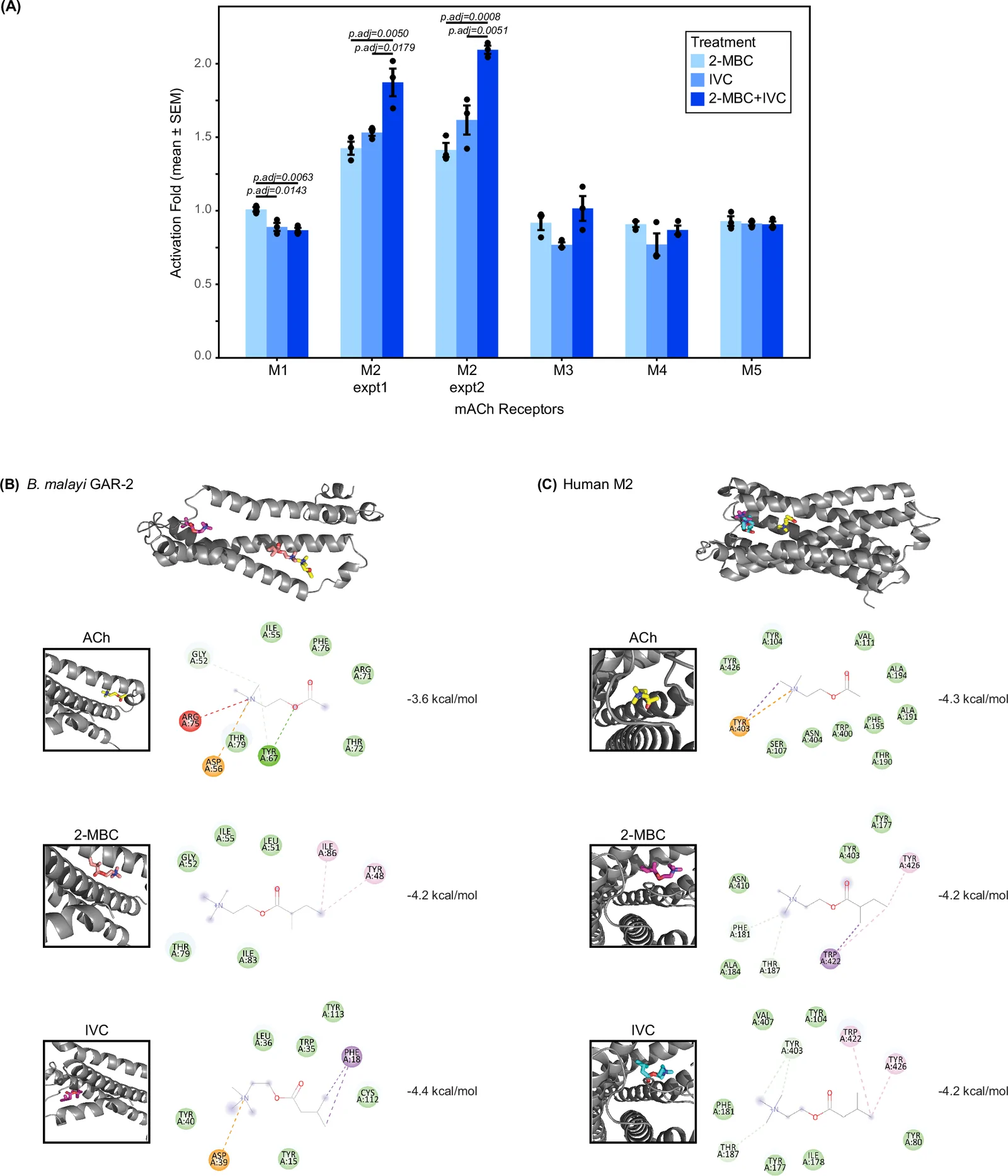
Filarial acetylcholine analogs activate muscarinic acetylcholine receptor M2. **A** Fold-activation of each mAChR in the PRESTO-Tango experiment using isovalerylcholine (IVC) and 2-methylbutyryl choline (2-MBC) at 20 µM each and in combination (*n* = 3 for each treatment). Data are presented as mean fold-change ± standard deviation. Differences in fold change between muscarinic receptors and between treatments were analyzed using one-way ANOVA, followed by Tukey’s HSD multiple comparisons test, with adjusted *p* values (*p*.adj) used for significance thresholds. Receptor differences were observed under all treatments, where M2, tested twice to validate the results (expt1 and expt2), showed significantly higher activation when compared to M1, M3, M4, and M5 (*p*.adj <0.0001), whereas no differences were observed among the latter receptors. Within muscarinic receptors, differences between treatments were found to be significant for M1 and M2. Adjusted *p* values (*p*.adj) are reported in the figure. Source data are provided as a Source Data file. **B** Affinity and selectivity of the filarial valerylcholines to the *B. malayi* GAR-2 receptor. **C** Affinity and selectivity of the filarial valerylcholines to the Human M2 receptor. See also Supplementary Data 9.

Acetylcholine is the primary excitatory neurotransmitter in *C. elegans*, which governs critical reproductive behaviors such as egg-laying and male mating^39^. The neurotransmitter acetylcholine activates muscarinic acetylcholine receptors (mAChRs), which belong to the G protein-coupled receptor (GPCR) superfamily. We tested whether IVC and 2-MBC could activate any of the five mAChRs encoded by the human genome (M1 to M5, encoded by genes CHRM1-5). For this, we used the PRESTO-Tango platform, a luciferase reporter-based system that converts activation into a luminescence signal that can be measured for high-throughput and quantitative screening for GPCR-ligand interactions^40^. We found that IVC and 2-MBC each specifically activate the M2 receptor, and when combined, they have an additive effect that leads to a 2-fold activation of the receptor (Fig. 6A).

Based on the observed activation of the M2 acetylcholine receptor from the PRESTO-Tango experiment, we tested the affinity and selectivity of the valerylcholines for the human muscarinic acetylcholine M2 receptors by generating predictions of the structural basis of receptor-ligand interactions. We also tested the affinity and selectivity to the *B. malayi* receptor. *B. malayi* has a set of two G protein-linked acetylcholine receptors (GARs), Bma-GAR-2 and Bma-GAR-3^41^, and Bma-GAR-2 is thought to be the most likely M2/M4-like inhibitory muscarinic receptor counterpart. A total of 10 docked poses were generated per receptor-ligand complex, and the first binding poses, which refer to the ones with the lowest (best) docking energy and the top-scoring conformation, were evaluated. The more negative the energy score (kcal/mol), the greater the binding (Supplementary Data 9). For the *B. malayi* GAR-2, acetylcholine (parent molecule) and 2-MBC were predicted to bind in a pocket on the extracellular surface proximal to the N-terminus of transmembrane 4 (TM4), while IVC was predicted to bind an intracellular site between TM3 and TM5 (Fig. 6B). These models predicted that binding of 2-MBC and IVC metabolites to the *B. malayi* GAR-2 (−4.2 and −4.4 kcal/mol for 2-MBC and IVC, respectively) was more energetically favorable than binding of acetylcholine (−3.6 kcal/mol). This better binding of IVC and 2-MBC to the *B. malayi* receptor was characterized by several important molecular interactions. Pi (π) molecular interactions were observed with binding cavity residues Tyr48, Ile86 (π-alkyl), and Phe18 (π-sigma). Additionally, van der Waals interactions were formed with receptor residues Tyr15, Trp35, Leu36, Tyr40, Leu51, Ile55, Gly52, Thr79, Ile83, Tyr113, and Cys112, as well as one attractive charged interaction with Asp39.

A comparison model of ligand interactions predicted more favorable binding of acetylcholine to the human M2 receptor (−4.3 kcal/mol) than to the *B. malayi* GAR-2 (−3.6 kcal/mol) (Fig. 6C). 2-MBC was predicted to bind both human and *B. malayi* receptors with similar energy (−4.2 kcal/mol), whereas IVC was predicted to have less favorable binding to the human receptor (−4.2 kcal/mol) compared to the *B. malayi* receptor (−4.4 kcal/mol). Interestingly, the valerylcholines had higher affinity than acetylcholine for the *B. malayi* receptor, but equal affinity for the human receptor as acetylcholine, potentially indicating that the valerylcholines may be a way for males to modulate female signaling. Molecular interactions established between human receptor-ligand complexes include the formation of van der Waal bonds with aromatic residues (Tyr80, Tyr104, Tyr177, Phe181, Thr187, Tyr403), hydrophobic residues (Ile178, Ala184, and Val407), and Asn410 that line the binding cavity. Additionally, three carbon-hydrogen bonds were formed with amino acid residues Phe181, Ala184, Thr187, and Tyr403, as well as π-sigma with Trp422 and π-alkyl interactions with Trp422 and Tyr426.

### Filarial acetylcholine analogs impact the host lymphatics

**Fig. 7:**
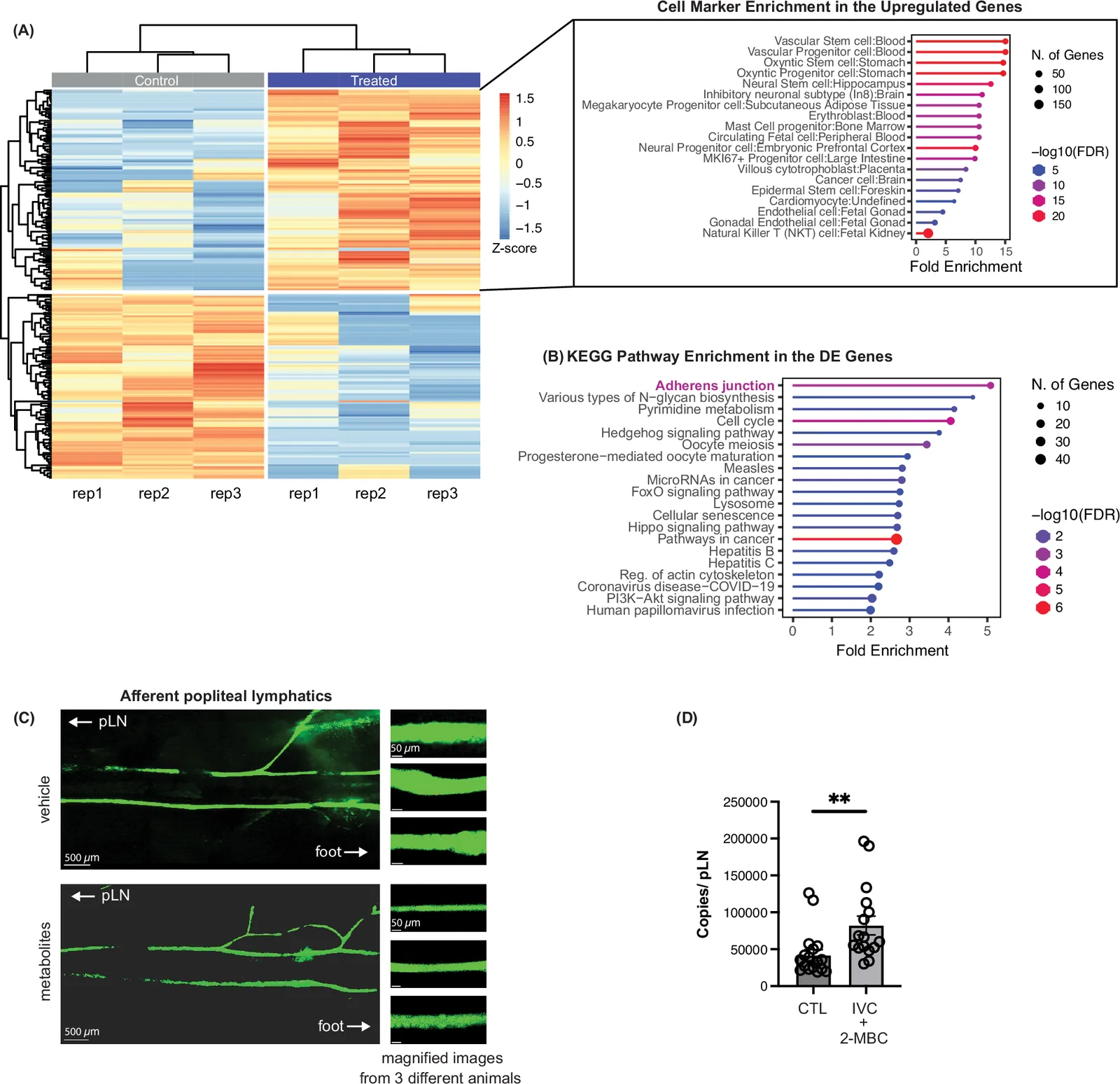
Filarial valerylcholines impact the lymphatics. **A** Heatmap of differentially expressed genes between control and treated LECs (*n* = 3) (FDR < 0.05); genes are plotted using *z*-score normalized variance-stabilized gene counts. (inset) Cell marker enrichment results in the upregulated genes using a one-tailed hypergeometric test, plotted by fold enrichment and −log_10_(FDR); point size indicates the number of genes with that cell marker in the set. **B** KEGG enrichment results for all differentially expressed genes using a one-tailed hypergeometric test, plotted by fold enrichment and −log_10_(FDR), point size indicates the number of genes with that KEGG term in the set. **C** Confocal microscope images of lymphatic vessels leading from the footpad to the popliteal lymph node of juvenile mice. Mice were injected once daily for 5 days in the hind footpad with vehicle control or isovalerylcholine (IVC) and 2-methylbutyryl choline together at 100 µM (*metabolites*). On day 5 post-treatment, mice were injected in the hind footpad with 5 µL of a 500 KDa solution of FITC dextran. SP8 confocal microscope (Leica Microsystems) using a 10x objective. Zoom-in from 3 representative mice (from *n* = 8) is shown here for each condition. Scalebars show microns. **D** At day 5, 2 h following the final metabolite injection, mice (5 mice for the control group; 4 mice for the treatment group) were inoculated with 10^4^ focus-forming units of ZIKV subcutaneously in the same hind footpad. RNA was extracted from resected popliteal lymph nodes (pLN; *n* = 17 from control mice, *n* = 16 from treatment mice) and copy numbers of the ZIKV capsid gene were assessed by qPCR. The bar plot indicates the mean ZIKV capsid gene numbers across pLNs, ±standard error. Statistical difference was determined using a two-tailed Mann–Whitney *U* test. **p = 0.0015. CTL = control. Source data are provided as a Source Data file.

To understand the transcriptional effects of these filarial metabolites on confluent human lymphatic endothelial cells (LECs), we treated cells with metabolites at a final concentration of 20 µM in the culture medium for 2 days and then measured the effect on LEC transcriptomes. We found 896 differentially expressed genes (483 upregulated, 413 downregulated) upon treatment with the metabolites (FDR < 0.05). A cell marker enrichment test of upregulated genes upon treatment using a one-tailed hypergeometric test revealed that the most enriched cell markers were for vascular stem cells and vascular progenitor cells (FDR = 8.34E−24) (Fig. 7A). We performed a KEGG enrichment analysis on differentially expressed genes using a one-tailed hypergeometric test and found the most enriched KEGG pathway to be Adherens junction (hsa04520, FDR = 0.00034). Adherens junctions are complexes that occur at cell-cell junctions to link cells and thereby preserve tissue integrity (Fig. 7B). This KEGG term was also significantly enriched in both the up- and downregulated genes when tested independently (Supplementary Data 10).

These analyses suggested that IVC and 2-MBC might affect the lymphatic epithelium. To test this hypothesis in vivo, we used a mouse model. Interestingly, mouse collecting lymphatic vessels express the M2 acetylcholine receptor and contract in response to acetylcholine^42^. We tested the impact of IVC and 2-MBC on collecting lymphatics draining to the popliteal lymph node in juvenile C57BL/6 mice. Mice received a daily footpad injection of 5 µL of a 100 µM solution of 2-MBC and IVC for 5 days. On day 5, mice were injected in the same footpad with 5 µL of 500,000 KDa FITC-dextran. After 15 min, lymphatic vessels were microsurgically exposed and visualized by confocal microscopy (Fig. 7C). Under these conditions, afferent lymphatic vessels appear structurally intact after treatment and show no obvious increase in permeability. However, lymphatic vessels appeared visibly thinner in diameter compared to vehicle-treated controls (Fig. 7C).

Lymph flow is regulated by several factors, including vessel contraction and flow resistance^43^; a decrease in diameter could therefore enhance transport to the draining lymph node. To test whether filarial valerylcholines affect lymph transport in vivo, we quantified the movement of a ~50 nm viral particle (Zika virus, ZIKV) from the footpad to the popliteal lymph node (Fig. 7D). We previously showed that 10⁴ focus-forming units (FFU) of ZIKV are efficiently captured in the popliteal LN^44^. Mice were treated with the valerylcholines for 5 days, followed by footpad inoculation with 10⁴ FFU of ZIKV. Ten minutes later, mice were euthanized, and the draining popliteal LN was harvested for viral genome quantification by qPCR. Filarial metabolite treatment significantly increased viral RNA levels in the lymph node, approximately doubling the number of viral copies from ~4 × 10⁴ to ~8 × 10⁴ (two-tailed Mann–Whitney *U* test, *p* = 0.0015). These results indicate that filarial valerylcholines, as acetylcholine analogs, can directly impact lymphatic transport in vivo in mice.

## Discussion

Genomic and proteomic studies have provided valuable insights into helminth biology; however, metabolomics remains a relatively underutilized approach in parasitology. As the most downstream output of cellular processes, the metabolome provides a dynamic and functional perspective on biochemical pathways that may not be fully captured through genomic or proteomic analyses alone. Studying the metabolomes of parasitic worms is particularly important because these organisms have co-evolved with their hosts for extended periods, developing intricate biochemical adaptations that both facilitate the parasite’s life cycle and modulate the host’s response to infection.

Through untargeted metabolomics of whole worms across the *B. malayi* life cycle and their excretory/secretory (ES) products, we identified an array of previously uncharacterized metabolites. From this collection, our analysis revealed *Brugia* male-specific metabolites significantly enriched in ES products, distinguishing them from female worms and other life stages. The structural characterization of these metabolites demonstrated their similarity to acetylcholine, but with extended aliphatic tails derived from branched-chain amino acids. Notably, metabolic labeling experiments confirmed that leucine and isoleucine are direct precursors for IVC and 2-MBC biosynthesis, suggesting a conserved but functionally divergent cholinergic signaling pathway in filarial worms. The discovery of these metabolites raises intriguing questions about their physiological roles, including their potential impact on parasite survival, tissue migration, mating behavior, and immune modulation of the host.

The male-specific production and secretion of 2-MBC and IVC in *Brugia* worms may reflect the distinct behavioral requirements of male lymphatic filariasis worms. A histological study of lymphatic nodules and vessels in *W. bancrofti*-infected individuals showed a ~5:1 female:male ratio and sparsely populated nests, including nests composed solely of females, all of which fertilized^45^, suggesting that the males were nomadic. Given that these metabolites alter lymphatic endothelial cell integrity and trigger vessel constriction, they may facilitate male movement through host tissues by creating temporary vascular changes that enable transit through the lymphatic network. This sex-specific metabolic strategy could represent an adaptation that balances the energetic cost of metabolite production against the reproductive necessity of male mobility. In *A. viteae*, we observe the opposite phenomenon where production of the valerylcholines is predominantly female-specific. *A. viteae* can infect various rodent species; adult worms reside in the subcutaneous tissue^46^ but can also be found in the muscular fascia^47^, thus smooth muscle contraction due to the valerylcholines could be important for parasite interactions, although it is not clear why female worms would be the predominant producers of these acetylcholine analogs.

Our findings indicate that filarial acetylcholine analogs regulate reproductive processes in nematodes. Treatment of *B. malayi* female worms with 2-MBC and IVC significantly reduced microfilarial production, and similar effects were observed in *C. elegans*, where these metabolites decreased egg-laying rates. These data suggest a conserved role for these valerylcholines in male reproductive fitness, as mating cannot take place when females are producing microfilariae, and these reproductive signaling pathways appear conserved across nematodes. Intriguingly, treatment with 2-MBC and IVC reduced microfilarial production by day 1 of treatment, a faster effect than we have observed for any other inhibitors. The identification of filarial acetylcholine analogs as inhibitors of parasite reproduction suggests potential targets for therapeutic intervention. Additionally, we demonstrated that doxycycline treatment, which eliminates *Wolbachia* endosymbionts, significantly increased the levels of 2-MBC and IVC (and their precursors), suggesting that bacterial endosymbionts might modulate the synthesis or stability of these metabolites. Conversely, ethacrynic acid, an inhibitor of choline acetyltransferase, suppressed 2-MBC and IVC production and impaired worm viability. These findings implicate cholinergic pathways as promising targets for novel anthelmintics that disrupt filarial reproduction and viability.

Functional assays revealed that both 2-MBC and IVC selectively activate the mammalian muscarinic acetylcholine receptor M2, a key regulator of smooth muscle contraction and immune responses^48^. Structural docking studies indicated that these metabolites likely exhibit distinct binding interactions with *B. malayi* GAR-2 receptors compared to human M2 receptors. The differential activation of host versus parasite cholinergic receptors raises the intriguing possibility of pharmacology that selectively targets *B. malayi* receptors. Moreover, the valerylcholines showed higher affinity for the *B. malayi* receptor than canonical acetylcholine, but equal affinity as acetylcholine for the human receptor, potentially indicating that the valerylcholines are a way for the *Brugia* males to modulate female reproduction.

In the host, muscarinic acetylcholine receptors regulate vascular tone and lymphatic contraction^49^. Our studies show that, indeed, *B. malayi* acetylcholine analogs affect lymphatics and increase flow to draining lymph nodes. A possibility is that activation of host M2 receptors by filarial acetylcholine analogs may directly contribute to the lymphatic dysfunction observed in infected individuals. Our transcriptomic analysis of lymphatic endothelial cells exposed to 2-MBC and IVC revealed significant downregulation of genes involved in focal adhesion and adherens junction pathways. These findings suggest that the valerylcholines may disrupt endothelial integrity, potentially facilitating parasite persistence within the host lymphatic system. This aligns with prior observations that filarial infections lead to endothelial dysfunction, lymphatic remodeling, and, ultimately, lymphedema^4,50^. The ability of these metabolites to perturb cell-cell adhesion and vascular stability may represent a mechanism by which filarial worms evade host defenses and establish chronic infections.

In conclusion, this study identifies two novel biological acetylcholine analogs from filarial worms that influence both parasite physiology and host endothelial function. These findings provide mechanistic insights into how *B. malayi* may exploit cholinergic signaling to modulate host endothelial integrity and maintain chronic infections, while providing an advantage in male reproductive fitness. Future studies should explore the broader effects of these metabolites on immune cell populations, endothelial barrier integrity, and lymphatic function. Additionally, the potential for targeting filarial cholinergic pathways as a therapeutic strategy warrants further investigation, such as the FDA-approved drug ethacrynic acid, particularly in the development of selective inhibitors that disrupt parasite-specific metabolic and signaling pathways while sparing host physiology. The discovery of filarial acetylcholine analogs as mediators of host-parasite interactions represents a significant advancement in our understanding of filarial metabolism and pathogenesis. By elucidating their biosynthesis, function, and impact on host tissues, we pave the way for novel therapeutic approaches aimed at mitigating filarial diseases and improving global health outcomes. This also opens avenues for exploring other possible variants of these molecules across biological systems^51^.

## Methods

### Parasite preparations

All parasites were provided by the NIH/NIAID Filariasis Research Reagent Resource Center for distribution through BEI Resources (Manassas, VA, USA). Worms were flash-frozen and shipped, or were sent fresh for in vitro culture experiments. Stages used for metabolomics analysis of whole worms included *Brugia malayi* L3 larvae from mosquitoes (*Aedes aegypti*), *Brugia malayi* microfilaria from infected gerbils (*Meriones unguiculatus)*, as well as *B. malayi, B. pahangi*, and *Acanthocheilonema viteae* adult worms at 120 dpi from infected gerbils.

The number of *B. malayi* worms per sample for whole worm lysis was 20 adult female worms, 40 adult males, 2 × 10^6^ microfilariae, and 200 L3 larvae per biological replicate (*n* = 3). Samples were washed in 1x PBS; adult male and female worms were picked individually from PBS, and each biological replicate was weighed. The microfilariae and L3 samples were spun down, the PBS was pipetted off, and the bulk worms were weighed.

### Excretory/secretory products

To collect ES products, cultures of adult worms (*B. malayi*, *B. pahangi*, or *A. viteae*) were generally done with 2 worms per 2 ml of culture medium without FBS (RPMI-1640, 100 U/mL penicillin, 100 mg/mL streptomycin, 2 mM L-glutamine, and glucose at a concentration of 2%). Cultures were done in triplicate (*n* = 3) for *B. malayi* and *A. viteae*, and six times (*n* = 6) for *B. pahangi*. The next day, the media was collected and spun down (300 × *g* for 5 min) to remove mf released from females, and then the supernatant was passed through 0.22 µ filters, aliquoted, and snap-frozen in an ethanol/dry ice bath. For experiments quantifying metabolites in the spent media of *B. malayi* (Fig. 2C), 1 to 4 worms were cultured per sample; These culture experiments were performed in triplicate.

### Metabolomics

All metabolomics samples, unless otherwise specified, were analyzed with a reverse-phase LC chemistry and high-resolution MS, adapted as previously described^52^. Briefly, LCMS parameters were adapted from a previously described method. The LC column was a Waters^TM^ BEH-Phenyl (2.1 × 150 mm, 1.7 μm) coupled to a Dionex Ultimate 3000^TM^ system, and the column oven temperature was set to 25 °C for the gradient elution. A flow rate of 200 μL/min was used with the following buffers: (A) 0.1% formic acid in water, and (B) 0.1% formic acid in acetonitrile. The gradient profile was as follows; 0–35%B (0–10 min), 35–75%B (10–15 min), 75–99%B (15–15.25 min), 99–99%B (15.25–16.5 min), 99–0%B (16.5–16.75 min), 0–0%B (16.75–20 min). Injection volume was set to 2 μL for all analyses (20 min total run time per injection). Some samples were analyzed with an additional LC method using a MilliporeTM ZIC-pHILIC (2.1 × 150 mm, 5 μm) column coupled to a Dionex Ultimate 3000TM. A flow rate of 100 μL/min was used with the following buffers: (A) 10 mM ammonium carbonate in water, pH 9.0, and (B) neat acetonitrile. The gradient profile was as follows: 80–20%B (0–30 min), 20–80%B (30–31 min), 80–80%B (31–42 min). Injection volume was set to 2 μL for all analyses (42 min total run time per injection). All MS analyses were carried out by coupling the LC system to a Thermo Q Exactive HF^TM^ mass spectrometer operating in heated electrospray ionization mode (HESI). Method duration was 20 min with a polarity switching data-dependent Top 5 method for both positive and negative modes. Spray voltage for both positive and negative modes was 3.5 kV, and capillary temperature was set to 320 °C with a sheath gas rate of 35, aux gas of 10, and max spray current of 100 μA. The full MS scan for both polarities utilized 120,000 resolution with an AGC target of 3e6 and a maximum IT of 100 ms, and the scan range was from 95–1000 *m*/*z*. Tandem MS spectra for both positive and negative mode used a resolution of 15,000, AGC target of 1e5, maximum IT of 50 ms, isolation window of 0.4 *m*/*z*, isolation offset of 0.1 *m*/*z*, fixed first mass of 50 *m*/*z*, and 3-way multiplexed normalized collision energies (nCE) of 10, 35, 80. The minimum AGC target was 1e4 with an intensity threshold of 2e5. All data were acquired in profile mode. For targeted MS2 analyses of the valerylcholines, including quantification, the same LC chemistry and gradient were used, but a dedicated PRM scan for the parent mass at 188.1640 *m*/*z* was introduced, and the topn loop count was reduced to 1 to provide more duty cycle and ion time for the targets. PRM scans were performed at 15,000 resolution with a 100 ms max ion time.

Metabolites were extracted from all samples after scaling to a uniform concentration in a resulting 80% acetonitrile with 500 nM metabolomics amino acid mix standard (catalog no. MSK-A2-1.2, Cambridge Isotope Laboratories) added as an internal control. For media samples, extraction solution was added to samples at a ratio of 200 µL sample/1 mL extraction solution. For intact worm samples (adults, L3, mf), the metabolite extraction was scaled to 16.5 mg worm material/1 mL extraction solution in 80% acetonitrile. This material mass corresponded to ~ 2e6 counts for microfilaria (mf), ~500 L3, 35 adult females, and 340 adult males. All samples, including media, were subjected to mechanical homogenization and extracted in 2.0 mL screw cap vials containing ~100 µL of disruption beads (Research Products International, Mount Prospect, IL). Each sample was homogenized for 10 cycles on a bead blaster homogenizer (Benchmark Scientific, Edison, NJ) in a 4 °C cold-room with dry-ice chilled extraction solution. Each cycle consisted of a 30 s homogenization time at 6 m/s followed by a 30 s pause. Samples were subsequently centrifuged at 21,000 × *g* for 3 min at 4 °C. A fixed volume of 450 µL from the homogenized samples was transferred to a 1.5 mL tube and dried down by speed vacuum concentration (Thermo Fisher, Waltham, MA) to ensure equal amounts of sample represented for relative quantification experiments. Samples were reconstituted in 50 µL of Optima LC/MS grade water (Fisher Scientific, Waltham, MA). After reconstitution, samples were sonicated for 2 min, then centrifuged at 21,000 × *g* for 3 min at 4 °C. From each sample, 20 µL of final extract was transferred to an LC vial containing glass inserts for LC injection. The remaining sample was placed in −80 °C for long-term storage.

For each LCMS dataset, quality control was assessed at several levels. First, replicate injections of neat internal standards before and after all study injections were assessed for stability of instrument response and chromatographic stability. Next, we quantified the list of doubly labeled amino acids (MSK-A2-1.2) that were added as internal standards to the samples. Absolute instrument mass accuracy was required to be <5 ppm for all studies, retention time performance was required to be <0.5 min (RT range: earliest to latest peak within a sequence), and internal standard response variability was required to be <15% across the spiked samples (median coefficient of variation).

Metabolite peaks were putatively identified by spectral library search against the NIST17MS/MS and METLIN tandem mass spectral libraries of metabolites. The top-scoring structure match for each data-dependent spectrum was returned using an in-house script wrapper “Dewey” for MSPepSearch_x64. Putative structures were sorted from high to low by their reverse dot scores, and duplicate structures were discarded, retaining only the top-scoring MS2 spectrum and the neutral chemical formula and detected polarity (+ or −) of the putative metabolite. These chemical formulas were used to quantify the extracted ion chromatograms using the theoretical *m*/*z* of the library-annotated ion type, 10 ppm, using an in-house script. Relative quantification of the peaks was carried out and normalized to the median peak intensity for each sample. Top hits were manually inspected and visualized. Untargeted metabolite features were detected with an in-house script “Ungrid”, resulting in >20,000 raw features. These features were filtered for minimum signal intensity (1e5) (a unique *m*/*z* at a given retention time 5 ppm, 0.5 min window). These *m*/*z* values and retention times were used to quantify the extracted ion chromatograms, 10 ppm tolerance, using an in-house script “Skeleton”. Downstream statistics were performed with the “Metabolyze” pipeline for groupwise comparisons. All scripts available on GitHub (https://github.com/NYUMetabolomics/plz). All statistics on the valerlycholines in targeted experiments were performed in GraphPad Prism, as annotated in specific results and figure legends.

### Molecular networking

The molecular network was run using the GNPS (Global Natural Products Social Molecular Network) platform. Feature-based molecular networks were generated^53^. Links to all jobs can be found in the “Data availability” section. Both the conventional and molecular community networks^54^ were generated. For the latter, an unpruned network was generated as follows: The precursor ion mass tolerance was set to 0.1 DA, while the fragment ion mass tolerance was set to 0.1 DA. The min pairs cosine score was set to 0.01, the minimum matched fragment ions was set to 4, while the network topk was set to 4. The rest of the settings remained the same as for the conventional network. The graphml file for the unpruned network was then used to generate a molecular community network as described in ref. ^54^.

A molecular community network was generated for experimental spectra with reference spectra of acetylcholine from the public library available at the GNPS platform to confirm the connectivity of the valerylcholines to structurally similar acetylcholine in a molecular network; the reference spectra were downloaded from GNPS and co-networked with the experimental spectra. The unpruned network was generated with the following settings: the precursor ion mass tolerance was set to 0.02 Da, and the fragment ion mass tolerance was set to 0.02 Da. The min cosine pairs were set to 0.01, the minimum matched fragment ions were set to 3, while the network topK was set to 10,000, and cluster size was set to unlimited. The graphml file for the unpruned network was then used to generate a molecular community network by the MCN generation code available at GitHub: https://github.com/Alexander0/molecular_communities.

### Synthesis of metabolites

All reagents and solvents were purchased from commercial sources (Sigma-Aldrich, TCI Europe N.V., Strem Chemicals, etc.) and were used without further purification unless otherwise noted. Reactions were monitored by TLC on pre-coated, Merck Silica gel 60 F_254_ glass-backed plates and the chromatograms were visualized by staining with aqueous ceric ammonium molybdate solution, and gentle heating with a heat gun. Flash silica gel chromatography was performed using silica gel (SiO^2^, particle size 40–63 μm) purchased from Merck. All NMR spectra (Supplementary Methods) were measured on a BRUKER Avance III HD 400 (equipped with a CryoProbe^TM^). A Shimadzu TQ 8040 GC-MS/FID system with electron ionization (EI) ion source was used to obtain low-resolution mass spectra (LRMS). High-resolution mass spectra (HRMS) were obtained with an Agilent 6224 Accurate Mass time-of-flight (TOF) LC/MS system using either an electrospray ionization (ESI) or atmospheric pressure chemical ionization (APCI) ion source. All reported data refer to the positive ionization mode. Synthetic schemes provided as Supplementary Methods.

### Validation of metabolites using whole worms

Intact worm samples were extracted as previously. Briefly, a count of 10 *B. malayi* worms per sample was homogenized with 1 mL of 80% methanol with a mixture of glass and stainless-steel beads in a bead blaster tube (20 cycles at 6 m/s 30 on and 30 off). A standard curve of 2-methylbutyryl choline (2-MBC) and isovalerylcholine (IVC) was prepared from authentic standards (Trauner lab) and processed in the same fashion, side-by-side with the worm samples. The resulting lysates were centrifuged to clarity (21,000 *g* ×3 min), and an aliquot (360 μL) was dried by speed vacuum and reconstituted in water (40 μL), then transferred to LCMS vials for analysis. The hydrophobic platform was used for chromatography, and a dedicated product reaction monitoring scan (PRM) was used for MS detection to target both 2-MBC and IVC (188.1645 *m*/*z* ± 0.2 *m*/*z* isolation window).

### Biosynthetic pathway tracing with C13-labeled metabolites

To characterize the biosynthetic pathway of IVC and 2-MBC in *B. malayi* adult worms, we performed live worm culture with uniformly carbon-13-labeled leucine and isoleucine. Culture conditions were tested with different combinations of labeled and unlabeled isoleucine and leucine. For these experiments, adult males were cultured in vitro in groups of 3 worms per well, and cultures were run in biological duplicates. Isoleucine and leucine (either labeled or unlabeled) were added to culture medium without FBS (RPMI-1640, 100 U/mL penicillin, 100 mg/mL streptomycin, 2 mM L-glutamine, and glucose at a concentration of 2%) for a final concentration of 50 mg/L. The worms were cultured overnight, and 1 ml of spent media was collected after 24 h of culture, passed through a 0.22 µm filter, and frozen. Standard media as control were included. All samples were snap-frozen and sent for metabolomics analysis. The culture experiment was done in duplicate.

### Doxycycline and ethacrynic acid treatments

For the doxycycline treatment experiments, adult male *B. malayi* worms (more than 120 days post-infection) were placed in 5 mL of complete culture medium (RPMI-1640 supplemented with 10% FBS, 100 U/mL penicillin, 100 mg/mL streptomycin, and 2 mM L-glutamine), 10 worms per well in a 6-well plate, and incubated at 37 °C under 5% CO_2_ conditions. The parasites were treated with doxycycline (12.5 µM) for 3 days in vitro. Control worms were similarly incubated in the medium containing an equivalent volume of DMSO (0.1% v/v) for 3 days. Culture experiments were done in triplicate. After incubation, the worms were washed three times with PBS, snap-frozen in liquid nitrogen, and stored at −80 °C for subsequent metabolomics analysis.

For the ethacrynic acid treatment experiments, worms were cultured in the same conditions, 10 worms per well, with 5 µM or 50 µM of ethacrynic acid added to the culture medium without FBS but supplemented with 2% glucose. After 24 h, 1 ml of spent media was collected, passed through a 0.22 µm filter, and frozen. Standard medium was used as a control, and all samples were snap-frozen and sent for metabolomics analysis. The culture experiment was done in duplicate.

### Transcriptomics experiments

Human lymphatic endothelial cells (LECs) HMVEC (Lonza, Catalog #: CC-2543) seeded at 3000 cells/cm^2^ were cultured at 37 °C and 5% CO_2_ in 75-cm^2^ flasks containing complete tissue culture medium (Microvascular Endothelial Cell Growth Medium-2, EGM-2, Lonza). The cell line was not authenticated in our laboratory. Cell culture medium was changed routinely every 2–3 days, and cells were transferred to a new flask every 5–7 days. IVC and 2-MBC were added (20 µM) to the culture medium when cells reached confluence, and total RNA was extracted 2 days later from the cells using the RNeasy Mini Spin Kit (Qiagen) following manufacturer instructions. Library construction for the mRNA was prepared using the NEBNext Ultra II Directional RNA Library Prep Kit in conjunction with the NEBNext Poly(A) mRNA Magnetic Isolation Module (New England Biolabs). Libraries were pooled, diluted, and quality-checked according to manufacturer specifications before loading onto a NextSeq 500 sequencer using a High Output Kit v2.5 (300 cycles) (Illumina).

For control and IVC + 2-MBC-treated male and female *B. malayi* worms, raw sequencing reads from each sample were processed using Fastp (version 0.23.4)^55^ and were quantified using Salmon (version 1.10.2)^56^, using the reference genome for *B. malayi* PRJNA10729 WS290 from Wormbase. Read counts were used for differential expression analysis, conducted with DESeq2 (version 4.0.3)^57^. Differentially expressed genes were determined between control and treated worms for both male and female worms. Genes were considered differentially expressed at a Benjamini–Hochberg adjusted *p* value (FDR) < 0.05, the default threshold in DESeq2. Volcano plots were generated by plotting log_2_(Fold Change) against −log_10_(FDR). Gene Ontology (GO) enrichment analysis was performed using g:Profiler (version: *e113_eg59_p19_f6a03c19*). Over-representation was assessed using a one-tailed hypergeometric test, and multiple hypothesis testing was corrected using the g:SCS (set counts and sizes) procedure implemented in g:Profiler. GO terms with an adjusted *p* value < 0.05 were considered significantly enriched^58^.

For treated and control LECs, raw sequencing reads from each sample were processed similarly as above using Fastp and were quantified using Salmon, using the reference genome assembly for Homo sapiens GRCh38.p14 from NCBI. Differentially expressed genes were determined as above using DESeq2. Following a variance-stabilizing transformation, expression values for each gene were used for generating heatmaps with Pretty Heatmaps (version 1.0.12). GO term, KEGG, and cell marker enrichment were performed using one-tailed hypergeometric tests in ShinyGO (version 0.8)^59^.

### *C. elegans* egg laying assay

Wild-type *C. elegans* Bristol strain N2 was maintained on nematode growth medium (NGM) plates seeded with *E. coli* OP50 bacteria at 20 °C, as described previously^60^. Five 1-day-old animals were placed in each well of a 96-well microtiter plate with 100 μL of S basal buffer and exposed to vehicle (1% DMSO) or vehicle plus chemicals of interest at a final concentration of 10 μM. After dosing the animals for 2 h, the released eggs were counted using a standard dissection stereomicroscope (Olympus, Zeiss). Differences in released eggs between the DMSO control and each of the metabolites were analyzed using a one-way ANOVA, followed by Holm-Šídák’s multiple comparisons test, with adjusted *p* values.

### Quantification of microfilariae released by female worms

We tested the capacity of the valerylcholines secreted by male *Brugia* worms to impact female worm motility and their capacity to produce offspring (microfilariae, mf). Thus, we quantified the number of mf released by adult female *B. malayi* during the initial 3 days of treatment with the acetylcholine analogs. A concentration of 10 µM of each compound was used to treat 6–7 pairs of females in 6-well plates. Control worms were treated with DMSO (0.1% v/v). The medium was replaced with fresh compounds every 24 h, and the medium containing mfs was used to count the number of mfs released during each treatment period (24 h). Aliquots (20 µL) of the medium with mf were taken from each well (containing 2 females) for counting mf using a dissection stereomicroscope (Olympus, Zeiss). These numbers were then used to determine the total number of mfs per female. Statistical significance was determined using an unpaired *t*-test.

### PRESTO-Tango assay

HTLA cells–a human embryonic kidney 293 (HEK293) cell line stably expressing a tetracycline transactivator–dependent luciferase reporter and a β-arrestin2–tobacco etch virus fusion gene^61^ (cell line obtained from Aashish Manglik at UCSF; not authenticated in our laboratory) were maintained in DMEM containing 10% FBS, 1% Penicillin/Streptomycin, 2.5 ug/mL and 75 ug/mL hygromycin B. For Tango assays, HTLA cells were seeded in DMEM containing 10% FBS 1% Penicillin/Streptomycin. A total of 2.0 × 10^4^ cells were plated in a 96-well white plate 16 h before transfection. A total of 150 ng per well of plasmids (CHRM1-5-Tango) were mixed with the transIT-293 transfection reagent for 20 min before transfection. After 20–24 h transfection, each compound (IVC, 2-MBC, or a combination of both IVC + 2-MBC) resuspended in DMSO was added at 20 µM and incubated for another 20–24 h. The control was done with DMSO only. The culture media was discarded and 50 μl per well of Bright-Glo solution (Promega) diluted 20-fold with PBS containing 20 mM HEPES was added into each well. Luminescence was measured using Biotek Synergy H1 (BioTek Instruments, Winooski, VT). To calculate fold change, wells without compounds and DMSO only were used as “basal”. Differences in fold change between muscarinic receptors and between treatments were analyzed using one-way ANOVA, followed by Tukey’s HSD multiple comparisons test, with adjusted *p* values (*p*.adj) used for significance thresholds. All experiments were done in triplicate; the experiment with CHRM2 only was repeated for confirmation.

### Structural predictions of binding affinity and molecular interactions

The amino acid sequence of muscarinic acetylcholine receptor M2 (*Brugia malayi*, ID: A0A8L7YLR4) was retrieved from UniProt (https://www.uniprot.org/). Due to the absence of a crystallized 3D structure, the SWISS-MODEL server^62^ was utilized to generate its model based on template IDs: 5zkb and 8e9z. The human muscarinic acetylcholine receptor M2 was retrieved from the Protein Data Bank (PDB ID: 5YC8)^63^. The chemical structures of acetylcholine, 2-methylbutyryl choline, and isovalerylcholine were generated using ChemDraw (saved in SDF format). Compounds were energy-minimized using molecular mechanics force fields (MMFF94) to ensure they were in the 3D structure at the lowest energy conformation. Molecular docking compatible PDBQT files of compounds and receptors were generated. Blind docking (search space box size of 150 Å) was run on the NIH HPC Biowulf server using Auto Dock Vina. The binding poses and binding energies were evaluated. Compound-protein complex structures were generated using PyMol (v3.1.0), and two-dimensional molecular interaction networks were drawn with Discovery Studio (v2023).

### Mouse lymphatic experiments

Female C57BL/6 mice from 6–10 weeks of age were injected once daily for 5 days in the hind footpad with 5 µL of vehicle control or both metabolites to a concentration of 100 µM. Two hours following the final metabolite injection, mice were inoculated with 10^4^ focus-forming units of ZIKV (strain H/PF/2013) in 20 µL total volume subcutaneously in the same hind footpad. Mice were euthanized 10 min following inoculation and popliteal lymph nodes were resected and stored in RNALater solution. RNA was extracted using the RNeasy mini kit (Qiagen), cDNA was made using the High-Capacity cDNA Reverse Transcription kit (Thermo Fisher), and copy numbers of the ZIKV capsid gene were assessed by qPCR using the PowerUp SYBR Green Master Mix (Thermo Fisher) on a QuantStudio 3 (Thermo Fisher). The statistics were assessed using a two-tailed Mann–Whitney *U* test. The sequence for the ZIKV capsid primers is as follows: Forward- GGAAAAAAGAGGCTATGGAAATAATAAAG; Reverse- CTCCTTCCTAGCATTGATTATTCTCA. The experiment was repeated twice with 4–5 mice per group.

For imaging mouse lymphatics, mice received the same treatment regimen as above. On day 5 post-treatment, mice were injected in the hind footpad with 5 µL of a 500 KDa solution of FITC dextran (1 mg/ml). Ten minutes later, mice were anesthetized using Avertin and the skin was surgically removed from the hindlimb using a dissecting microscope to avoid damaging blood or lymphatic vessels. The anesthetized mouse was placed in a 100 mm dish with a glass coverslip insert (Mattek corporation), PBS was applied to keep the leg moist, and the lymphatic vessels leading from the footpad to the popliteal lymph node were imaged on a SP8 confocal microscope (Leica Microsystems) using a 10x objective. Tiled images were merged and analyzed using Imaris software version 10.2.0 (Oxford Instruments). A total of 8 mice were imaged for each condition. Scalebars show microns.

All animal experiments were conducted in accordance with the recommendations in the Guide for the Care and Use of Laboratory Animals of the National Institutes of Health. The NIAID Division of Intramural Research Animal Care and Use Committee approved all animal work performed in this study. The NIH is fully accredited by AAALAC International, and all animal procedures were conducted under PHS Assurance number D16-00602 (A4149-01). All animals were socially housed in individually ventilated solid-bottom cages on hardwood bedding (Sani-Chips, ASAP) that had a relative negative-pressure micro-environment to the surrounding room. Mice were maintained on a 12:12-h light:dark cycle, at 72 ± 3 °F ambient temperature, and 30-70% relative humidity.

Supplementary Table 1 lists details on the experimental design for each section of the Results.

### Reporting summary

Further information on research design is available in the Nature Portfolio Reporting Summary linked to this article.

## Supplementary information

Supplementary Information

Supplementary Data 1

Supplementary Data 2

Supplementary Data 3

Supplementary Data 4

Supplementary Data 5

Supplementary Data 6

Supplementary Data 7

Supplementary Data 8

Supplementary Data 9

Supplementary Data 10

Reporting Summary

Transparent Peer Review file

## Source data

Source Data File

## Data Availability

The LC-MS metabolomics data generated in this study are available on Zenodo https://doi.org/10.5281/zenodo.17101533. The raw sequencing data generated for this study are available in the Sequence Read Archives under PRJNA1315212. The conventional and molecular community network GNPS jobs can be accessed at: https://gnps.ucsd.edu/ProteoSAFe/status.jsp?task=3866393dea534cdca5618a500ffcb9fe, https://gnps.ucsd.edu/ProteoSAFe/status.jsp?task=b5d3f7d60e5e49f591172826c60d7e46 and https://gnps2.org/status?task=fbfd168f6c704c9982d70548e753f686 Source data are provided with this paper.

## Code availability

Our data processing pipeline used for the metabolomics experiments can be found here: https://github.com/NYUMetabolomics/plz and here https://github.com/DrewRJones/Metabolyze_Public.
