# Supplementary material for: Secreted filarial acetylcholine analogs modulate parasite reproduction and host physiology
Source: Nat Commun. 2026 Aug 29;17:10336. doi: 10.1038/s41467-026-77090-2 (PMC13623875; doi:10.1038/s41467-026-77090-2)
Supplement: Supplementary file 1 [file 41467_2026_77090_MOESM12_ESM.pdf]

nature portfolio

Corresponding author(s): Elodie Ghedin

Last updated by author(s): 8/7/2026

Reporting Summary

Nature Portfolio wishes to improve the reproducibility of the work that we publish. This form provides structure for consistency and transparency in reporting. For further information on Nature Portfolio policies, see our Editorial Policies and the Editorial Policy Checklist.

Statistics

For all statistical analyses, confirm that the following items are present in the figure legend, table legend, main text, or Methods section.

n/a	Confirmed	
☐	☒	The exact sample size (*n*) for each experimental group/condition, given as a discrete number and unit of measurement
☐	☒	A statement on whether measurements were taken from distinct samples or whether the same sample was measured repeatedly
☐	☒	The statistical test(s) used AND whether they are one- or two-sided<br>*Only common tests should be described solely by name; describe more complex techniques in the Methods section.*
☒	☐	A description of all covariates tested
☐	☒	A description of any assumptions or corrections, such as tests of normality and adjustment for multiple comparisons
☐	☒	A full description of the statistical parameters including central tendency (e.g. means) or other basic estimates (e.g. regression coefficient) AND variation (e.g. standard deviation) or associated estimates of uncertainty (e.g. confidence intervals)
☐	☒	For null hypothesis testing, the test statistic (e.g. *F*, *t*, *r*) with confidence intervals, effect sizes, degrees of freedom and *P* value noted<br>*Give P values as exact values whenever suitable.*
☒	☐	For Bayesian analysis, information on the choice of priors and Markov chain Monte Carlo settings
☒	☐	For hierarchical and complex designs, identification of the appropriate level for tests and full reporting of outcomes
☐	☒	Estimates of effect sizes (e.g. Cohen's *d*, Pearson's *r*), indicating how they were calculated

Our web collection on statistics for biologists contains articles on many of the points above.

Software and code

Policy information about availability of computer code

Data collection

Metabolic data: Metabolite peaks were putatively identified by spectral library search against the NIST17MS/MS and METLIN tandem mass spectral libraries of metabolites. The top scoring structure match for each data-dependent spectrum was returned using an in-house script wrapper “Dewey” for MSPepSearch_x64.
Structural predictions of binding affinity and molecular interactions: The amino acid sequence of muscarinic acetylcholine receptor M2 (Brugia malayi, ID: A0A8L7YLR4) was retrieved from UniProt (https://www.uniprot.org/). Due to the absence of a crystallized 3D structure, the SWISS-MODEL server 52 was utilized to generate its model based on template IDs: 5zkb and 8e9z. The human muscarinic acetylcholine receptor M2 was retrieved from Protein Data Bank (PDB ID: 5YC8). The chemical structures of acetylcholine, 2-methylbutyrylcholine and isovalerylcholine were generated using ChemDraw (saved in SDF format). Compounds were energy-minimized using molecular mechanics force fields (MMFF94) to ensure they were in the 3D structure at the lowest energy conformation. Molecular docking compatible PDBQT files of compounds and receptors were generated. Blind docking (search space box size of 150 Å) was run on the NIH HPC Biowulf server using Auto Dock Vina.

Data analysis

Data analysis of metabolic data was performed with custom scripts available at https://github.com/NYUMetabolomics/plz and https://github.com/DrewRJones/Metabolyze_Public
The molecular network was run using the GNPS (Global Natural Products Social Molecular Network) platform.
Compound-protein complex structures were generated using PyMol (v3.1.0) and two-dimensional molecular interaction networks were drawn with Discovery Studio (v2023).
For transcriptomic analysis, raw sequencing reads from each sample were processed using Fastp (version 0.23.4) and were quantified using Salmon (version 1.10.2) , using the reference genome for B. malayi PRJNA10729 WS290 from Wormbase. Read counts were used for differential expression analysis, conducted with DESeq2 (version 4.0.3). . Differentially expressed genes were determined between control

and treated worms for both male and female worms. Genes were considered differentially expressed at a Benjamini-Hochberg adjusted p-value (FDR) <0.05, the default threshold in DESeq2. Volcano plots were generated by plotting log2(Fold Change) against -log10(FDR). Gene Ontology (GO) enrichment analysis was performed using g:Profiler (version: e113_eg59_p19_f6a03c19). Over-representation was assessed using a one-tailed hypergeometric test, and multiple hypothesis testing was corrected using the g:SCS (set counts and sizes) procedure implemented in g:Profiler. GO terms with an adjusted p-value < 0.05 were considered significantly enriched.

For manuscripts utilizing custom algorithms or software that are central to the research but not yet described in published literature, software must be made available to editors and reviewers. We strongly encourage code deposition in a community repository (e.g. GitHub). See the Nature Portfolio guidelines for submitting code & software for further information.

Data

Policy information about availability of data

All manuscripts must include a data availability statement. This statement should provide the following information, where applicable:

- Accession codes, unique identifiers, or web links for publicly available datasets
- A description of any restrictions on data availability
- For clinical datasets or third party data, please ensure that the statement adheres to our policy

Our data processing pipeline used for the metabolomics experiments can be accessed here: https://github.com/NYUMetabolomics/plz
and here https://github.com/DrewRJones/Metabolyze_Public. LC-MS metabolomics data are available on Zenodo doi:10.5281/zenodo.17101533
Sequence data are available in the Sequence Read Archives under PRJNA1315212.
The conventional and molecular community network GNPS jobs can be accessed at:
https://gnps.ucsd.edu/ProteoSAFe/status.jsp?task=3866393dea534cdca5618a500ffcb9fe
https://gnps.ucsd.edu/ProteoSAFe/status.jsp?task=b5d3f7d60e5e49f591172826c60d7e46
and here
https://gnps2.org/status?task=fbfd168f6c704c9982d70548e753f686

Research involving human participants, their data, or biological material

Policy information about studies with human participants or human data. See also policy information about sex, gender (identity/presentation), and sexual orientation and race, ethnicity and racism.

Reporting on sex and gender	
Reporting on race, ethnicity, or other socially relevant groupings	*Please specify the socially constructed or socially relevant categorization variable(s) used in your manuscript and explain why they were used. Please note that such variables should not be used as proxies for other socially constructed/relevant variables (for example, race or ethnicity should not be used as a proxy for socioeconomic status).<br>Provide clear definitions of the relevant terms used, how they were provided (by the participants/respondents, the researchers, or third parties), and the method(s) used to classify people into the different categories (e.g. self-report, census or administrative data, social media data, etc.)<br>Please provide details about how you controlled for confounding variables in your analyses.*
Population characteristics	*Describe the covariate-relevant population characteristics of the human research participants (e.g. age, genotypic information, past and current diagnosis and treatment categories). If you filled out the behavioural & social sciences study design questions and have nothing to add here, write "See above."*
Recruitment	*Describe how participants were recruited. Outline any potential self-selection bias or other biases that may be present and how these are likely to impact results.*
Ethics oversight	*Identify the organization(s) that approved the study protocol.*

Note that full information on the approval of the study protocol must also be provided in the manuscript.

Field-specific reporting

Please select the one below that is the best fit for your research. If you are not sure, read the appropriate sections before making your selection.

☒ Life sciences ☐ Behavioural & social sciences ☐ Ecological, evolutionary & environmental sciences

For a reference copy of the document with all sections, see nature.com/documents/nr-reporting-summary-flat.pdf

Life sciences study design

All studies must disclose on these points even when the disclosure is negative.

Sample size	The number of B. malayi worms per sample were 20 adult female worms, 40 adult males, 2X10^6 microfilaria, and 200 L3 larvae per biological replicate. These numbers were determined by weight (adult females are 2X larger than males etc). To collect ES products, cultures of adult

	worms (B. malayi, B. pahangi, or A. viteae) were generally done with 2 worms per 2 ml of culture medium. All sample sizes for each experiment are listed in figure legends and in Supplementary Table 1.
Data exclusions	No data were excluded from the analyses.
Replication	Reproducibilty was determined by running all experiments largely in triplicate (biological replicates) with some instances where experimenst were done in duplicate or 6 times.
Randomization	Grouping was assigned based on sex of worm, or developmental stage, or by treatment/no treatment
Blinding	Blinding was not relevant to our study as study involved simple group comparisons that resulted in quantitative data

Reporting for specific materials, systems and methods

We require information from authors about some types of materials, experimental systems and methods used in many studies. Here, indicate whether each material, system or method listed is relevant to your study. If you are not sure if a list item applies to your research, read the appropriate section before selecting a response.

Materials & experimental systems

n/a	Involved in the study
☒	☐ Antibodies
☐	☒ Eukaryotic cell lines
☒	☐ Palaeontology and archaeology
☐	☒ Animals and other organisms
☒	☐ Clinical data
☒	☐ Dual use research of concern
☒	☐ Plants

Methods

n/a	Involved in the study
☒	☐ ChIP-seq
☒	☐ Flow cytometry
☒	☐ MRI-based neuroimaging

Eukaryotic cell lines

Policy information about cell lines and Sex and Gender in Research

Cell line source(s)	HTLA cells–a human embryonic kidney 293 (HEK293) cell line stably expressing a tetracycline transactivator–dependent luciferase reporter and a β-arrestin2–tobacco etch virus fusion gene51 (cell line obtained from Aashish Manglik at UCSF); Human Lymphatic Epithelial Cells were purchased from Lonza (Catalog #: CC-2543)
Authentication	none of the cell lines used were authenticated in our lab
Mycoplasma contamination	cell lines were not tested for mycoplasma
Commonly misidentified lines (See ICLAC register)	*Name any commonly misidentified cell lines used in the study and provide a rationale for their use.*

Animals and other research organisms

Policy information about studies involving animals; ARRIVE guidelines recommended for reporting animal research, and Sex and Gender in Research

Laboratory animals	Female C57BL/6 mice from 6 - 10 weeks of age ; female and male B. malayi, B. pahangi, A. viteae worms 120 days old ; C. elegans strain N2; All animals were socially housed in individually ventilated solid bottom cages on hardwood bedding (Sani-Chips, ASAP) that have a relative negative pressure micro-environment to the surrounding room. Mice were maintained on a 12:12-h light:dark cycle, at 72 ± 3°F ambient temperature, and 30-70% relative humidity.
Wild animals	*Provide details on animals observed in or captured in the field; report species and age where possible. Describe how animals were caught and transported and what happened to captive animals after the study (if killed, explain why and describe method; if released, say where and when) OR state that the study did not involve wild animals.*
Reporting on sex	sex of filarial worms were considered in study design because one of the goals of the study was to determine if there were differences between the sexes in the metabolites produced.
Field-collected samples	*For laboratory work with field-collected samples, describe all relevant parameters such as housing, maintenance, temperature, photoperiod and end-of-experiment protocol OR state that the study did not involve samples collected from the field.*
Ethics oversight	All animal experiments were conducted in accordance with the recommendations in the Guide for the Care and Use of Laboratory Animals of the National Institutes of Health. The NIAID Division of Intramural Research Animal Care and Use Committee approved all

animal work performed in this study. The NIH is fully accredited by AAALAC International, and all animal procedures were conducted under PHS Assurance number D16-00602 (A4149-01).

Note that full information on the approval of the study protocol must also be provided in the manuscript.

Plants

Seed stocks	*Report on the source of all seed stocks or other plant material used. If applicable, state the seed stock centre and catalogue number. If plant specimens were collected from the field, describe the collection location, date and sampling procedures.*
Novel plant genotypes	*Describe the methods by which all novel plant genotypes were produced. This includes those generated by transgenic approaches, gene editing, chemical/radiation-based mutagenesis and hybridization. For transgenic lines, describe the transformation method, the number of independent lines analyzed and the generation upon which experiments were performed. For gene-edited lines, describe the editor used, the endogenous sequence targeted for editing, the targeting guide RNA sequence (if applicable) and how the editor was applied.*
Authentication	Cell lines used were not authenticated in our lab
