# Supplementary material for: Secreted filarial acetylcholine analogs modulate parasite reproduction and host physiology
Source: Nat Commun. 2026 Aug 29;17:10336. doi: 10.1038/s41467-026-77090-2 (PMC13623875; doi:10.1038/s41467-026-77090-2)
Supplement: Supplementary file 2 [file 41467_2026_77090_MOESM13_ESM.pdf]

nature portfolio

Peer Review File

Secreted filarial acetylcholine analogs modulate parasite reproduction and host physiology

Corresponding Author: Professor Elodie Ghedin

This file contains all reviewer reports in order by version, followed by all author rebuttals in order by version.

Version 0:

Reviewer comments:

Reviewer #1

(Remarks to the Author)
MANUSCRIPT SUMMARY

This manuscript reports the discovery of novel male-enriched filarial acetylcholine analogs that are produced across multiple filarial species and may be derived from branched-chain amino acids. An untargeted metabolomics assay was used to identify novel sex specific compounds that may regulate nematode reproduction, including rapid suppression of microfilariae production. The authors further argue that these metabolic analogs selectively activate mammalian M2 muscarinic receptors, alter lymphatic vessel function, and disrupt endothelial adhesion pathways, suggesting a mechanistic link to lymphatic pathology. There is a clear integration of metabolite labeling, transcriptomics, novel compound synthesis, and receptor assays throughout the manuscript. Overall, it is a nice manuscript; however, some further description and characterization of the unknown metabolites needs be further explored along with some more method explanation and organization.

General Comments

Supp Figure 3. Three valerylcholine isomers are being proposed here and were semi-quantified with a PRM scan. Can a list be added of what a, b, c, d, and e correspond to for the chromatogram peaks so it is quicker to navigate the figure? Does GNPS, or using another program like Sirius 4, give other potential correlating mass spectra in other databases? The structures for fragments m/z 57 and 129 would also be helpful in Supp Fig. 3.

There should be a supplemental figure of the synthesized standards by themselves, rather than only spiked into the sample. How do these compounds elute using a different column or LC gradient? If you ran these out on different column, such as a chiral column or possibly using a GC, you should see if they still co-elute and would strengthen the data for the unknown identification. Deuterated internal standards or having 13C labelled stds to match would be ideal, however, synthesis may be cost and time prohibitive though at this point.

Is the R2 listed somewhere for the standard quantitative curves?

There are a lot of comparisons being performed here, with little in the way of explanation of statistical designs for each comparison or how false discovery rates were calculated.

The results can be a bit difficult to navigate, with comparisons made in the metabolomics section; some subheaders here would really help orient the reader along with what statistical test in each section is being performed. Also, a figure with pictures of the statistical designs, species, analyses, sample sizes, etc always helps to orient the reader.

Figure 1. It would be more intuitive if you could label what the metabolites of interest are on the volcano plots. The volcano plots in Fig 5 are much easier to read.

Line 118, several of the metabolites mentioned can be derived from the host microbiota (e.g. pipecolic acid mentioned 1 sentence later), is there a specific reason that folic acid in particular was linked to possible Wolbachia presence? Also, I don’t see where folic acid, pyridoxine, or pipecolic acid were validated against a standard. Somewhere the level of identification based on the Metabolomics Standards Initiative needs to be listed. A table would be a wonderful addition to

this manuscript, providing all pertinent information on the metabolites mentioned in the text. This way, the reader does not have to search through the supp table for it.

Following this thought, in supp table 3, why is Trp-Asn and Trp-Phe both listed with 2 different adducts? If this is a true hit you should ideally list the response of the same adduct for both or the grouped adduct signal. Do these also show up in neg mode? The authors should list which metabolites are significantly changed and found in both the neg and pos mode, this is an easy way to increase confidence in the identification. This would especially be useful if the novel acetylcholine compounds are showing multiple adducts and are present in both the pos and neg mode.

Specific comments

Line 57 can this PC abbreviation for phosphorylcholine be removed? PC is also used for pentanoylcholine, but phosphorylcholine does not appear to be used in the paper again anyway so there is not much use for its abbreviation.

Line 65 Typo

Line 100, change to “could not be identified”

Line 102, of the 2384 metabolites, are these supposed to be individual metabolites? How many were positive mode and how many were negative mode?

Line 129, where did the 5142 features come from? Line 102 lists 2384 metabolite peaks?

Line 527 typo

Line 617, was the labeled amino acid mixture used for any normalization or validation?

Reviewer #2

(Remarks to the Author)
This is an important and very interesting study on the potential function of secreted acetylcholine analogs in one of the agents of lymphatic filariasis, Brugia malayi, which employs a wide variety of orthogonal approaches. The authors have conducted an impressive feat of metabolic detective work to identify these analogs and their likely biosynthesis pathway. There are three main parts to the investigation: (1) identification of the ACh analogs and their synthesis pathway; (2) the effect of two licensed drugs of production of the ACh analogs; (3) the potential biological role of the ACh analogs in filarial reproduction; and (4) the effect of the ACh analogs on the mammalian host.

Regarding (1), the identification of the ACh analogs is the strongest part of the manuscript, but it is surprising that the authors do not place the putative metabolic pathways outlined in Fig. 3A in the context of their previous life-stage-specific metabolic models and RNA-Seq datasets (or B. malayi proteomic datasets from other labs). Are the activities of the enzymes catalysing each step of the process particularly high in adult males?

The main limitations of the study are around parts (2) and (3).

Regarding (2), the authors hypothesise that the observed increase in ACh analog levels following in vitro exposure to DOX could be due to depletion of Wolbachia symbionts that might otherwise compete for leucine/isoleucine. This is plausible considering the known dependency of Wolbachia on host amino acids but does not rule out a direct effect of DOX on filarial metabolism, including biosynthesis or degradation of the ACh analogs (especially considering the high dose of DOX applied). For instance, one in vivo study indicated that DOX inhibits acetylcholinesterase activity in the murine brain, which could increase ACh levels if filarial acetylcholinesterases are similarly affected (Ben-Azu et al. https://doi.org/10.1016/j.brainresbull.2018.02.007). Since DOX treatment was administered for 3 days only, a substantial depletion of Wolbachia density would not be expected (and could be easily checked using qPCR and/or imaging). Nevertheless, inhibition of Wolbachia metabolism could ensue prior to significant depletion, which is more difficult to assess, but could be estimated by RNA-Seq of Wolbachia. An obvious control would be to determine of the ACh analogs in aposymbiotic A. viteae also increased after DOX treatment. This isn’t simply of theoretical interest considering the mixed results of DOX therapy for filarial lymphedema (https://doi.org/10.1371/journal.ppat.0020092; https://pubmed.ncbi.nlm.nih.gov/39362213/) and uncertainty over the mode-of-action.

Regarding (3), the authors hypothesise that the ACh analogs regulate reproduction and generate some quite convincing data to support this, as shown in Fig. 5. However, the biological implications of a secreted male metabolite controlling female reproduction (in the case of B. malayi) are not fleshed-out. Since both sexes of worms are thought to remain together in worm nests, constant production of ACh analogs would interfere with Mf release, suggesting that in vivo there must be crosstalk and regulation of ACh analog production. This is clearly very difficult to demonstrate in vivo, or even in vitro. A concern is that the in vitro conditions are artefactual and production of the ACh analogs may be a function of the single-sex incubation of the worms, or the in vitro environment itself, and less about a natural aspect of male worm biology (especially as female A. viteae seem to be producing more of these molecules than the males). It would be informative to know if mixed-

sex cultures of B. malayi produce the ACh analogs at the expected level (i.e., proportional to the number of males present).

Regarding (4), the connection with the in vivo environment of the host is critical and both the LEC and mouse experiments are quite convincing (Fig. 7), although the latter would benefit from a statistical analysis of popliteal lymph node diameter for control v. treatments. In the main narrative and legend reporting the data in Fig. 6, the description of the switch from work on the human to the filarial muscarinic acetylcholine receptors is not entirely clear in the text. Although a focus on the B. malayi M2 receptor is justified from the human data, it would be useful to have some context on the number of putative muscarinic acetylcholine receptors in the parasite.

Minor points

For all figures displaying numeric data, the legend should state sample sizes and what the error bars represent. The figures in the supplement did not have intuitive file names or any figure numbers displayed in the uploaded documents.
The “Ethacrynic acid experiments and C13 labeled metabolites” methods section reads more like a shorthand lab protocol.
There are several typographical errors as follows:
Line 65 “effect son immunity”
Line 100 “the metabolites could not be by their MS2 library search.”
Legend for Fig. 1C “A. vitea worm ES”
Line 277 “However, non-detection is common for many CoA metabolites as can be covalently bound”
Lines 427 – 429. The following sentence is unclear: “Although binding of the isoforms to the human receptor was characterized by relatively similar molecular interactions as observed in B. malayi, isoforms bind differently from the human homolog suggesting higher specificity and selectivity towards B. malayi.”
Line 527 “anthelminticss”
Line 748 “Homo Sapiens”
Line 767 “(cell line obtained from Aashish Manglik at UCSF” – close bracket.
In Fig 7b, there is a citation to “Lake et al. Science” in the figure label.
Throughout the manuscript, the names of various molecules are capitalized unnecessarily; e.g., Isoleucine, Leucine.

Reviewer #3

(Remarks to the Author)
The Grote et al have identified and thoroughly investigated two secreted B. malayi metabolites of interest, supported by several approaches. The work is novel, with very few studies available in the literature performing metabolomics studies of parasitic nematodes. It is worth emphasizing in the discussion that these two metabolites are not only novel in filarial nematodes but they're novel overall in the literature, even in other eukaryote or microbiome studies. The manuscript is well-written, and the data is transparent and easily accessible with the detailed supplementary tables and code and disclosure as detailed in the Data Availability statement.
Most of the issues that need to be addressed prior to consideration for publication involve clarification of the experiments performed, provide more details related to the statistical methods used and significance, and some additional data interpretations could be included.
1. For Figure 2, it should be stated what the error bars represent, and either the individual points should be shown or at least the number of replicates should be stated directly in the caption.
2. For the doxycycline experiment (Figure 4), at 12.5µM for 3 days, how much was the worm overall fitness affected by the treatment? Were they still motile enough to conclude that what is observed isn't the result of worms dying? It would have been helpful to have another anthelmintic that doesn't target Wolbachia as a control to help confirm Wolbachia's importance.
3. For Figure 4, statistical differences are mentioned in the text but should also be shown on the graphs themselves. The caption should indicate the test that was used. Also, either the individual points should be shown or at least the number of replicates should be stated directly in the caption.
4. For the functional enrichment testing (GO and KEGG) were the results FDR-corrected? If so, the text could indicate FDR correction.
5. Were any of the enzymes from Fig 3A identified as being differentially regulated at a transcriptional level in the RNA-seq analysis? It would be informative to know their expression either way, to see whether deduced enzyme expression may or may not be influencing metabolite production or consumption.
6. For the egg laying and Mf secretion data in Figures 5B and 5C, the results are stated to be significant but no statistical tests or P values are provided in the text or on the figures. Individual points or sample counts should be provided for these as well.
7. "Genes were determined as differentially expressed using a threshold of $p < 0.05$ and a false-discovery rate (FDR) of 5%". This threshold is ok, but FDR is directly calculated from p values, so anything with FDR ≤ 0.05 will by definition have p ≤ 0.05, so it's not necessary to state both. No fold change cutoff is indicated, and that's ok but the volcano plots in Fig 5 show vertical dashed lines that would be expected to indicate a fold change threshold used. This could just be a single line at the fold change = 0 position.
8. Figure 6A, were any statistics performed to validate the increased fold changes? ANOVA could be used, or each of them could be compared to the M1 as a baseline. The methods indicate N=3, but this should be indicated in the caption.
9. Figure 7D, the exact P value should be shown and the statistical test used should be indicated in the caption.
10. Regarding the importance of 2-MBC and IVC to host-parasite interactions, since these are male-specific, perhaps these metabolites may be critical for tissue penetration as the males actively travel through the host to seek females which remain relatively stationary. This is speculative, but some more connection to sex-specific roles in terms of host-parasite interactions may strengthen this section of the discussion.

11. A. viteae females had much higher abundance of the two metabolites than the males (Fig 1C). Unlike B. malayi in the lymphatics, A. viteae females reside in the muscle tissue, so this finding may support the hypothesis that smooth muscle contraction is an important target for host-parasite interactions. Some level of interpretation about the A. viteae results should be added to the results/discussion.
Minor:
12. Line 527, "anthelminticss" misspelling.
13. Line 533 "raises intriguing the intriguing".

Reviewer #4

(Remarks to the Author)
I co-reviewed this manuscript with one of the reviewers who provided the listed reports. This is part of the Nature Communications initiative to facilitate training in peer review and to provide appropriate recognition for Early Career Researchers who co-review manuscripts.

Version 1:

Reviewer comments:

Reviewer #1

(Remarks to the Author)
The authors did an adequate job of addressing the comprehensive reviews.

Reviewer #2

(Remarks to the Author)
The manuscript has been thoroughly revised. While no new experiments have been performed (for instance, using A. viteae), there are good reasons for this. The only outstanding minor correction is to Fig. 7, where an odd label in panel A remains (see highlighted text in attachment).

Reviewer #3

(Remarks to the Author)
Overall, the authors have sufficiently addressed the concerns. I have only a few remaining minor additional suggestions:

- In the Figure 4 caption, "...standard deviation of mean error bars". This should either just be "standard deviation error bars" or "standard error of the mean error bars", depending which was used.

- When multiple test correction is described on lines 228, 230 and 743, describe the approach used for the correction (e.g. FDR correction)

- Reference 17 is listed as a bioRxiv, but has recently been published (PMID:42229419)

Reviewer #4

(Remarks to the Author)
I co-reviewed this manuscript with one of the reviewers who provided the listed reports. This is part of the Nature Communications initiative to facilitate training in peer review and to provide appropriate recognition for Early Career Researchers who co-review manuscripts.

Open Access This Peer Review File is licensed under a Creative Commons Attribution 4.0 International License, which permits use, sharing, adaptation, distribution and reproduction in any medium or format, as long as you give appropriate credit to the original author(s) and the source, provide a link to the Creative Commons license, and indicate if changes were made.
In cases where reviewers are anonymous, credit should be given to 'Anonymous Referee' and the source.
The images or other third party material in this Peer Review File are included in the article's Creative Commons license, unless indicated otherwise in a credit line to the material. If material is not included in the article's Creative Commons license and your intended use is not permitted by statutory regulation or exceeds the permitted use, you will need to obtain permission directly from the copyright holder.
To view a copy of this license, visit https://creativecommons.org/licenses/by/4.0/

Response to Reviewer Comments

Reviewer #1 (Remarks to the Author):

This manuscript reports the discovery of novel male-enriched filarial acetylcholine analogs that are produced across multiple filarial species and may be derived from branched-chain amino acids. An untargeted metabolomics assay was used to identify novel sex specific compounds that may regulate nematode reproduction, including rapid suppression of microfilariae production. The authors further argue that these metabolic analogs selectively activate mammalian M2 muscarinic receptors, alter lymphatic vessel function, and disrupt endothelial adhesion pathways, suggesting a mechanistic link to lymphatic pathology. There is a clear integration of metabolite labeling, transcriptomics, novel compound synthesis, and receptor assays throughout the manuscript. Overall, it is a nice manuscript; however, some further description and characterization of the unknown metabolites needs be further explored along with some more method explanation and organization.

General Comments

1. Supp Figure 3. Three valerylcholine isomers are being proposed here and were semi-quantified with a PRM scan. Can a list be added of what a, b, c, d, and e correspond to for the chromatogram peaks so it is quicker to navigate the figure?

Response: We thank the reviewer for their constructive review of our manuscript. We have improved **Supplementary Figure 3** by re-ordering it, adding in color-coding, and we changed the labels to facilitate interpretation of these key data, which confirm the novel metabolite structures. Specifically, the new figure makes it clearer that the spectra of the two endogenous peaks are fully consistent with the novel structures (2MBC and IVC).

2. Does GNPS, or using another program like Sirius 4, give other potential correlating mass spectra in other databases?

Response: We have updated this section of the manuscript, where we had performed a molecular clustering analysis using the GNPS platform, described in **Supplementary Figure 2**, and in the methods section "**Molecular Networking**". We originally did both traditional networking and molecular community networking of our experimental spectra from the ES samples with the goal of capturing any related structures. The novel acetylcholine analogs did not cluster with any other metabolites (as per the inset of Supp Fig 2 showing the 4 nodes for this cluster). The novel compounds, however, did automatically cluster with each other and with the MS2 spectra of their own natural isotopes.

Since we were also surprised that the analogs did not initially cluster with acetylcholine, we performed an additional molecular networking analysis using GNPS2 with all available acetylcholine reference spectra. In panel B of the updated Supplementary Figure 2, we show that our metabolites cluster with acetylcholine and do so via a direct edge, meaning that acetylcholine is the most similar molecule to our novel valerylcholines available in GNPS.

We have updated the section “Filarial metabolites are acetylcholine analogs” to include this result [Lines 200-204 of the highlighted version of the manuscript], and we now show acetylcholine clustering with the valerylcholines in **Supplementary Figure 2B**. We thank the reviewer again for this request, and we are excited to demonstrate how molecular networking could help with the identification of novel compounds.

3. The structures for fragments m/z 57 and 129 would also be helpful in Supp Fig. 3.

Response: We have made a major update to **Supplementary Figure 5**, annotating the MS2 fragment substructure assignments. We thank the reviewer for this recommendation, and we hope it helps the reader interpret the spectra of these novel structures. This new figure also helps highlight and clarify that there are actually several minor fragments which are unique to either 2-MBC or IVC (i.e., the 73 mz fragment is unique for 2-MBC, and the 85 and 87 mz is unique for IVC).

4. There should be a supplemental figure of the synthesized standards by themselves, rather than only spiked into the sample. How do these compounds elute using a different column or LC gradient? If you ran these out on different column, such as a chiral column or possibly using a GC, you should see if they still co-elute and would strengthen the data for the unknown identification. Deuterated internal standards or having 13C labelled stds to match would be ideal, however, synthesis may be cost and time prohibitive though at this point.

Response: We thank the reviewer for these questions. We have added extracted ion chromatograms of the 2 synthetic standards in **Supplementary Figure 4A**. We have also expanded and combined the data on isotope labeling with the substructure details into an updated **Supplementary Figure 5**, as mentioned above. These new figures include the MS2 spectra of the neat synthetic standards alone for this comparison **(Supplementary Figure 5 A and B)**. The spectra of the standards alone further confirm that the endogenous 2-MBC has an identical MS2 spectrum to the synthetic 2-MBC, and the same for IVC. Combined with the fragment annotation from the previous comment, this new figure clarifies the structural interpretation and confirmation of the novel compound spectra.

We have also analyzed these metabolites with a polar/HILIC chromatography, and these data are mentioned in-text and in **Supplementary Table 4**. However, we did not prioritize the polar analyses for most of the targeted work because the 2 isomers completely co-elute on the polar chromatography giving just 1 peak. Therefore, we used the reverse-phase separations for all of the targeted data in the manuscript. Other chromatography methods would be interesting, but the reverse-phase method fully separates the two novel compounds, which is why we used it for all subsequent analyses.

Regarding the isotope standards, it is unfortunately cost-prohibitive to synthesize these custom molecules with uniform carbon-13 at this time. However, a critical piece of data we report is the MS2 spectrum after biosynthesis with the carbon-13 precursors leucine and isoleucine

(**Supplementary Figure 5**). Importantly, the MS2 pattern for both of the novel biological compounds is identical to their respective synthesized standards, and to the unlabeled endogenous spectra, but with high resolution mass shifts of the fragment m/z, which matched our predictions based on the proposed structures and biological pathway. The section "*Biosynthetic pathways of filarial acetylcholine analogs use leucine and isoleucine as precursors*" discusses that even though the leucine/isoleucine precursors start with 6 labeled carbons, the biosynthesis pathway predicts loss of 1 carbon (the carboxylic acid carbon), and indeed, we only saw +5 labels, not +6. Further, the MS2 of the 13C-biosynthetic analogs confirms the location of the labeling on the structures (only on the aliphatic tail derived from leucines). Please see updated **Supplementary Figure 5,** which also shows that the substructure labeling is fully consistent with the biosynthesis pathway and predicted mass shifts. We thank the reviewer for suggesting this new figure, which helps demonstrate this critical validation.

With respect to the question of chiral chromatography/stereochemistry, the metabolic labeling experiments actually confirm the chemistry of the biosynthetic pathway. IVC does not have a stereocenter, but 2-MBC has one stereocenter. These labeling data show that no chemistry is happening on the 2-MBC stereocenter, meaning that it retains the same chirality as isoleucine (which is enantiopure). We have now stated the following at the end of section "*Biosynthetic pathways of filarial acetylcholine analogs use leucine and isoleucine as precursors*":

> Lines 292-294: "Further, because the pathway reactions do not alter the aliphatic tail, 2-MBC must retain the chirality of the isoleucine precursor, thereby confirming the 2-MBC stereocenter to be in the S-configuration (S-2-MBC)."

5. Is the R2 listed somewhere for the standard quantitative curves?

Response: Thank you for pointing out this missing information. We have now updated **Supplementary Figure 4** labels to include the r2 value for the linear regression.

6. There are a lot of comparisons being performed here, with little in the way of explanation of statistical designs for each comparison or how false discovery rates were calculated.

Response: We thank the reviewer for this helpful comment and agree that clearer presentation of our statistical designs and multiple-testing strategies improves the manuscript. We have made several changes:

- We made sure information on all statistical tests used was reported in the results section, as well as the corresponding figure legends, with more detailed descriptions in the Materials & Methods section.
- We added a **Supplementary Table 10** that now lists all the experiments, which comparisons were performed, and which statistical tests were performed.

We hope that these additions make the statistical design and comparisons more transparent and that they help orient the reader through the different datasets in the manuscript.

7. The results can be a bit difficult to navigate, with comparisons made in the metabolomics section; some subheaders here would really help orient the reader along with what statistical test in each section is being performed. Also, a figure with pictures of the statistical designs, species, analyses, sample sizes, etc always helps to orient the reader.

Response: We have made sure to add all information on statistical tests used to the results sections as well as any relevant figures, with more detailed descriptions in the Materials & Methods section. We have also added a new **Supplementary Table 10** which details all of these tests and conditions for each statistic reported in the manuscript.

8. Figure 1. It would be more intuitive if you could label what the metabolites of interest are on the volcano plots. The volcano plots in Fig 5 are much easier to read.

Response: We have now added red arrows next to the metabolite features of interest on the *B. malayi, B. pahangi*, and *A. viteae* volcano plots, and labeled the features of interest (188.16 m/z).

9. Line 118, several of the metabolites mentioned can be derived from the host microbiota (e.g. pipecolic acid mentioned 1 sentence later), is there a specific reason that folic acid in particular was linked to possible Wolbachia presence? Also, I don't see where folic acid, pyridoxine, or pipecolic acid were validated against a standard. Somewhere the level of identification based on the Metabolomics Standards Initiative needs to be listed. A table would be a wonderful addition to this manuscript, providing all pertinent information on the metabolites mentioned in the text. This way, the reader does not have to search through the supp table for it.

Response: We have attempted to clarify the text to simplify readability and added statistical values for any named metabolites mentioned throughout the text. We have also highlighted the row of the tables where the mentioned metabolites are listed. We thank the reviewer for highlighting the importance of the metabolomics standards initiative. We have now clarified in the methods section that all of the named metabolites are level 2 because of our spectral library approach to putative metabolite identification in global metabolomics. We have also clarified that all putative metabolites names have a minimum reverse-dot product of >900. We also manually inspected each metabolite mentioned in-text for chromatography quality, presence of isomers, and MS2 library match and score. As an example of this process, please see the below screenshot of the Folic Acid manual inspection:

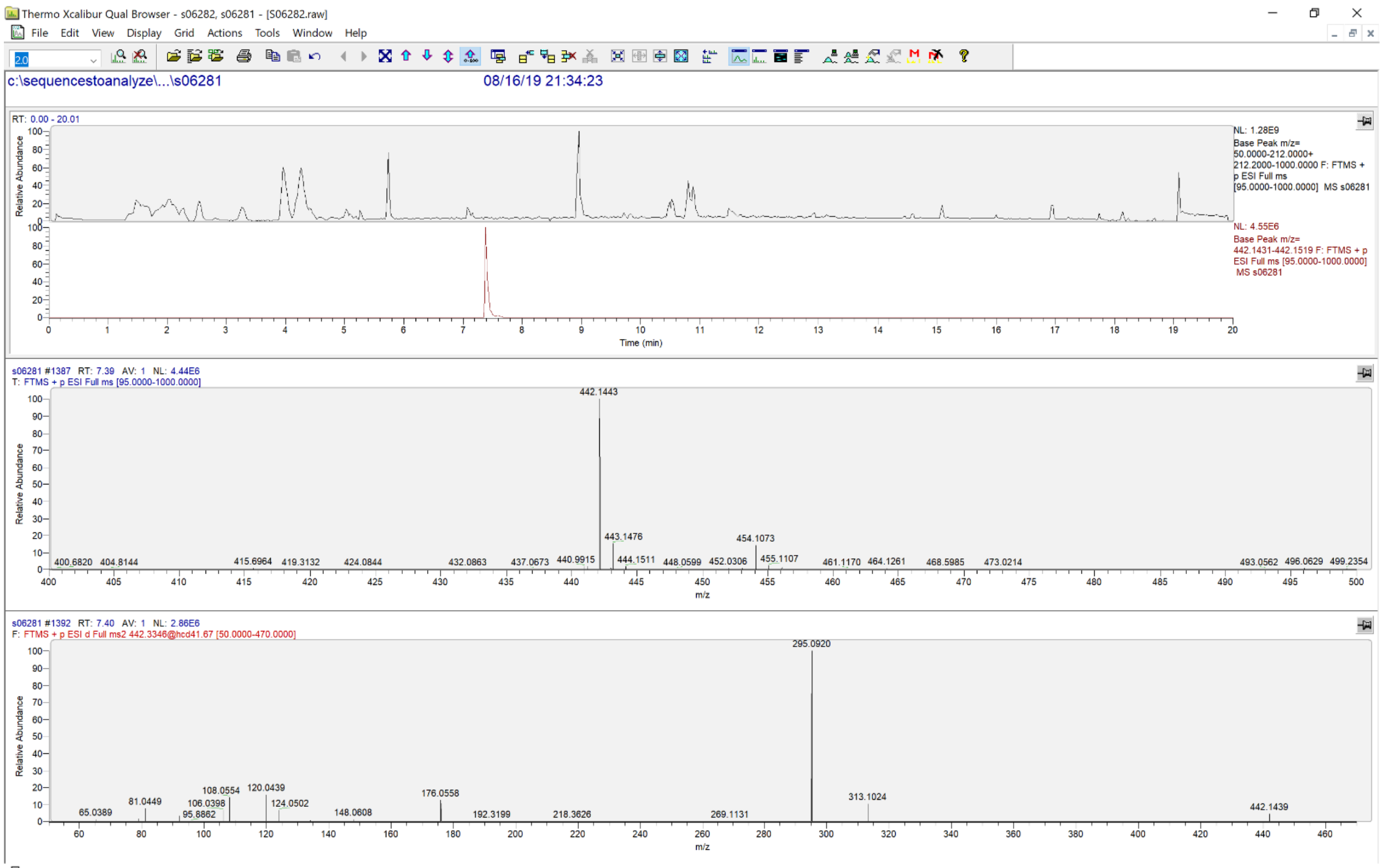
Thermo Xcalibur Qual Browser - s06282, s06281 - [S06282.raw]
File Edit View Display Grid Actions Tools Window Help
c:\sequencestoanalyze\...\s06281
08/16/19 21:34:23
RT: 0.00 - 20.01
NL: 1.28E9
Base Peak m/z= 50.0000-212.0000+ 212.2000-1000.0000 F: FTMS + p ESI Full ms [95.0000-1000.0000] MS s06281
NL: 4.55E6
Base Peak m/z= 442.1431-442.1519 F: FTMS + p ESI Full ms [95.0000-1000.0000] MS s06281
Relative Abundance
Time (min)
s06281 #1387 RT: 7.39 AV: 1 NL: 4.44E6
T: FTMS + p ESI Full ms [95.0000-1000.0000]
442.1443
443.1476
454.1073
m/z
s06281 #1392 RT: 7.40 AV: 1 NL: 2.86E6
F: FTMS + p ESI d Full ms2 442.3346@hcd41.67 [50.0000-470.0000]
295.0920
313.1024
176.0558
120.0439
108.0554
442.1439
m/z

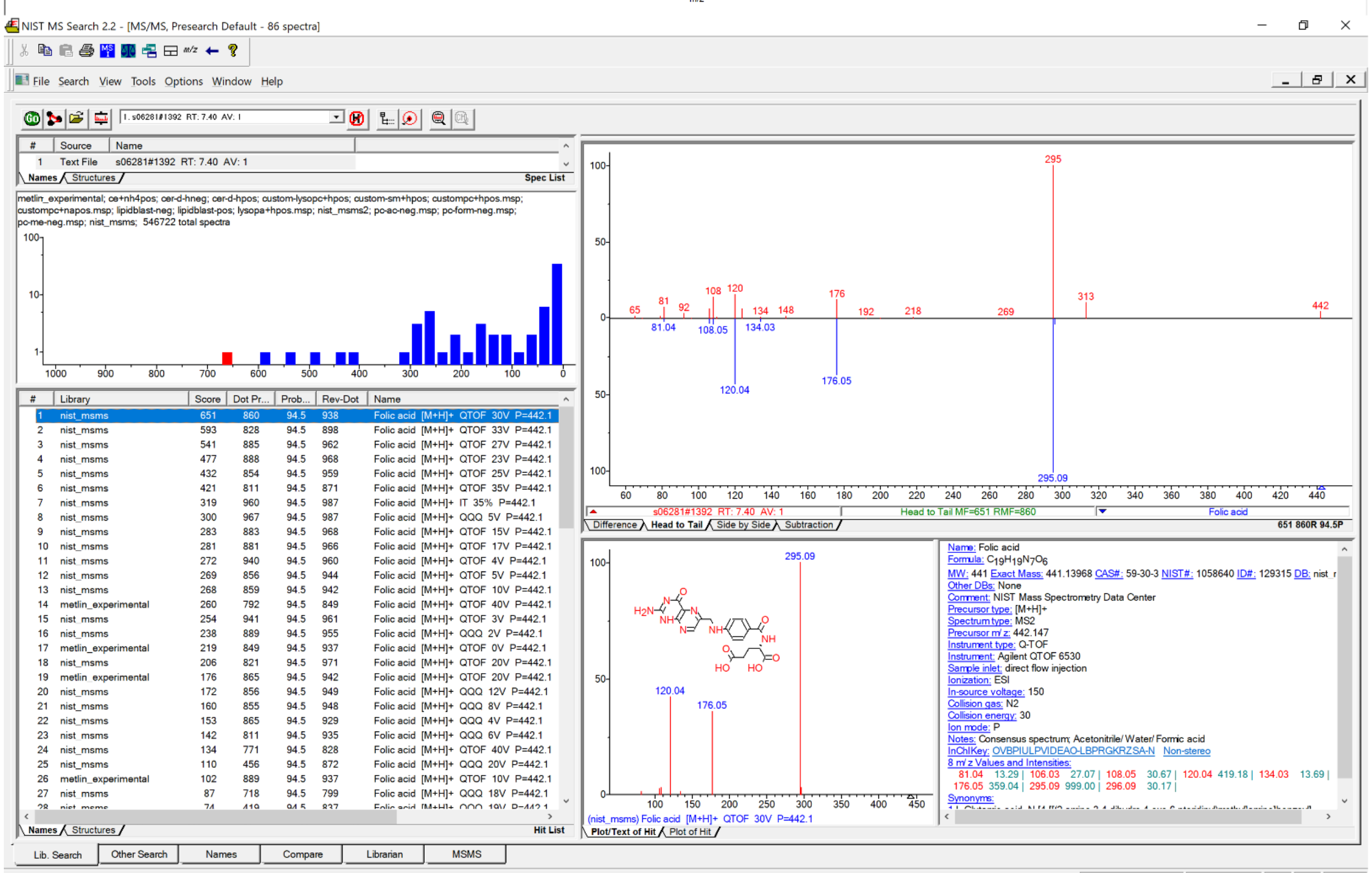
NIST MS Search 2.2 - [MS/MS, Presearch Default - 86 spectra]
File Search View Tools Options Window Help
1 Text File s06281#1392 RT: 7.40 AV: 1
Folic acid [M+H]+ QTOF 30V P=442.1
Head to Tail MF=651 RMF=860
651 860R 94.5P
Name: Folic acid
Formula: C19H19N7O6
MW: 441 Exact Mass: 441.13968 CAS#: 59-30-3 NIST#: 1058640 ID#: 129315
Comment: NIST Mass Spectrometry Data Center
Precursor type: [M+H]+
Spectrum type: MS2
Precursor m/z: 442.147
Instrument type: Q-TOF
Instrument: Agilent QTOF 6530
Sample inlet: direct flow injection
Ionization: ESI
In-source voltage: 150
Collision gas: N2
Collision energy: 30
Ion mode: P
Notes: Consensus spectrum; Acetonitrile/ Water/ Formic acid
InChIKey: OVBPIULPVIDEAO-LBPRGKRZSA-N
8 m/z Values and Intensities:
Lib. Search Other Search Names Compare Librarian MSMS

10. Following this thought, in supp table 3, why is Trp-Asn and Trp-Phe both listed with 2 different adducts? If this is a true hit you should ideally list the response of the same adduct for both or the grouped adduct signal. Do these also show up in neg mode? The authors should list which metabolites are significantly changed and found in both the neg and pos mode, this is an easy way to increase confidence in the identification. This would especially be useful if the novel acetylcholine compounds are showing multiple adducts and are present in both the pos and neg mode.

Response: We thank the reviewer for these questions, and we completely agree that evaluating other ion types and polarities is important for confirming compound identities. For the novel acetylcholine analogs, we carefully evaluated and discussed the ion type question in the 2nd paragraph [lines 192-194] of the section "*Filarial metabolites are acetylcholine analogs*". Because the two analogs are both quaternary amines, they have an intrinsic positive charge, and we exclusively detected them as [M]+ ions despite an extensive manual search for any co-eluting mzs (other adducts) in both positive and negative modes. This was one of our earliest hints at the molecular substructure of the unknown compounds, which led to the acetylcholine-like scaffold; requiring a quaternary amine moiety dramatically reduced the range of possible molecular configurations, leading to the choline moiety and subsequent aliphatic tail configurations.

For the two di-peptides this is a good example of our approach to global metabolite ID. Each of these names was independently identified, which in a sense increases the support, despite multiple rows for the same compound. These IDs are all level 2 annotations from high-scoring library matches (which is now clarified in the methods section). The data analysis pipeline keeps the best scoring hit for each unique inchicore, but exactly which spectrum (including polarity and ion type) has the best match could be any of the available spectra in the MS2 library, including different polarities, adducts, energies etc. Together, this means that two similar analytes may have different preferred ion types, because the putative naming comes from the best-matching library entry, and we then use that formula & ion type for the relative quantification of the best-matching feature. We hope that the detailed data in the supplementary tables enable readers to evaluate these types of questions.

11. Specific comments

(a) Line 57 can this PC abbreviation for phosphorylcholine be removed? PC is also used for pentanoylcholine, but phosphorylcholine does not appear to be used in the paper again anyway so there is not much use for its abbreviation.

Response: We agree that this is confusing, so we have removed the PC abbreviation for phosphorylcholine.

(b) Line 65 Typo

Response: Thank you for catching this, we have fixed the typo.

(c) Line 100, change to “could not be identified”

Response: Thank you for catching this, we have fixed the typo.

(d) Line 102, of the 2384 metabolites, are these supposed to be individual metabolites? How many were positive mode and how many were negative mode?

Response: These 2,384 untargeted features are defined as a specific mz at a specific retention time. There are 1,567 features in positive mode, and 817 features in negative mode. This information is shown in the “Polarity (z)” column of **Supplementary Table 2**, and the metabolomics methods section has been updated to cite the parameters for generation of the features. Metabolomics features are not necessarily unique molecules because of multiple possibilities for polarity, charge state, ion type, adducts, isotopes, and other factors, which can lead to one molecule generating multiple features.

(e) Line 129, where did the 5142 features come from? Line 102 lists 2384 metabolite peaks?

Response: The previous table of features (n=2,384) comes from the study on the different life-stages of the worms. **Supplementary Table 4** (n=5,142) comes from the study of the excreted/secreted metabolites from conditioned media of Brugia adult worms. The updated methods section describes the parameters for untargeted feature analysis. We hope these changes increase the clarity of the many metabolomics datasets, and we thank the reviewer.

(f) Line 527 typo

Response: (now line 531) Thank you for catching this, we have fixed the typo.

(g) Line 617, was the labeled amino acid mixture used for any normalization or validation?

Response: The amino acid mixture of internal standards was used exclusively for quality control assessment. We have added a section to the methods [Lines 720-727] on quality control of the metabolomics data, and we thank the reviewer for pointing out this important question.

Reviewer #2 (Remarks to the Author):

This is an important and very interesting study on the potential function of secreted acetylcholine analogs in one of the agents of lymphatic filariasis, Brugia malayi, which employs a wide variety of orthogonal approaches. The authors have conducted an impressive feat of metabolic detective work to identify these analogs and their likely biosynthesis pathway. There are three main parts to the investigation: (1) identification of the ACh analogs and their synthesis pathway; (2) the effect of two licensed drugs of production of the ACh analogs; (3) the potential biological role of the ACh analogs in filarial reproduction; and (4) the effect of the ACh analogs on the mammalian host.

Response: Thank you for this comprehensive description of the work!

1. Regarding (1), the identification of the ACh analogs is the strongest part of the manuscript, but it is surprising that the authors do not place the putative metabolic pathways outlined in Fig. 3A in the context of their previous life-stage-specific metabolic models and RNA-Seq datasets (or B. malayi proteomic datasets from other labs). Are the activities of the enzymes catalysing each step of the process particularly high in adult males?

Response: We agree that this was a missed opportunity. We were able to identify the choline acetyltransferase (ChAT) in the *B. malayi* genome as Bma-unc-17, an ortholog of *C. elegans* cha-1. Referencing our previously published life-stage-specific RNA-Seq data, we found that this enzyme is significantly upregulated in juvenile (30 DPI) and adult (120 DPI) males compared to females at the same developmental stages (logFC = 2.49 and 0.82 respectively, FDR < 0.05 for both). This male-biased expression pattern directly correlates with our observation that acetylcholine analogs are preferentially secreted by male worms, providing a molecular basis for the observed sex-specific metabolite production. We have now added this information to the Results [Lines 333-339].

2. The main limitations of the study are around parts (2) and (3).
   Regarding (2), the authors hypothesise that the observed increase in ACh analog levels following in vitro exposure to DOX could be due to depletion of Wolbachia symbionts that might otherwise compete for leucine/isoleucine. This is plausible considering the known dependency of Wolbachia on host amino acids but does not rule out a direct effect of DOX on filarial metabolism, including biosynthesis or degradation of the ACh analogs (especially considering the high dose of DOX applied). For instance, one in vivo study indicated that DOX inhibits acetylcholinesterase activity in the murine brain, which could increase ACh levels if filarial acetylcholinesterases are similarly affected (Ben-Azu et al. https://doi.org/10.1016/j.brainresbull.2018.02.007). Since DOX treatment was administered for 3 days only, a substantial depletion of Wolbachia density would not be expected (and could be easily checked using qPCR and/or imaging). Nevertheless,

inhibition of Wolbachia metabolism could ensue prior to significant depletion, which is more difficult to assess, but could be estimated by RNA-Seq of Wolbachia. An obvious control would be to determine of the ACh analogs in aposymbiotic A. viteae also increased after DOX treatment. This isn't simply of theoretical interest considering the mixed results of DOX therapy for filarial lymphedema (https://doi.org/10.1371/journal.ppat.0020092; https://pubmed.ncbi.nlm.nih.gov/39362213/) and uncertainty over the mode-of-action.

Response: Thank you for pointing us to that interesting murine study showing that doxycycline can inhibit acetylcholinesterase activity. We have added another piece of evidence that could point to the higher availability of leucine and isoleucine with doxycycline treatment: we provide an added panel in **Figure 4A** that shows the effect of doxycycline on leucine and isoleucine.

We have now added text referring to these data and a sentence as to the alternative interpretation on [Lines 324-331]: "We also observed that doxycycline treatment led to an increase in both leucine and isoleucine, the precursors of the valerylcholines [Figure 4A, right panel]. These results suggest *Wolbachia*, which is impacted by the antibiotic treatment, may be competing with the worms for the amino acids or nutrients, including leucine and isoleucine, leading to increased availability for other downstream metabolite products such as the valerylcholines. An alternative interpretation is that doxycycline is inhibiting acetylcholinesterase activity, as seen in other studies {Ben-Azu et al}, which would lead to an increase in acetylcholine analogs in the treated worms because of less degradation."

We agree with the reviewer that testing Ach analogs in *A. viteae* would be an interesting experiment. Unfortunately, *A. viteae* worms are no longer available to us from any known supplier or any of our prior suppliers. Additionally, to our knowledge there isn't any RNA-seq data available for *Wolbachia* after a short Doxycycline treatment. These are extremely interesting questions which we hope to pursue in subsequent work.

3. Regarding (3), the authors hypothesise that the ACh analogs regulate reproduction and generate some quite convincing data to support this, as shown in Fig. 5. However, the biological implications of a secreted male metabolite controlling female reproduction (in the case of B. malayi) are not fleshed-out. Since both sexes of worms are thought to remain together in worm nests, constant production of ACh analogs would interfere with Mf release, suggesting that in vivo there must be crosstalk and regulation of ACh analog production. This is clearly very difficult to demonstrate in vivo, or even in vitro. A concern is that the in vitro conditions are artefactual and production of the ACh analogs may be a function of the single-sex incubation of the worms, or the in vitro environment itself, and less about a natural aspect of male worm biology (especially as female A. viteae seem to be producing more of these molecules than the males). It would be informative to know if mixed-sex cultures of B. malayi produce the ACh analogs at the expected level (i.e., proportional to the number of males present).

Response: We agree that the *in vitro* conditions have a risk of artefactually highlighting a single aspect of the regulation of the ACh analog production by the males. Further, we agree that it is biologically likely that there is some crosstalk *in vivo* between the male and female worms that we are not capturing with this experimental model. However, it is also unclear what happens *in vivo*: while *B. malayi* males coil around the posterior end of the females for mating, post-mating they are no longer connected physically. They are likely still in close proximity but lymph flow is constant and so it is unclear how much of what the male produces would impact the female when not in close association. Therefore, this question is extremely interesting, and one we are pursuing in the future with an experimental plan that involves a large number of worms. We will perform various permutations where we co-culture males and females at different ratios. This will involve making sure the worms are also developmentally phased as much as possible, which will require coordination with the laboratory, FR3, which provides the worms to us (and to many others in the US).

4. Regarding (4), the connection with the in vivo environment of the host is critical and both the LEC and mouse experiments are quite convincing (Fig. 7), although the latter would benefit from a statistical analysis of popliteal lymph node diameter for control v. treatments.

Response: We thank the reviewer for their comment. Regarding **Figure 7**, a statistical analysis of popliteal lymph node diameter for control v. treatments would not be informative as there wouldn't be any changes in lymph node size or architecture yet at 2 hours post-inoculation. The microscopic images confirm vessel patency and structural integrity across treatment conditions and represent accurate observations; however, without standardization of imaging location and vessel selection across animals, we do not feel it is appropriate to derive quantitative diameter measurements from the vessels. Meaningful morphometric analysis would require a dedicated imaging protocol and a substantially larger cohort. We therefore present the images as qualitative confirmation of normal vessel architecture and rely on lymph node viral accumulation as our quantitative functional readout.

5. In the main narrative and legend reporting the data in Fig. 6, the description of the switch from work on the human to the filarial muscarinic acetylcholine receptors is not entirely clear in the text. Although a focus on the B. malayi M2 receptor is justified from the human data, it would be useful to have some context on the number of putative muscarinic acetylcholine receptors in the parasite.

Response: Thank you for pointing out this gap in narrative. We have now added a sentence describing what is known about GPCRs in *B. malayi* [Lines 458-460]. We have also renamed the *B. malayi* M2 to GAR-2 as this is the potential homolog of the mammalian M2.

6. Minor points

(a) For all figures displaying numeric data, the legend should state sample sizes and what the error bars represent. The figures in the supplement did not have intuitive file names or any figure numbers displayed in the uploaded documents.

Response: Thank you for pointing out these omissions. We have added this information where relevant.

(b) The "Ethacrynic acid experiments and C13 labeled metabolites" methods section reads more like a shorthand lab protocol.

Response: We have expanded the methods section for this experiment and apologize for the abbreviated description.

(c) There are several typographical errors as follows:
Line 65 "effect son immunity"

Response: Thank you for catching this, we have fixed the typo.

(d) Line 100 "the metabolites could not be by their MS2 library search."

Response: Thank you for catching this, we have fixed the typo.

(e) Legend for Fig. 1C "A. vitea worm ES"

Response: Thank you for catching this, we have fixed the typo.

(f) Line 277 "However, non-detection is common for many CoA metabolites as can be covalently bound"

Response: Thank you for catching this, we have fixed the typo.

(g) Lines 427 – 429. The following sentence is unclear: "Although binding of the isoforms to the human receptor was characterized by relatively similar molecular interactions as observed in B. malayi, isoforms bind differently from the human homolog suggesting higher specificity and selectivity towards B. malayi."

Response: We have now rewritten this sentence for clarity. [Lines 480-483]

(h) Line 527 "anthelminticss"

Response: Thank you for catching this, we have fixed the typo.

(i) Line 748 "Homo Sapiens"

Response: Thank you for catching this, we have fixed the typo.

(j) Line 767 "(cell line obtained from Aashish Manglik at UCSF" – close bracket.

Response: Thank you for catching this, we have fixed the typo.

(k) In Fig 7b, there is a citation to "Lake et al. Science" in the figure label.

Response: We were unable to find this reference in the figure label and hope it has now disappeared.

(l) Throughout the manuscript, the names of various molecules are capitalized unnecessarily; e.g., Isoleucine, Leucine.

Response: Thank you for pointing this out- we have made sure all molecule names are not capitalized.

Reviewer #3 (Remarks to the Author):

The Grote et al have identified and thoroughly investigated two secreted B. malayi metabolites of interest, supported by several approaches. The work is novel, with very few studies available in the literature performing metabolomics studies of parasitic nematodes. It is worth emphasizing in the discussion that these two metabolites are not only novel in filarial nematodes but they're novel overall in the literature, even in other eukaryote or microbiome studies. The manuscript is well-written, and the data is transparent and easily accessible with the detailed supplementary tables and code and disclosure as detailed in the Data Availability statement. Most of the issues that need to be addressed prior to consideration for publication involve clarification of the experiments performed, provide more details related to the statistical methods used and significance, and some additional data interpretations could be included.

1. For Figure 2, it should be stated what the error bars represent, and either the individual points should be shown or at least the number of replicates should be stated directly in the caption.

Response: We thank the reviewer for this positive assessment of the work, as well as for highlighting the novelty of these analog structures across biology more widely. We have added to the legend of figure 2 the number of worms lysed in each of the 3 biological replicates. We have also added information of what the error bar represents (mean standard deviation error bars)

2. For the doxycycline experiment (Figure 4), at 12.5µM for 3 days, how much was the worm overall fitness affected by the treatment? Were they still motile enough to conclude that what is

observed isn't the result of worms dying? It would have been helpful to have another anthelmintic that doesn't target Wolbachia as a control to help confirm Wolbachia's importance.

Response: We used a short exposure to doxycycline at 12.5 µM for 3 days to perturb *Wolbachia* while avoiding acute killing of adult worms. This dose/time course is supported by previous studies where doxycycline was used *in vitro* against Brugia for 3 or 6 days. We have added information to that effect [Lines 319-320]. Importantly, after 3 and even 6 days, doxycycline-treated *B. malayi* showed no significant reduction in MTT/NAD(P)H activity assay (the assay to test worm viability) compared with DMSO-treated controls (https://doi.org/10.1371/journal.pone.0153812, Figure 5C and D from cited manuscript). This is consistent with the established anti-*Wolbachia* mechanism of doxycycline in filarial worms, in which *Wolbachia* clearance leads to delayed effects on reproduction (days 6 and 7 post-treatment in vitro) (DOI: 10.1371/journal.ppat.1002351). Therefore, worms in our 3-day treatment were sufficiently viable/motile, and the observed phenotype reflects *Wolbachia* perturbation rather than nonspecific worm death.

3. For Figure 4, statistical differences are mentioned in the text but should also be shown on the graphs themselves. The caption should indicate the test that was used. Also, either the individual points should be shown or at least the number of replicates should be stated directly in the caption.

Response: We have added to the text the statistical test used as well as the number of replicates.

4. For the functional enrichment testing (GO and KEGG) were the results FDR-corrected? If so, the text could indicate FDR correction.

Response: Thank you for catching this. We had intended to report FDR-corrected results, not p-values. We have rectified this in the manuscript.

5. Were any of the enzymes from Fig 3A identified as being differentially regulated at a transcriptional level in the RNA-seq analysis? It would be informative to know their expression either way, to see whether deduced enzyme expression may or may not be influencing metabolite production or consumption.

Response: We agree that this was a missed opportunity. We were able to identify the choline acetyltransferase (ChAT) in the *B. malayi* genome as Bma-unc-17, an ortholog of *C. elegans* cha-1. Referencing our previously published life-stage-specific RNA-Seq data, we found that this enzyme is significantly upregulated in juvenile (30 DPI) and adult (120 DPI) males compared to females at the same developmental stages (logFC = 2.49 and 0.82 respectively, FDR < 0.05 for both). This male-biased expression pattern directly correlates with our observation that acetylcholine analogs are preferentially secreted by male worms, providing a

molecular basis for the observed sex-specific metabolite production. We have added this to the manuscript. [Lines 333-339]

6. For the egg laying and Mf secretion data in Figures 5B and 5C, the results are stated to be significant but no statistical tests or P values are provided in the text or on the figures. Individual points or sample counts should be provided for these as well.

Response: We apologize for this omission and have now added the statistical tests, p-values, and sample counts in the caption of the figure.

7. "Genes were determined as differentially expressed using a threshold of p <0.05 and a false-discovery rate (FDR) of 5%". This threshold is ok, but FDR is directly calculated from p values, so anything with FDR ≤ 0.05 will by definition have p ≤ 0.05, so it's not necessary to state both. No fold change cutoff is indicated, and that's ok but the volcano plots in Fig 5 show vertical dashed lines that would be expected to indicate a fold change threshold used. This could just be a single line at the fold change = 0 position.

Response: We have clarified the use of FDR ≤ 0.05 as our threshold and removed the mention of p-value. And for clarity, we have added what the dashed lines in Figure 5 represent.

8. Figure 6A, were any statistics performed to validate the increased fold changes? ANOVA could be used, or each of them could be compared to the M1 as a baseline. The methods indicate N=3, but this should be indicated in the caption.

Response: We apologize for this omission and have now updated the figure and clarified the tests done and comparisons.

9. Figure 7D, the exact P value should be shown and the statistical test used should be indicated in the caption.

Response: We apologize for not providing that information. We have now added in the figure caption the test used (a Mann-Whitney U test) with p-value.

10. Regarding the importance of 2-MBC and IVC to host-parasite interactions, since these are male-specific, perhaps these metabolites may be critical for tissue penetration as the males actively travel through the host to seek females which remain relatively stationary. This is speculative, but some more connection to sex-specific roles in terms of host-parasite interactions may strengthen this section of the discussion.

Response: We agree that the discussion could benefit from further discussion of the potential sex-specific roles of the parasite and how these metabolites may fit in. We have added a section to the discussion [Lines 568-582].

11. A. viteae females had much higher abundance of the two metabolites than the males (Fig 1C). Unlike B. malayi in the lymphatics, A. viteae females reside in the muscle tissue, so this finding may support the hypothesis that smooth muscle contraction is an important target for host-parasite interactions. Some level of interpretation about the A. viteae results should be added to the results/discussion.

Response: Thank you for providing this very interesting information. We have now added a statement to that effect in the Discussion. [Lines 568-582]

Minor:
12. Line 527, "anthelminticss" misspelling.

Response: Thank you for catching this, we have fixed the typo.

13. Line 533 "raises intriguing the intriguing".

Response: Thank you for catching this, we have fixed the typo.

Reviewer #4 (Remarks to the Author):

I co-reviewed this manuscript with one of the reviewers who provided the listed reports. This is part of the Nature Communications initiative to facilitate training in peer review and to provide appropriate recognition for Early Career Researchers who co-review manuscripts.

Response: We thank the reviewer for their input in the review process.

We thank the reviewers for taking the time to re-review our manuscript. We have addressed each comment below.

Reviewer #2 (Remarks to the Author):
The manuscript has been thoroughly revised. While no new experiments have been performed (for instance, using A. viteae), there are good reasons for this. The only outstanding minor correction is to Fig. 7, where an odd label in panel A remains (see highlighted text in attachment).

RESPONSE: Thank you for highlighting the odd label in panel A of Figure 7. It turns out that this is the annotation that is in the ShinyGO database. We have gone through the paper the label references and have corrected the label to what the database should have listed.

Reviewer #3 (Remarks to the Author):

Overall, the authors have sufficiently addressed the concerns. I have only a few remaining minor additional suggestions:

- In the Figure 4 caption, "...standard deviation of mean error bars". This should either just be "standard deviation error bars" or "standard error of the mean error bars", depending which was used.

RESPONSE: Thank you for highlighting the inconsistency. We have now rewritten the statement and it now reads: "shown as mean MS intensity +/- standard deviation"

- When multiple test correction is described on lines 228, 230 and 743, describe the approach used for the correction (e.g. FDR correction)

RESPONSE: We have now added that information in the relevant places.

- Reference 17 is listed as a bioRxiv, but has recently been published (PMID:42229419)

RESPONSE: Thank you very much. We have now updated the reference.
