# Supplementary material for: Secreted filarial acetylcholine analogs modulate parasite reproduction and host physiology
Source: Nat Commun. 2026 Aug 29;17:10336. doi: 10.1038/s41467-026-77090-2 (PMC13623875; doi:10.1038/s41467-026-77090-2)
Supplement: Supplementary file 3 [file 41467_2026_77090_MOESM1_ESM.pdf]

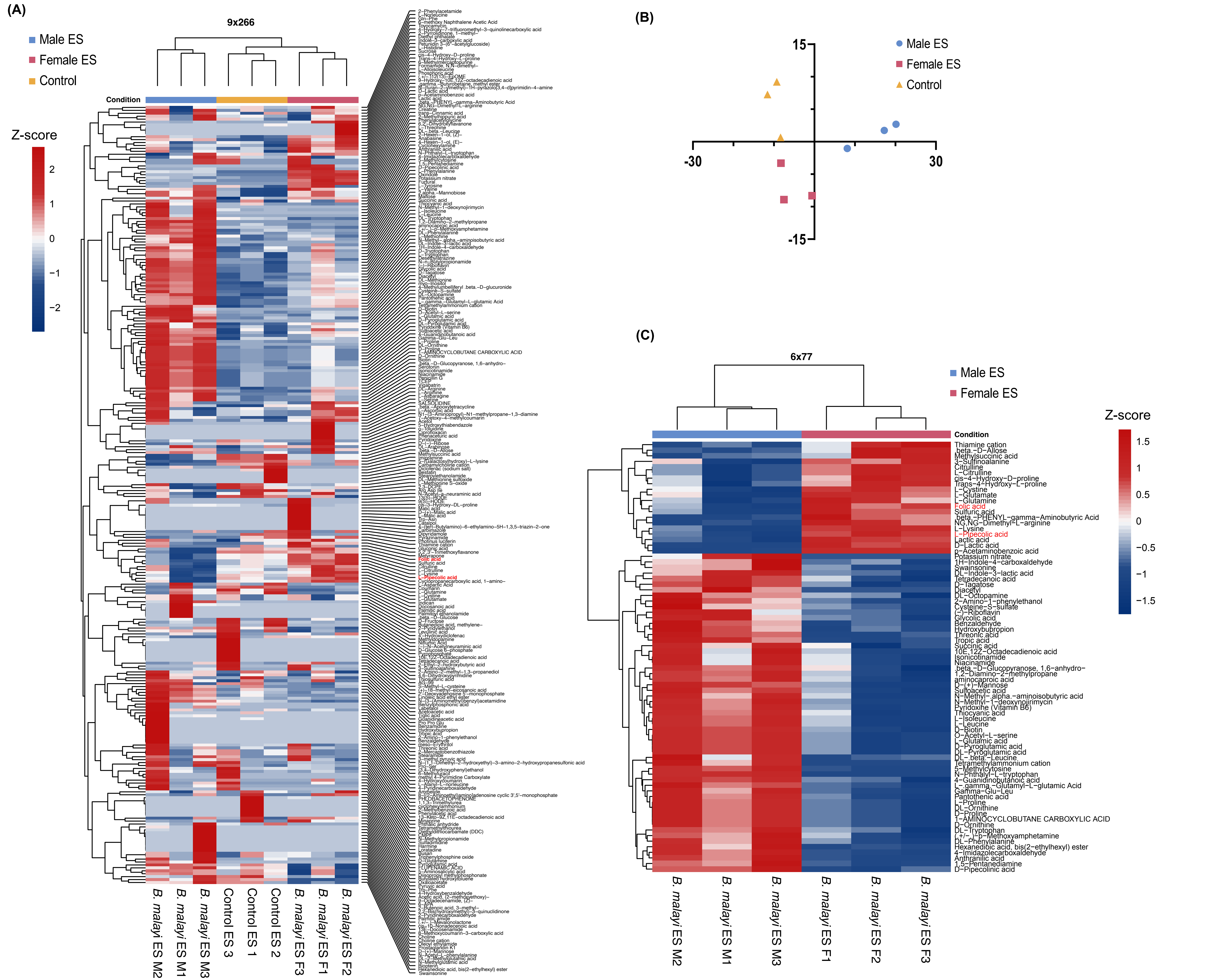
(A)
9x266
Male ES
Female ES
Control
Condition
Z-score
2
1
0
−1
−2
B. malayi ES M2
B. malayi ES M1
B. malayi ES M3
Control ES 3
Control ES 1
Control ES 2
B. malayi ES F3
B. malayi ES F1
B. malayi ES F2
(B)
15
-15
-30
30
Male ES
Female ES
Control
(C)
6x77
Male ES
Female ES
Condition
Z-score
1.5
1
0.5
0
−0.5
−1
−1.5
Thiamine cation
.beta.-D-Allose
Methylsuccinic acid
3-Sulfinoalanine
Citrulline
L-Citrulline
cis-4-Hydroxy-D-proline
Trans-4-Hydroxy-L-proline
L-Cystine
L-Glutamate
L-Glutamine
Folic acid
Sulfuric acid
.beta.-PHENYL-gamma-Aminobutyric Acid
NG,NG-Dimethyl-L-arginine
L-Lysine
L-Pipecolic acid
Lactic acid
D-Lactic acid
p-Acetaminobenzoic acid
Potassium nitrate
1H-Indole-4-carboxaldehyde
Swainsonine
DL-Indole-3-lactic acid
Tetradecanoic acid
D-Tagatose
Diacetyl
DL-Octopamine
2-Amino-1-phenylethanol
Cysteine-S-sulfate
(-)-Riboflavin
Glycolic acid
Benzaldehyde
Hydroxybupropion
Threonic acid
Tropic acid
Succinic acid
10E,12Z-Octadecadienoic acid
Isonicotinamide
Niacinamide
.beta.-D-Glucopyranose, 1,6-anhydro-
1,2-Diamino-2-methylpropane
aminocaproic acid
D-(+)-Mannose
Sulfoacetic acid
N-Methyl-.alpha.-aminoisobutyric acid
N-Methyl-1-deoxynojirimycin
Pyridoxine (Vitamin B6)
Thiocyanic acid
L-Isoleucine
L-Leucine
D-Biotin
O-Acetyl-L-serine
L-Glutamic acid
D-Pyroglutamic acid
DL-Pyroglutamic acid
DL-.beta.-Leucine
Tetramethylammonium cation
5-Methylcytosine
N-Phthalyl-L-tryptophan
4-Guanidinobutanoic acid
L-.gamma.-Glutamyl-L-glutamic Acid
Gamma-Glu-Leu
Pantothenic acid
L-Proline
DL-Ornithine
D-Proline
1-AMINOCYCLOBUTANE CARBOXYLIC ACID
D-Ornithine
DL-Tryptophan
(+/-)-p-Methoxyamphetamine
DL-Phenylalanine
Hexanedioic acid, bis(2-ethylhexyl) ester
4-Imidazolecarboxaldehyde
Anthranilic acid
1,5-Pentanediamine
D-Pipecolinic acid
B. malayi ES M2
B. malayi ES M1
B. malayi ES M3
B. malayi ES F1
B. malayi ES F2
B. malayi ES F3

Supplementary Figure 1: Conditioned media from male and female *B. malayi* worms have a distinct molecular composition. (A) Unsupervised clustering analysis of putatively identified metabolites in spent media of cultured worms, 2 worms per 2 ml of media, experiment done in triplicate (n=3). Control represents media without worms. Samples were clustered with the Complete method and Euclidian distance function. **(B)** Principal component analysis of identified metabolites by sample groups. **(C)** Hierarchical clustering of statistically different metabolites between Male ES and Female ES. A two-tailed t-test, raw p-values, was applied. Color legend indicates z-score. Source data are provided as a Source Data file.

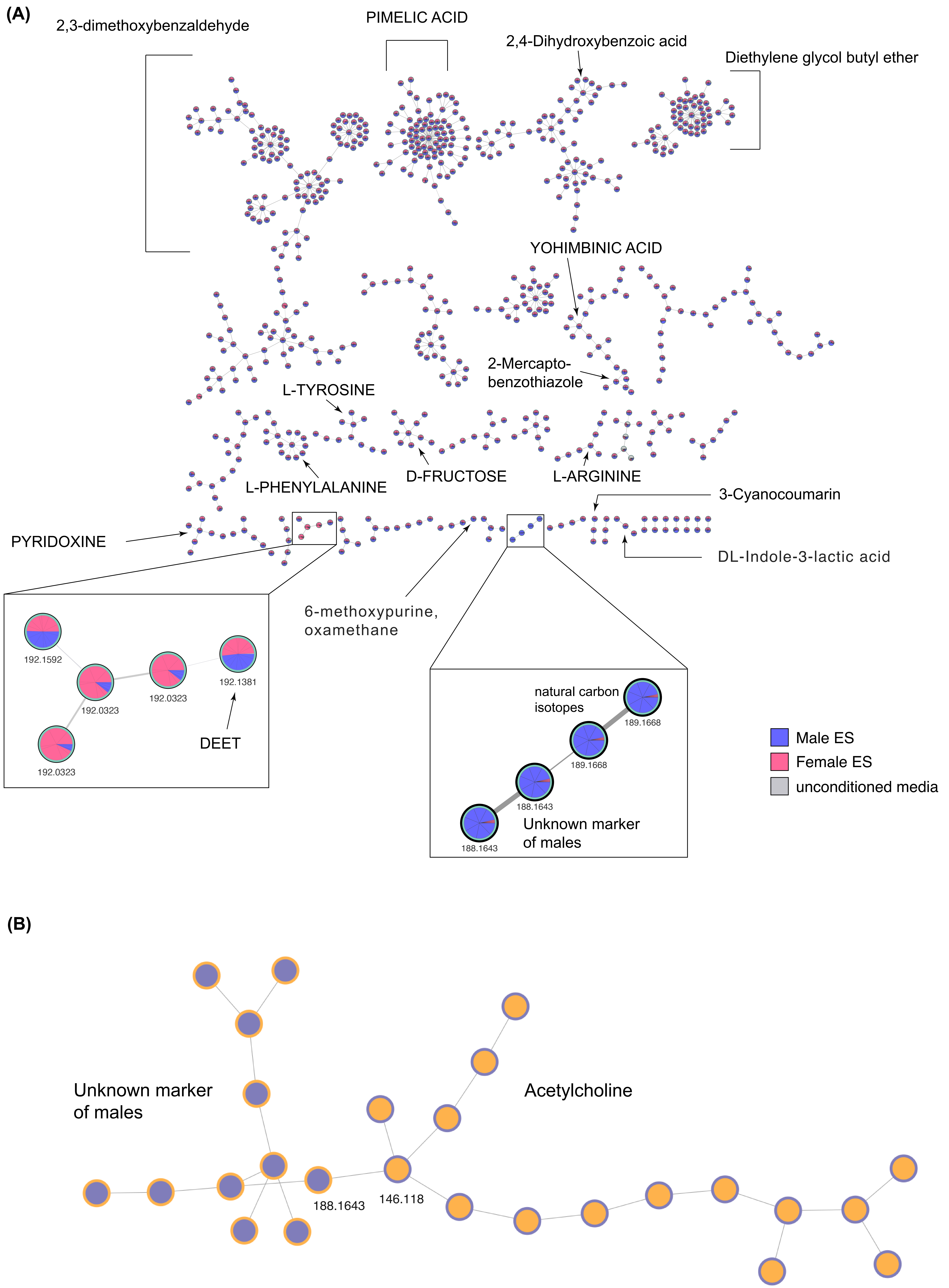
(A)
2,3-dimethoxybenzaldehyde
PIMELIC ACID
2,4-Dihydroxybenzoic acid
Diethylene glycol butyl ether
YOHIMBINIC ACID
2-Mercapto-
benzothiazole
L-TYROSINE
L-PHENYLALANINE
D-FRUCTOSE
L-ARGININE
3-Cyanocoumarin
PYRIDOXINE
DL-Indole-3-lactic acid
6-methoxypurine,
oxamethane
192.1592
192.0323
192.0323
192.1381
192.0323
DEET
natural carbon
isotopes
189.1668
189.1668
188.1643
188.1643
Unknown marker
of males
Male ES
Female ES
unconditioned media
(B)
Unknown marker
of males
Acetylcholine
188.1643
146.118

Supplementary Figure 2: Molecular Network Analysis using GNPS. (A) Molecular network of *B. pahangi* ES metabolites based on MS/MS spectral similarity. Each node is a representative metabolite feature (m/z, rt, MS2) after deduplication of replicate experimental spectra (0.005 m/z precursor, 10sec). Edges connect nodes with non-zero cosine scores, which quantify similarity between their tandem MS2 spectra. Edge thickness is proportional to the cosine score, ranging from 0 (lowest similarity) to 6 (highest similarity, indicating very similar or nearly identical spectra). Pie-chart node colors indicate relative signal contributions (MS1 intensity) from male ES (blue), female ES (magenta), and unconditioned media (grey). Metabolite name labels come from MS2 library matching. **(B)** Molecular network of connections between ES metabolites and GNPS reference spectra. Each node is a representative metabolite feature (m/z, rt, MS2) after deduplication of replicate experimental spectra (0.005 m/z precursor, 10sec). Edges connect nodes with non-zero cosine scores that quantify the similarity between their tandem MS2 spectra. The abundance information is not included and the network shows connectivity based on MS/MS similarity only. Coloring represents where the MS2 spectra come from, where purple represents experimental spectra, and orange represents acetylcholine library spectra.

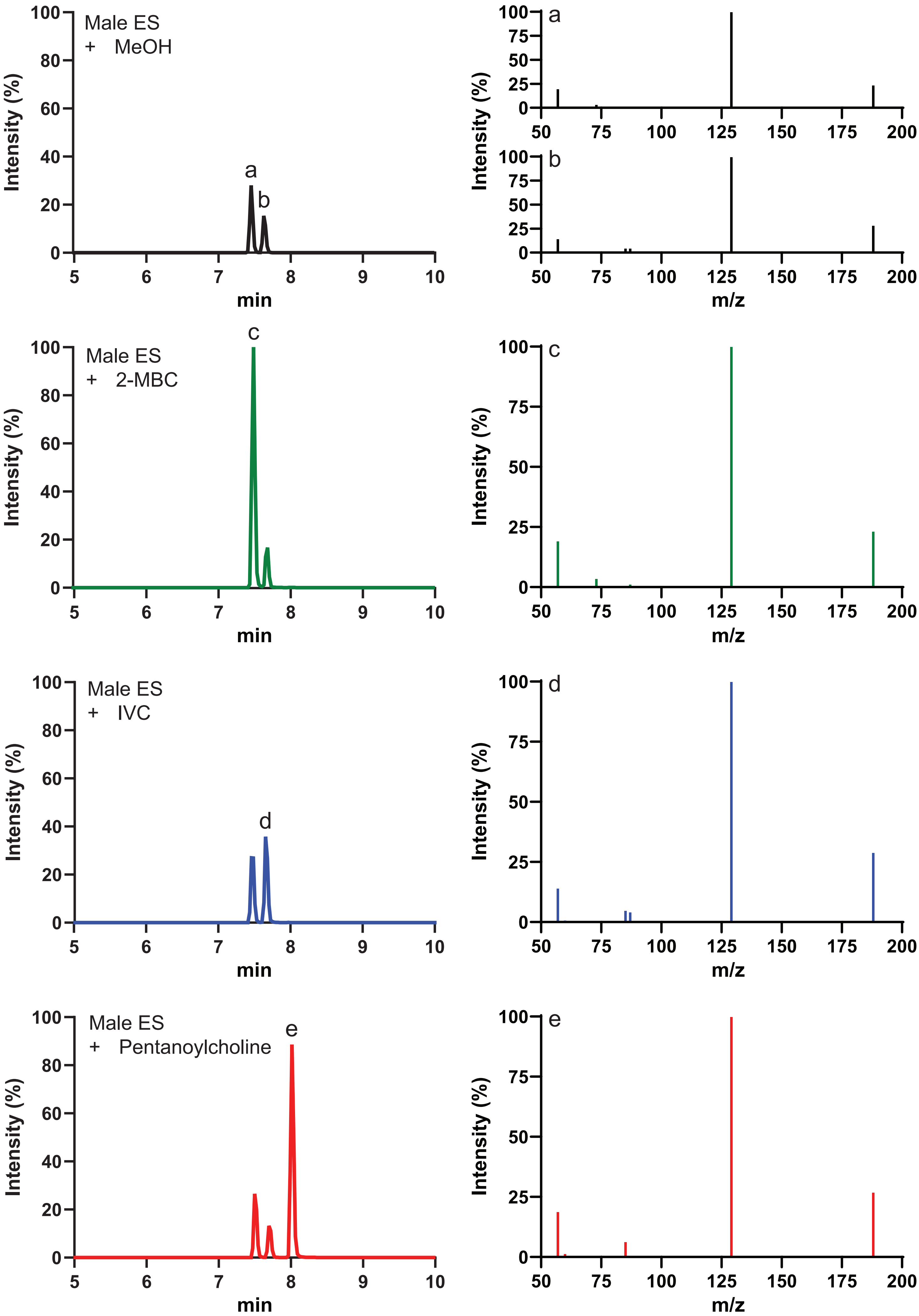

Male ES
+ MeOH
a
b
Intensity (%)
min
Male ES
+ 2-MBC
c
Male ES
+ IVC
d
Male ES
+ Pentanoylcholine
e
m/z

Supplementary Figure 3: Validation of synthesized compounds. Spike-in experiments of synthesized compounds into authentic *B. pahangi* male conditioned media (male ES). Left side plots are extracted ion chromatograms (MS1, 188.1645 m/z, 15ppm tolerance). Right side plots are MS2 spectra corresponding to the peaks (left) with same letter label. Peaks a and b show the endogenous metabolites from the conditioned media. Spike-in of synthetic 2-MBC increases height of first peak (peak c) as compared to the methanol control (peak a), indicating co-elution with the endogenous metabolite. Comparing the MS2 of the co-eluting peak (spectrum c) with that of the endogenous alone (spectrum a) resulted in an identical pattern, with no change in relative fragment abundance, even for the minor fragments. Similarly, spike-in of synthetic IVC increases height of the second peak (peak d), indicating co-elution with the second endogenous peak (peak b) and no change in the MS2 pattern compared to the peak without spike-in (spectrum d vs spectrum b). Synthetic pentanoylcholine (peak e) resolves as a third isomeric peak while showing the same major MS2 fragments as the endogenous peaks, indicating a close but not identical match. 2-MBC: 2-Methylbutyrylcholine; IVC: Isovalerylcholine; MeOH: methanol.

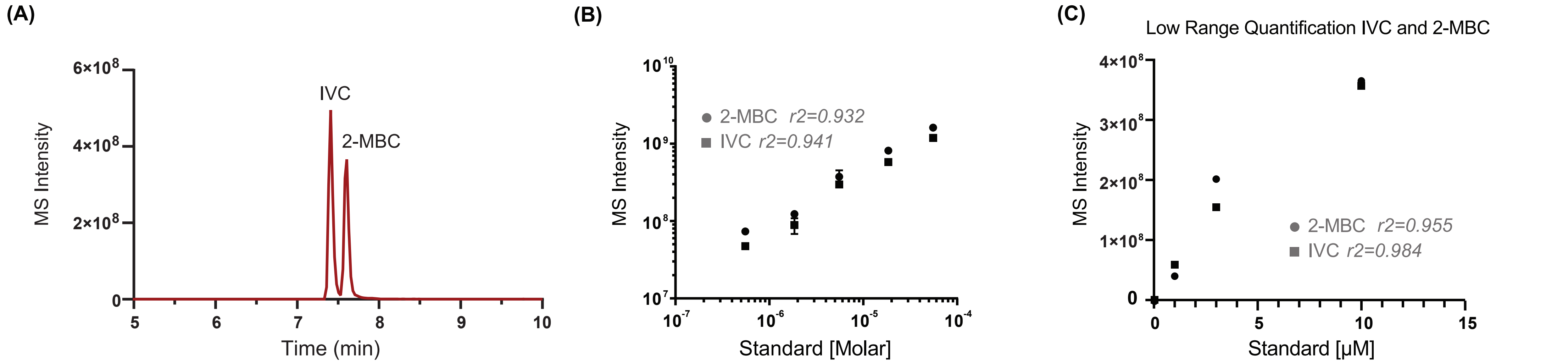
(A)
IVC
2-MBC
6×10^8
4×10^8
2×10^8
0
MS Intensity
5
6
7
8
9
10
Time (min)
(B)
10^10
10^9
10^8
10^7
MS Intensity
2-MBC r2=0.932
IVC r2=0.941
10^-7
10^-6
10^-5
10^-4
Standard [Molar]
(C)
Low Range Quantification IVC and 2-MBC
4×10^8
3×10^8
2×10^8
1×10^8
0
MS Intensity
2-MBC r2=0.955
IVC r2=0.984
0
5
10
15
Standard [µM]

Supplementary Figure 4. Targeted quantification of 2-MBC and IVC. (A) Representative parallel reaction monitoring chromatogram of a 10μM (each analyte) curve point using synthetic IVC and 2-MBC. **(B)** The predominant fragment for both 2-MBC and IVC was measured $[C_7H_{12}O_2]^+$ (theoretical 129.0910 m/z). X-axis, standard curve point concentration, in duplicate. Y-axis, maximum peak height was reported within the retention time window. Data shown as log-log plot, standard deviation may be smaller than symbol. **(C)** Low range curve quantification. For female conditioned media, samples had detectable levels of 2-MBC and IVC, but the best fit curve of all points led to skewed quantification (high relative deviation from the line). Therefore, we used a narrower range of curve points spanning below and above the female sample signals to improve quantification accuracy of these low-intensity signals. Source data are provided as a Source Data file.

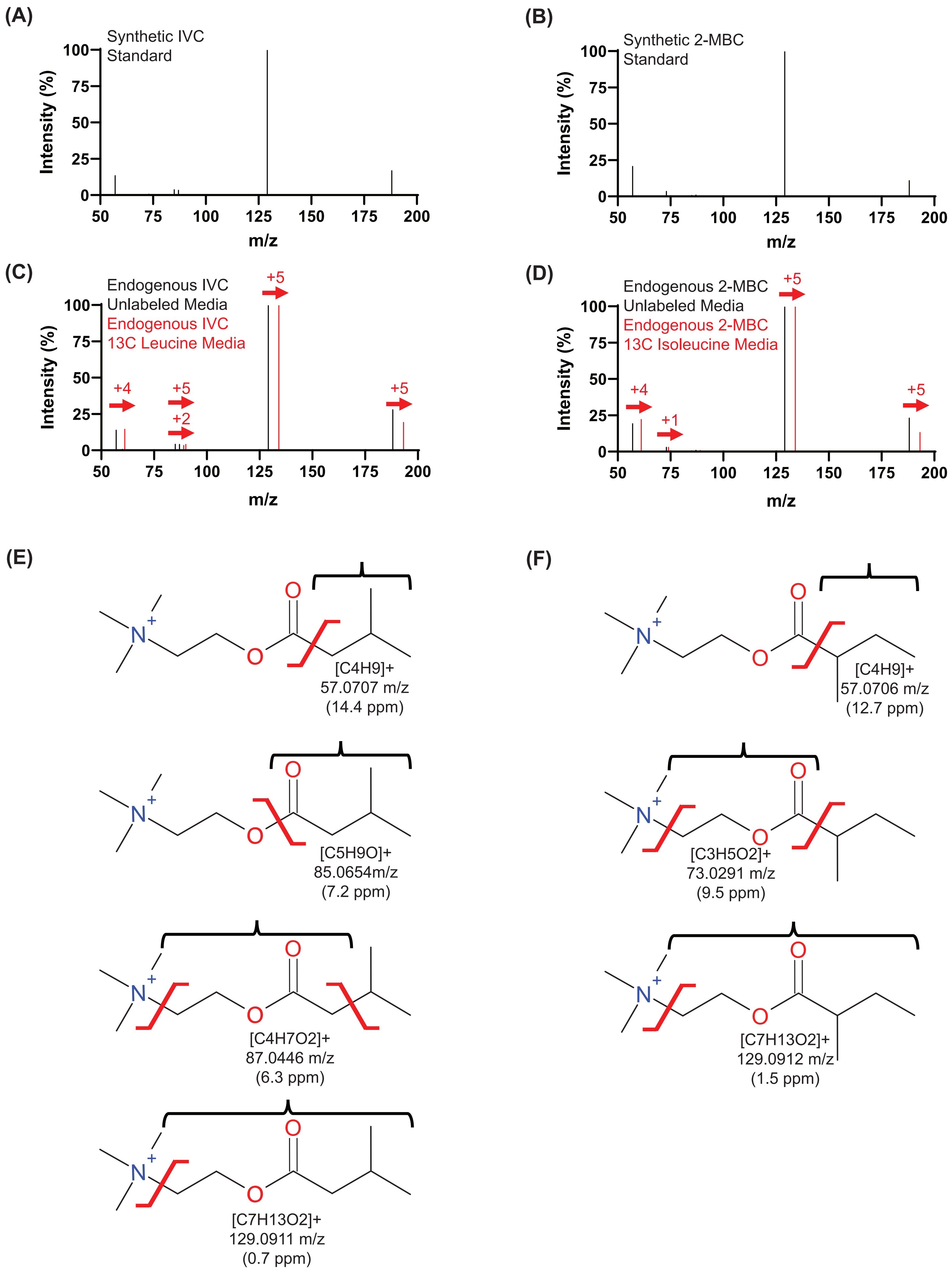

(A)
Synthetic IVC Standard
Intensity (%)
m/z
(B)
Synthetic 2-MBC Standard
Intensity (%)
m/z
(C)
Endogenous IVC Unlabeled Media
Endogenous IVC 13C Leucine Media
+4
+5
+2
+5
+5
Intensity (%)
m/z
(D)
Endogenous 2-MBC Unlabeled Media
Endogenous 2-MBC 13C Isoleucine Media
+4
+1
+5
+5
Intensity (%)
m/z
(E)
[C4H9]+ 57.0707 m/z (14.4 ppm)
[C5H9O]+ 85.0654m/z (7.2 ppm)
[C4H7O2]+ 87.0446 m/z (6.3 ppm)
[C7H13O2]+ 129.0911 m/z (0.7 ppm)
(F)
[C4H9]+ 57.0706 m/z (12.7 ppm)
[C3H5O2]+ 73.0291 m/z (9.5 ppm)
[C7H13O2]+ 129.0912 m/z (1.5 ppm)

Supplemental Figure 5: Tandem mass spectral analysis of isotopic fate. **(A)** MS2 of the synthetic IVC standard, and **(B)** MS2 of the synthetic 2-MBC standard. **(C)** Substructure and fate of 13C-U-Leucine into IVC (MS2) and **(D)** substructure and fate of 13C-U-Isoleucine into 2-MBC (MS2). Parent and characteristic fragment ions show the predicted mass shifts for both compounds. Isotopic label was also confirmed by apparent mass defect (1.00335 5ppm tolerance), confirming carbon-13 as the source of the mass shift. Relative fragment intensities are unaffected by incorporation of the isotopic labels. **(E-F)** Substructure assignments for each fragment ion observed above 1% relative intensity for IVC and 2-MBC, respectively. Red bars indicate location of bond break during MS/MS to generate the fragment. Black brackets indicate moiety of structure associated with each fragment m/z.

SUPPLEMENTARY METHODS

Synthetic schemes and NMR spectra related to synthesis of metabolites

Multiplicities in the following experimental procedures are abbreviated as follows: s = singlet, d = doublet, t = triplet, q = quartet, quint = quintet, sext = sextet, hept = heptet, br = broad, m = multiplet. Proton chemical shifts are expressed in parts per million (ppm, δ scale) and are referenced to the residual protium in the NMR solvent ($CDCl_3$: δ = 7.26). Carbon chemical shifts are expressed in ppm (δ scale) and are referenced to the carbon resonance of the NMR solvent ($CDCl_3$: δ = 77.16).

2-(dimethylamino)ethyl pentanoate

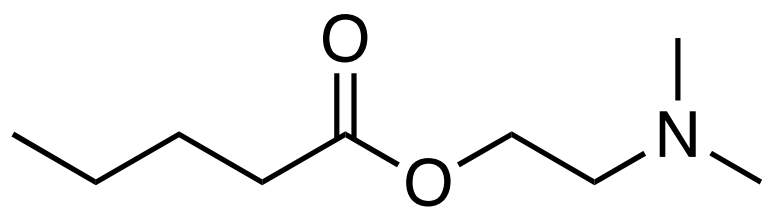

Valeric acid (541 μL, 4.90 mmol, 1.0 equiv.) was dissolved in a 1 M solution of $SOCl_2$ in CH_2Cl_2 (10.0 mL) and stirred at room temperature for 16 h. Volatiles were removed under reduced pressure and the residue was dissolved in dry CH_2Cl_2 (10.0 mL). 2-Dimethylaminoethanol (524 mg, 5.88 mmol, 1.2 equiv.) was added and stirred at room temperature for 16 h, washed with 1 M NaOH, dried, filtered, and the solvent was evaporated under reduced pressure. The crude mixture was purified by flash column chromatography (CH_2Cl_2 to 20% MeOH in CH_2Cl_2) to yield **2-(dimethylamino)ethyl pentanoate** as colorless oil (147 mg, 0.851 mmol, 17%).

^{1}H NMR (400 MHz, $CDCl_3$) δ 4.17 (t, J = 5.8 Hz, 2H), 2.56 (t, J = 5.8 Hz, 2H), 2.33 (t, J = 7.6 Hz, 2H), 2.29 (s, 6H), 1.61 (p, J = 7.6 Hz, 2H), 1.34 (h, J = 7.4 Hz, 2H), 0.91 (t, J = 7.3 Hz, 3H).

^{13}C NMR (100 MHz, $CDCl_3$) δ 174.1, 62.2, 58.0, 45.9, 34.1, 27.2, 22.4, 13.9.

HRMS: m/z calcd. for $C_9H_{20}NO_2^+$ ($[M+H]^+$): 174.1489 found: 174.1496.

2-(dimethylamino)ethyl 2-methylbutanoate

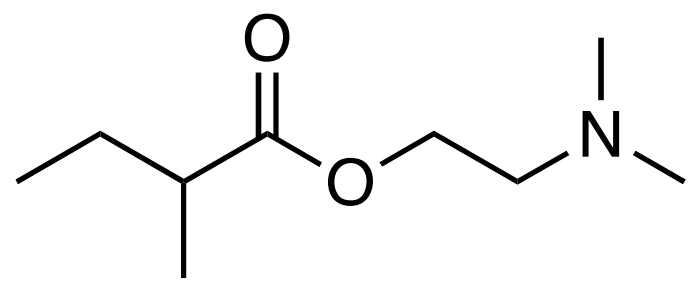

Racemic 2-methylbutanoic acid (541 μL, 4.90 mmol, 1.0 equiv.) was dissolved in a 1 M solution of $SOCl_2$ in CH_2Cl_2 (10.0 mL) and stirred at room temperature for 16 h. Volatiles were removed under reduced pressure and the residue was dissolved in dry CH_2Cl_2 (10.0 mL). 2-Dimethylaminoethanol (524 mg, 5.88 mmol, 1.2 equiv.) was added and stirred at room temperature for 16 h, washed with 1 M NaOH, dried, filtered, and the solvent was evaporated under reduced pressure. The crude mixture was purified by flash column chromatography (CH_2Cl_2 to 20% MeOH in CH_2Cl_2) to yield **2-(dimethylamino)ethyl 2-methylbutanoate** as colorless oil (62 mg, 0.358 mmol, 7%).

^{1}H NMR (400 MHz, $CDCl_3$) δ 4.19 (t, J = 5.5 Hz, 2H), 2.58 (s, 2H), 2.40 (h, J = 7.0 Hz, 1H), 2.30 (s, 6H), 1.68 (dp, J = 14.8, 7.4 Hz, 1H), 1.47 (dp, J = 14.6, 7.1 Hz, 1H), 1.14 (d, J = 7.0 Hz, 3H), 0.90 (t, J = 7.4 Hz, 3H).

^{13}C NMR (100 MHz, $CDCl_3$) δ 176.9, 62.2, 57.9, 45.8, 41.1, 26.9, 16.7, 11.7.

HRMS: m/z calcd. for $C_9H_{20}NO_2^+$ ($[M+H]^+$): 174.1489 found: 174.1484.

2-(dimethylamino)ethyl 3-methylbutanoate

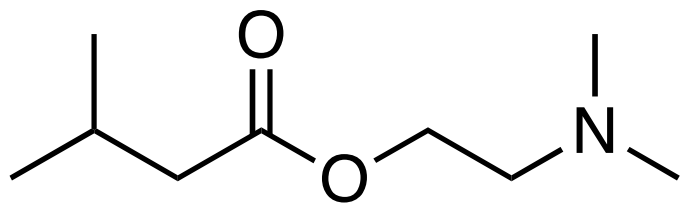

3-Methylbutanoic acid (541 µL, 4.90 mmol, 1.0 equiv.) was dissolved in a 1 M solution of $SOCl_2$ in CH_2Cl_2 (10.0 mL) and stirred at room temperature for 16 h. Volatiles were removed under reduced pressure and the residue was dissolved in dry CH_2Cl_2 (10.0 mL). 2-Dimethylaminoethanol (524 mg, 5.88 mmol, 1.2 equiv.) was added and stirred at room temperature for 16 h, washed with 1 M NaOH, dried, filtered, and the solvent was evaporated under reduced pressure. The crude mixture was purified by flash column chromatography (CH_2Cl_2 to 20% MeOH in CH_2Cl_2) to yield **2-(dimethylamino)ethyl 3-methylbutanoate** as colorless oil (100 mg, 0.577 mmol, 12%).

^{1}H NMR (400 MHz, $CDCl_3$) δ 4.17 (t, J = 5.8 Hz, 2H), 2.62 – 2.53 (m, 2H), 2.28 (s, 6H), 2.21 (d, J = 7.3 Hz, 2H), 2.10 (dp, J = 14.0, 6.9 Hz, 1H), 0.95 (d, J = 6.6 Hz, 6H).

^{13}C NMR (100 MHz, $CDCl_3$) δ 173.3, 62.1, 58.0, 45.8, 43.5, 25.8, 22.5.

HRMS: m/z calcd. for $C_9H_{20}NO_2^+$ ($[M+H]^+$): 174.1489 found: 174.1495.

N,N,N-trimethyl-2-(pentanoyloxy)ethan-1-aminium iodide

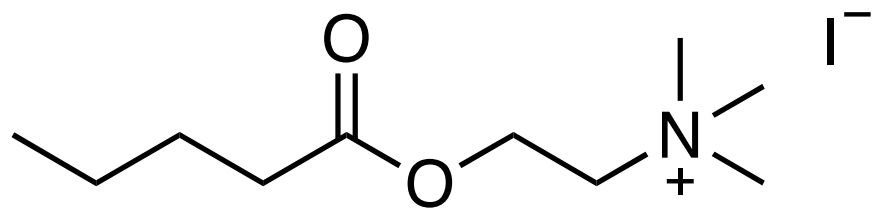

2-(dimethylamino)ethyl pentanoate (143 mg, 0.850 mmol, 1.0 equiv.) was dissolved in CH_2Cl_2 (10.0 mL). Methyl iodide (2.41 g, 17.0 mmol, 20.0 equiv.) was added and stirred at room temperature for 4 h. Heptanes were added and the formed precipitate was filtered off and washed with heptanes. **N,N,N-trimethyl-2-(pentanoyloxy)ethan-1-aminium iodide** was obtained as colorless solid (209 mg, 0.664 mmol, 78%).

^{1}H NMR (400 MHz, $CDCl_3$) δ 4.58 (s, 2H), 4.17 – 4.12 (m, 2H), 3.56 (s, 9H), 2.39 (t, J = 7.6 Hz, 2H), 1.63 (q, J = 7.5 Hz, 2H), 1.34 (m, 2H), 0.92 (t, J = 7.3 Hz, 3H).

^{13}C NMR (100 MHz, $CDCl_3$) δ 172.8, 65.5, 57.6, 55.0, 33.9, 26.8, 22.4, 13.8.

HRMS: m/z calcd. for $C_{10}H_{22}NO_2^+$ ($[M-I]^+$): 188.1645 found: 188.1651.

N,N,N-trimethyl-2-((2-methylbutanoyl)oxy)ethan-1-aminium iodide

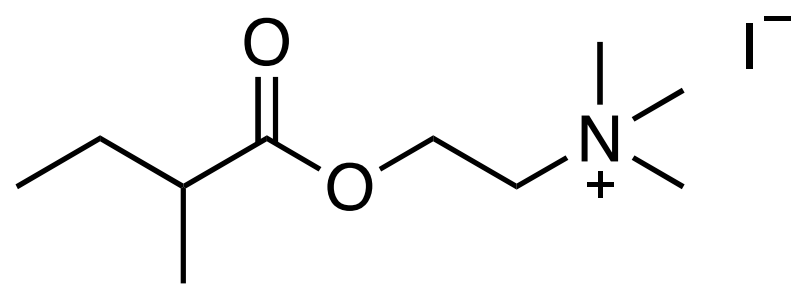

2-(dimethylamino)ethyl 2-methylbutanoate (62.0 mg, 0.358 mmol, 1.0 equiv.) was dissolved in CH_2Cl_2 (4.2 mL). Methyl iodide (1.02 g, 7.16 mmol, 20.0 equiv.) was added and stirred at room temperature for 4 h. Heptanes were added and the formed precipitate was filtered off and washed with heptanes. **N,N,N-trimethyl-2-((2-methylbutanoyl)oxy)ethan-1-aminium iodide** was obtained as colorless solid (67.0 mg, 0.213 mmol, 59%).

^{1}H NMR (400 MHz, $CDCl_3$) δ 4.59 (s, 2H), 4.13 – 4.08 (m, 2H), 3.53 (s, 9H), 2.45 (p, J = 6.9 Hz, 1H), 1.69 (dq, J = 14.7, 7.3 Hz, 1H), 1.51 (dq, J = 13.9, 7.2 Hz, 1H), 1.18 (d, J = 7.0 Hz, 3H), 0.92 (t, J = 7.4 Hz, 3H).

^{13}C NMR (100 MHz, $CDCl_3$) δ 175.7, 65.6, 57.7, 55.0, 40.9, 26.7, 16.5, 11.8.

HRMS: m/z calcd. for $C_{10}H_{22}NO_2^+$ ($[M-I]^+$): 188.1645 found: 188.1643.

N,N,N-trimethyl-2-((3-methylbutanoyl)oxy)ethan-1-aminium iodide

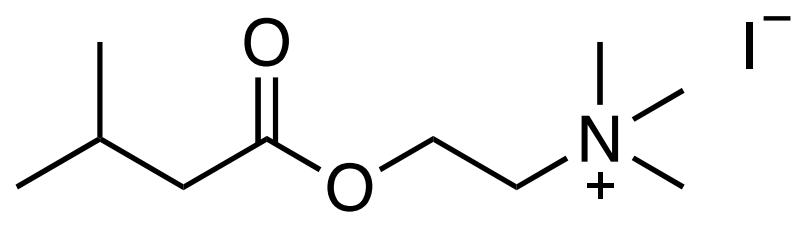

2-(dimethylamino)ethyl 3-methylbutanoate (100 mg, 0.577 mmol, 1.0 equiv.) was dissolved in CH_2Cl_2 (6.8 mL). Methyl iodide (1.64 g, 11.5 mmol, 20.0 equiv.) was added and stirred at room temperature for 4 h. Heptanes were added and the formed precipitate was filtered off and washed with heptanes. **N,N,N-trimethyl-2-((3-methylbutanoyl)oxy)ethan-1-aminium iodide** was obtained as colorless solid (134 mg, 0.426 mmol, 74%).

^{1}H NMR (400 MHz, $CDCl_3$) δ 4.58 (s, 2H), 4.16 – 4.10 (m, 2H), 3.55 (s, 9H), 2.27 (d, J = 7.1 Hz, 2H), 2.16 – 2.06 (m, 1H), 0.97 (d, J = 6.6 Hz, 6H).

^{13}C NMR (100 MHz, $CDCl_3$) δ 172.1, 65.5, 57.6, 55.0, 43.1, 25.7, 22.6.

HRMS: m/z calcd. for $C_{10}H_{22}NO_2^+$ ($[M-I]^+$): 188.1645 found: 188.1642.

^{1}H, and ^{13}C NMR Spectra

2-(dimethylamino)ethyl pentanoate

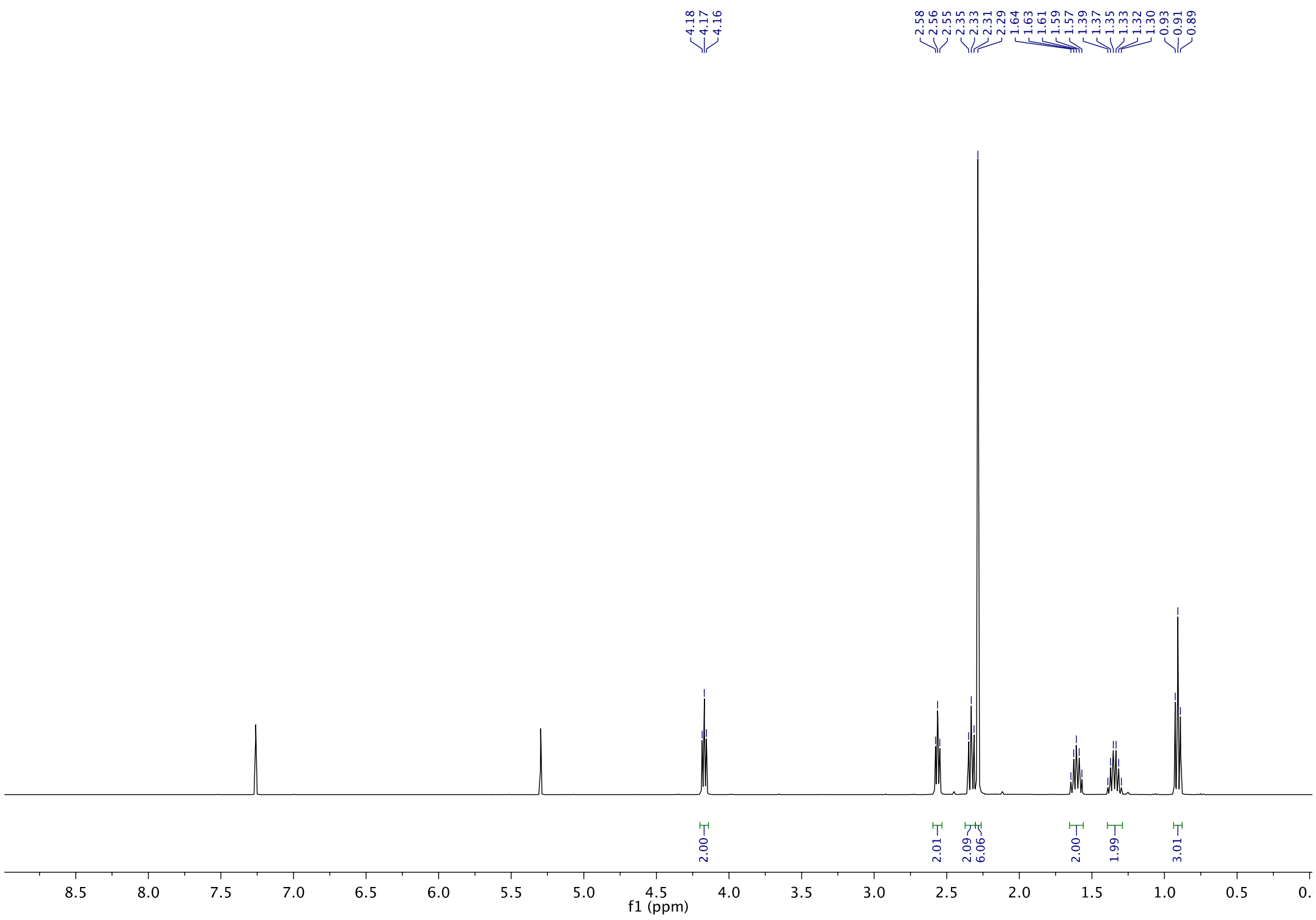

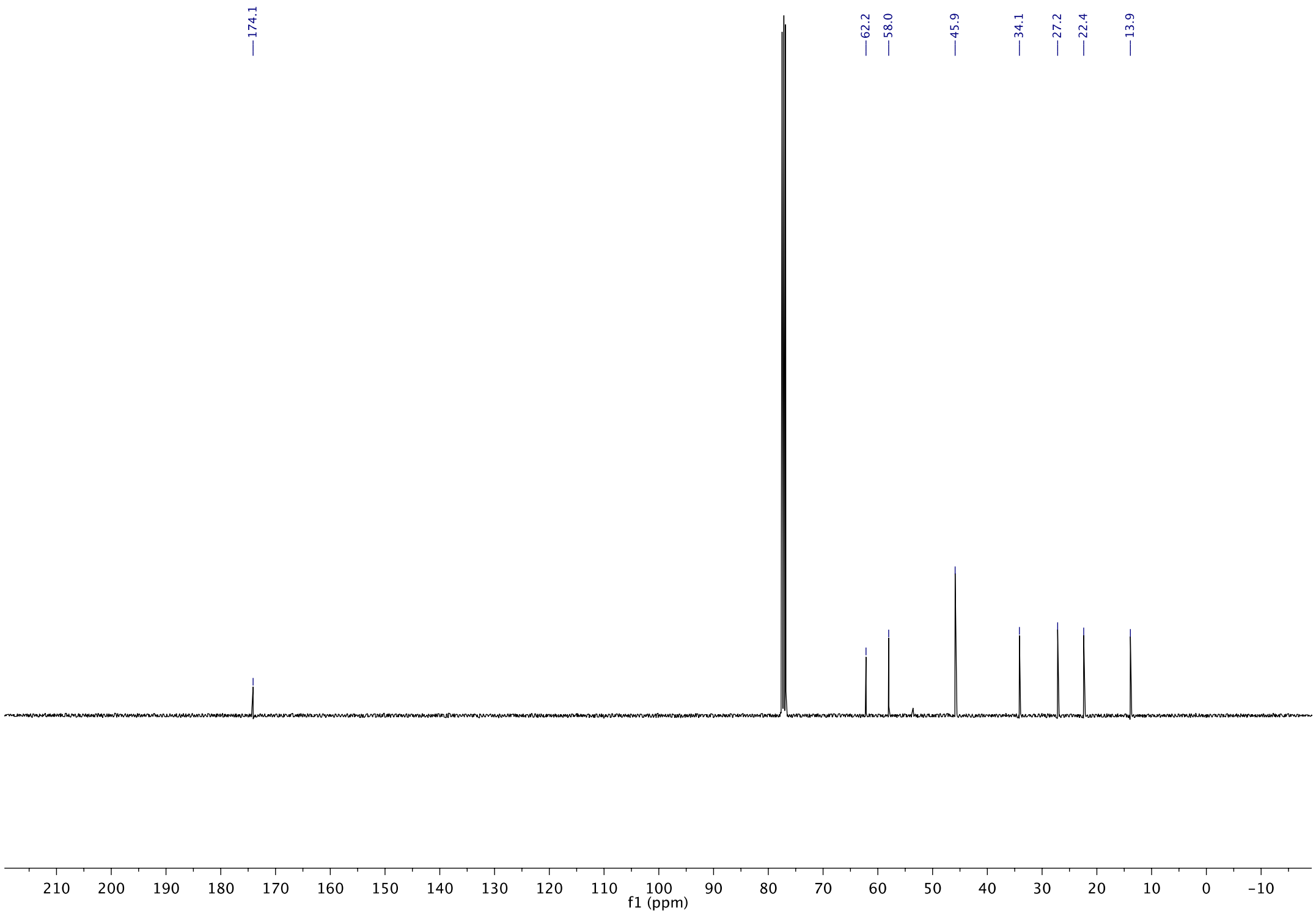

2-(dimethylamino)ethyl 2-methylbutanoate

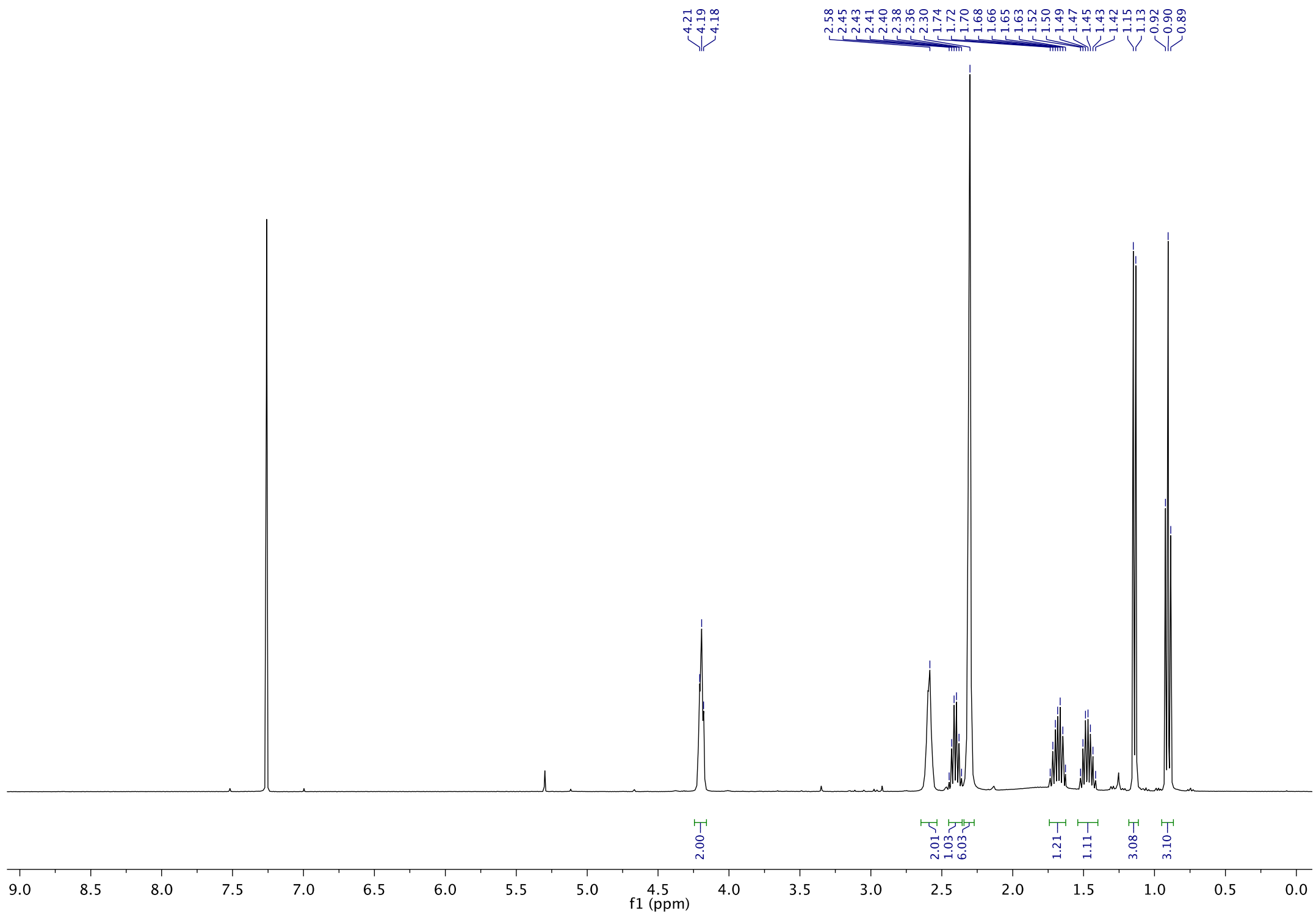

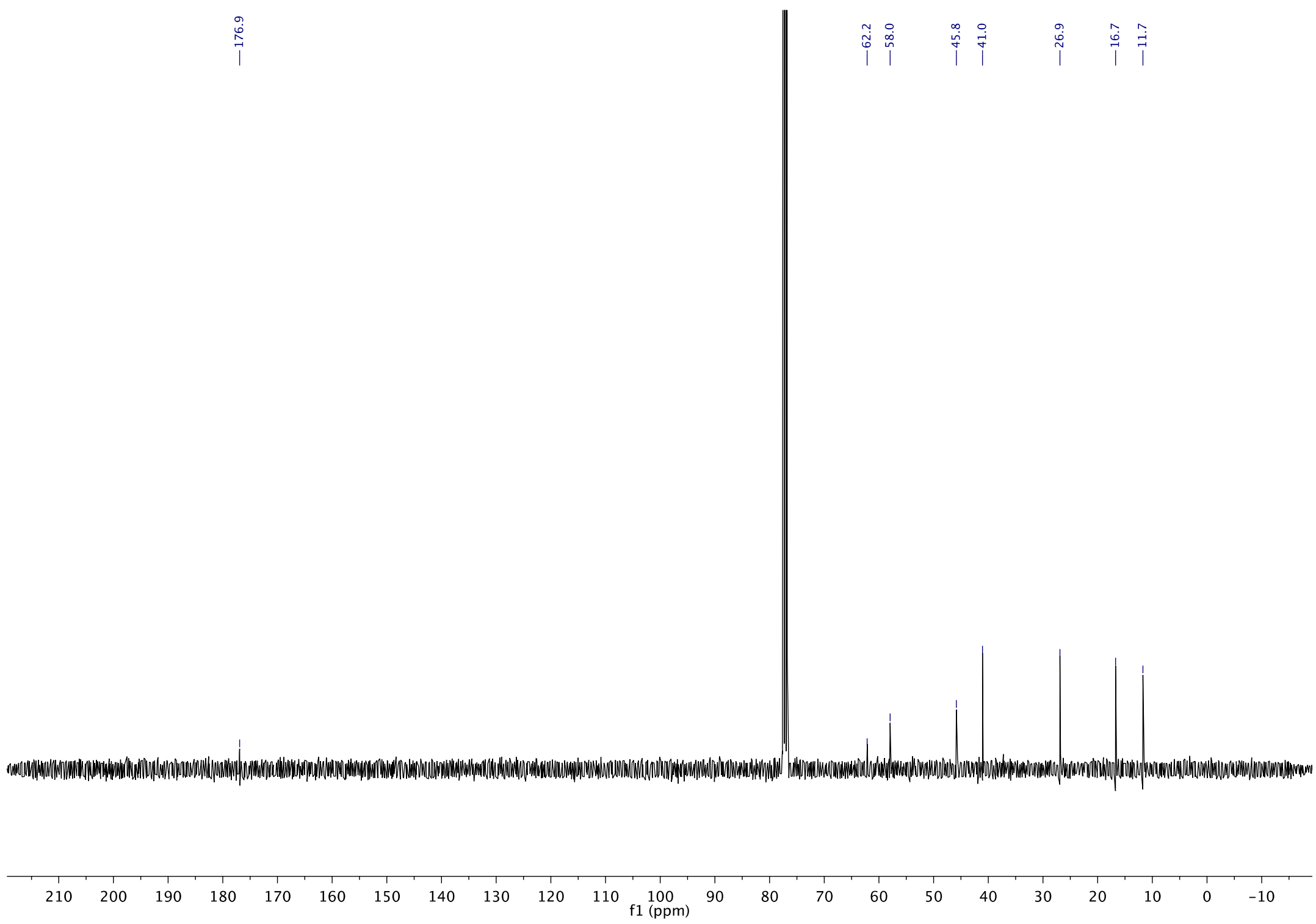

2-(dimethylamino)ethyl 3-methylbutanoate

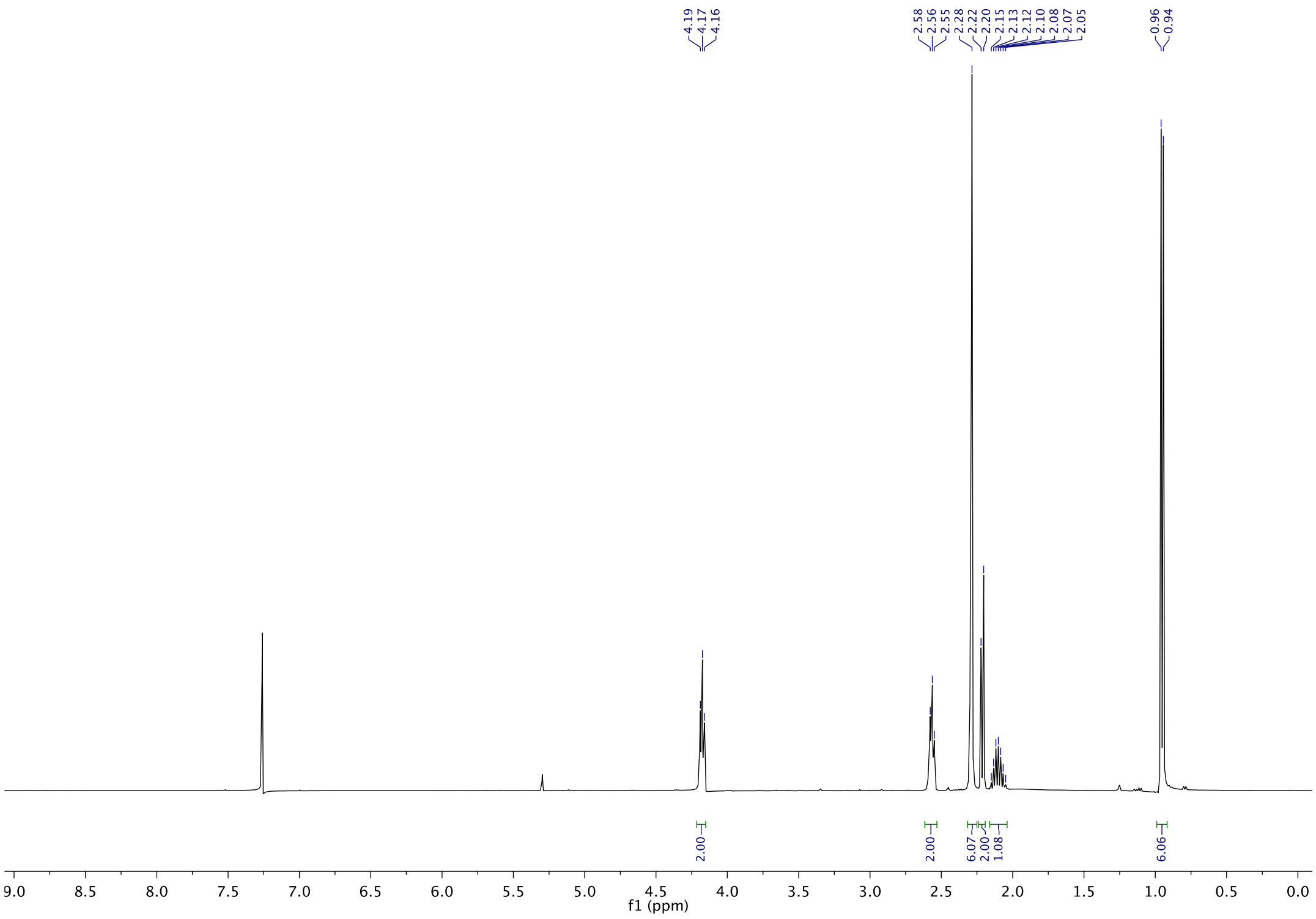

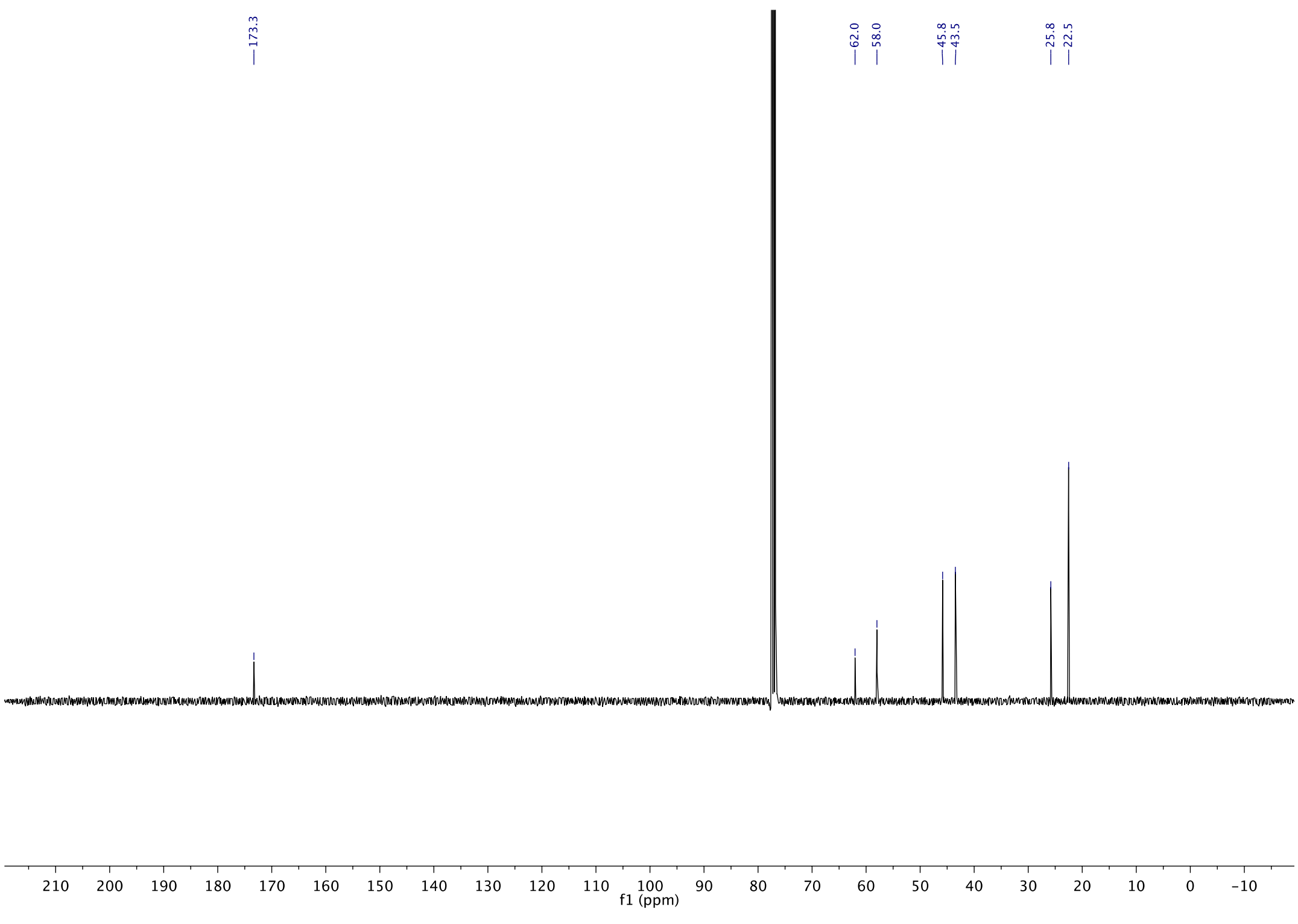

N,N,N-trimethyl-2-(pentanoyloxy)ethan-1-aminium iodide

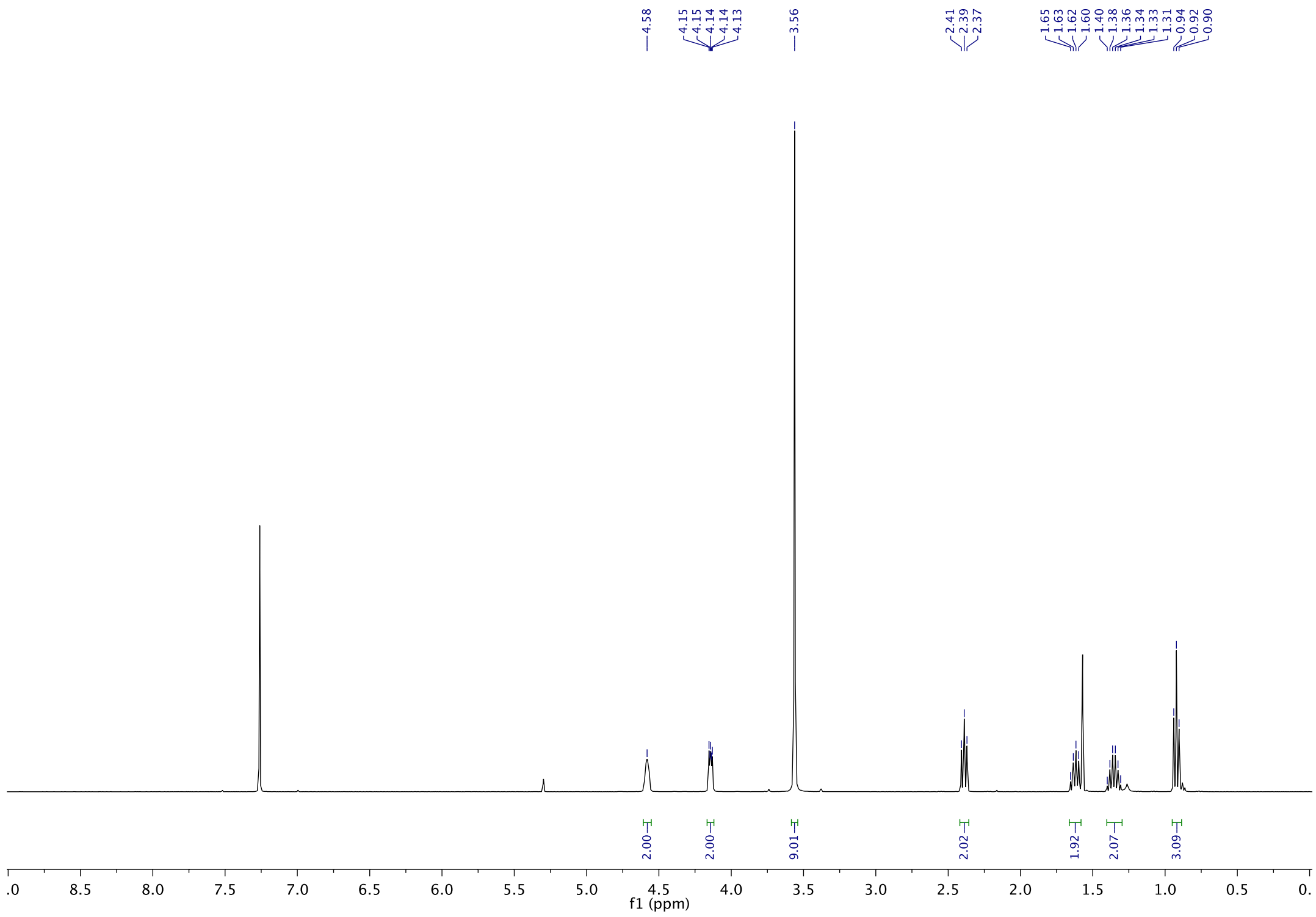

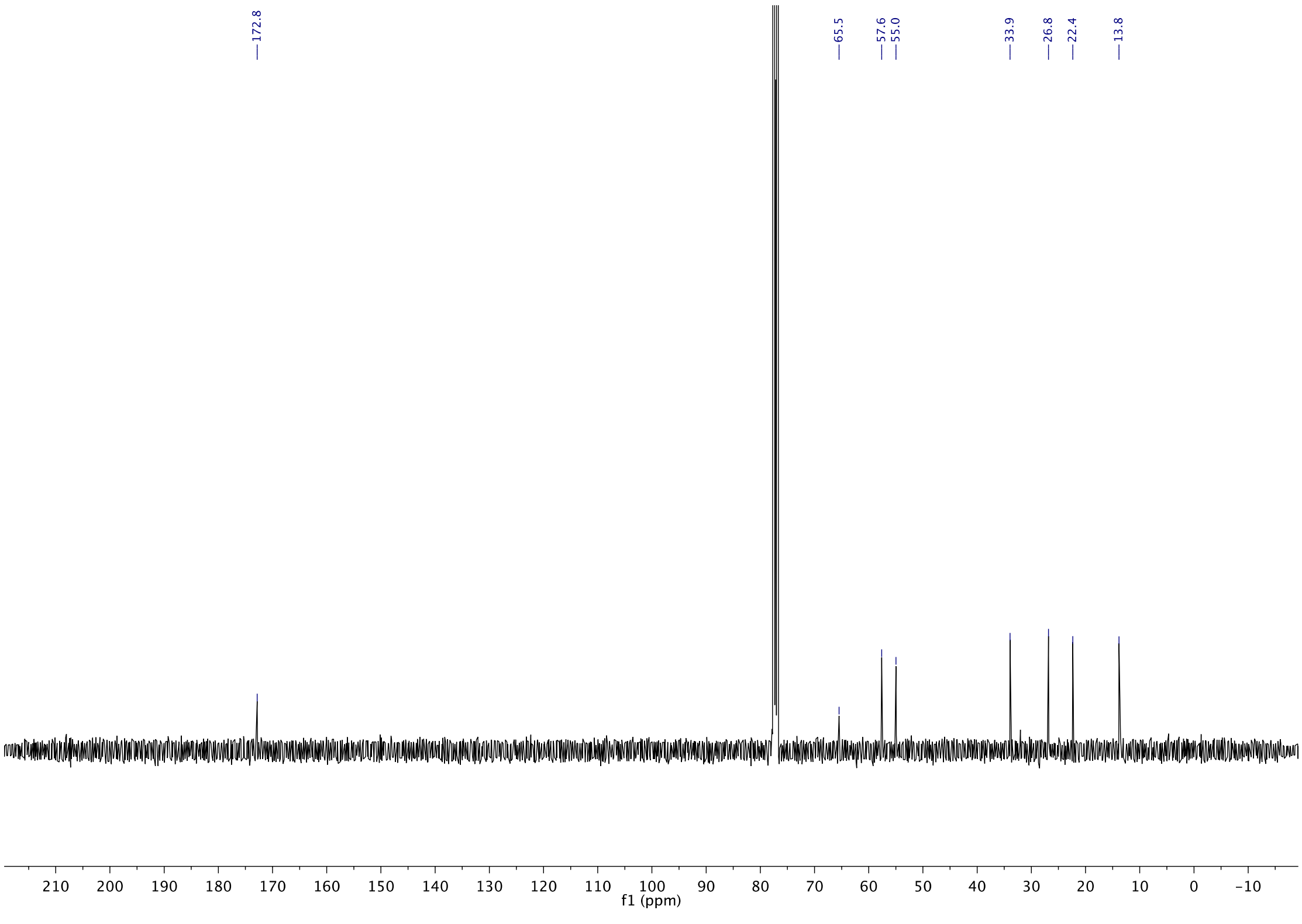

N,N,N-trimethyl-2-((2-methylbutanoyl)oxy)ethan-1-aminium iodide

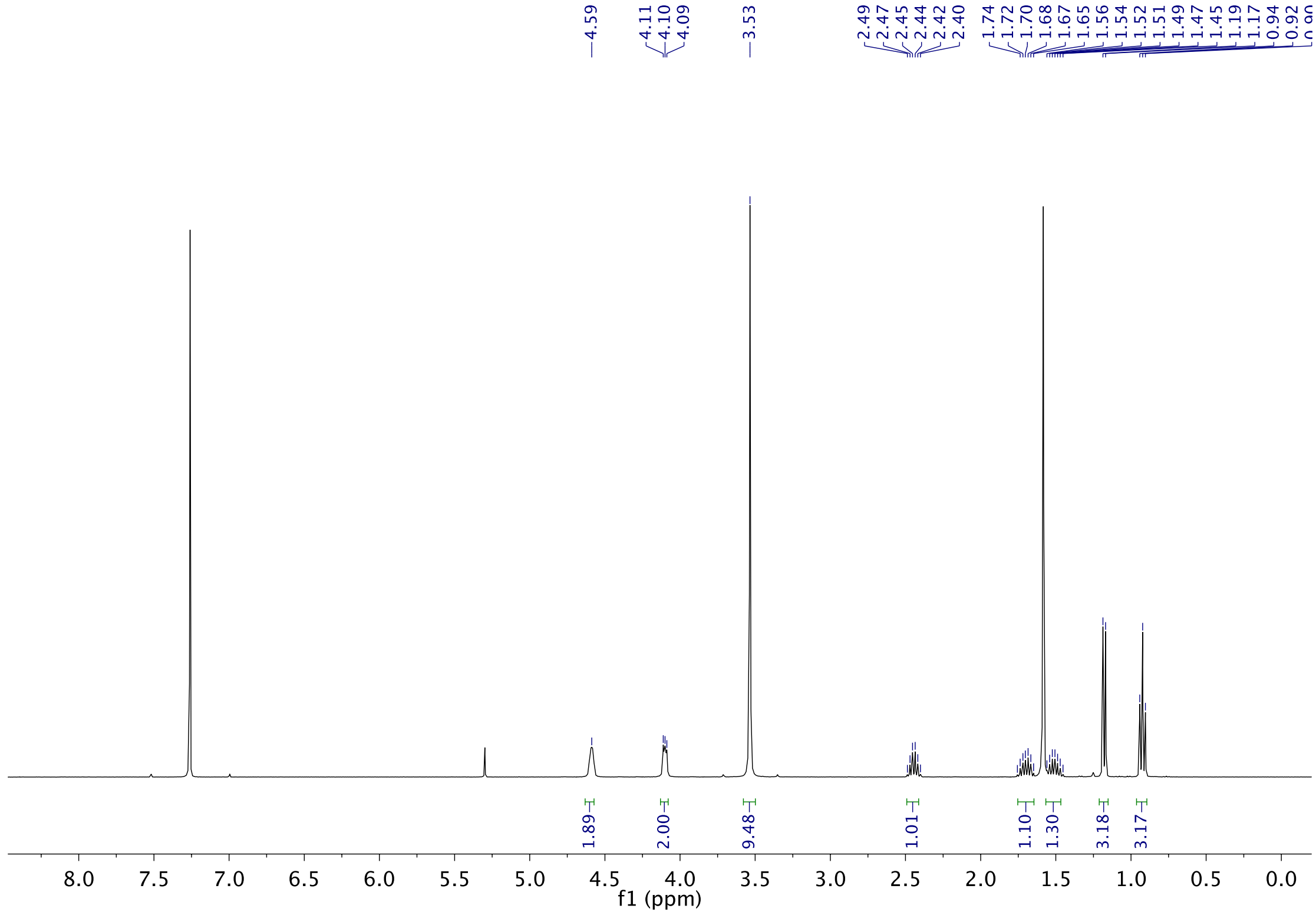

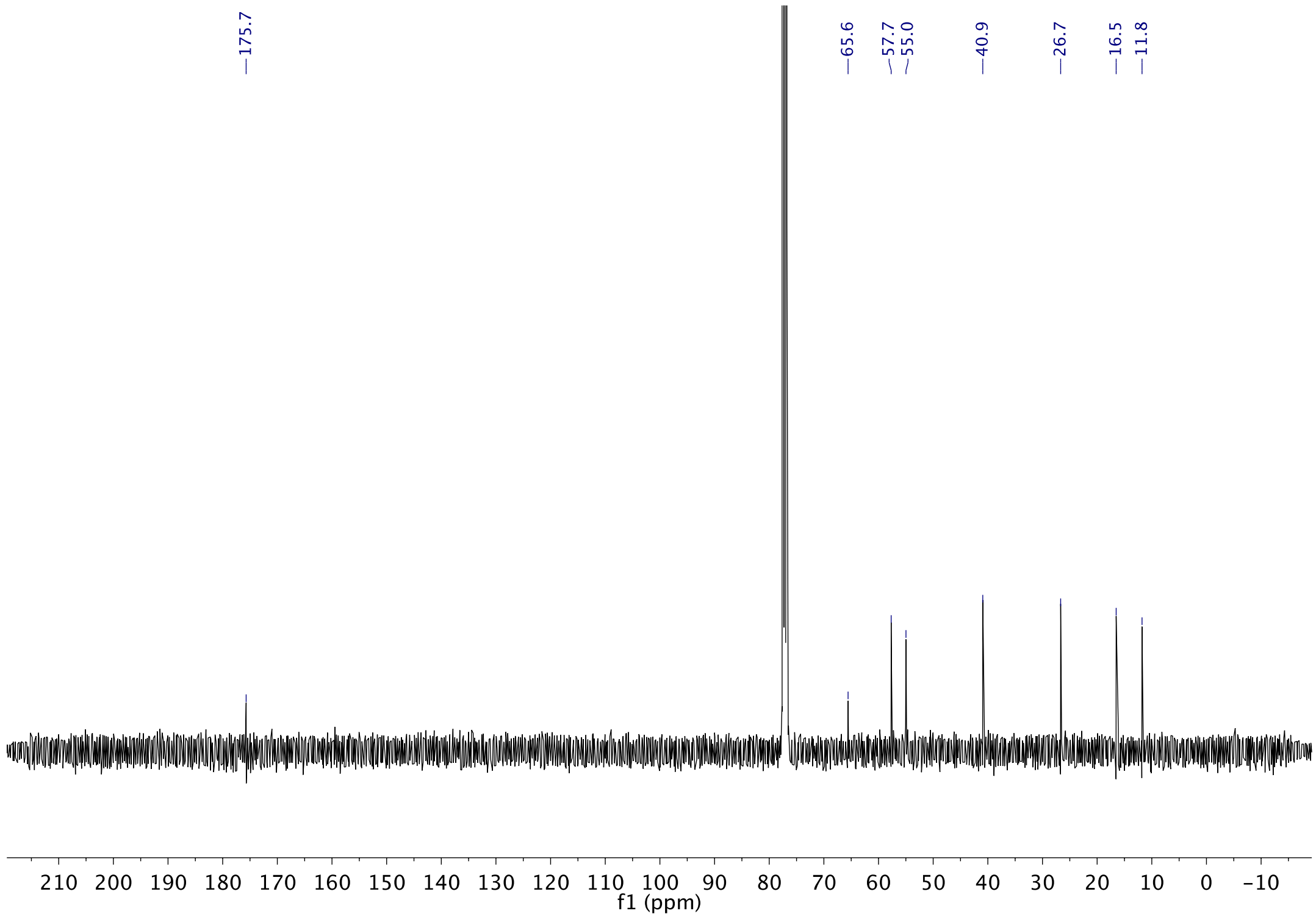

N,N,N-trimethyl-2-((3-methylbutanoyl)oxy)ethan-1-aminium iodide

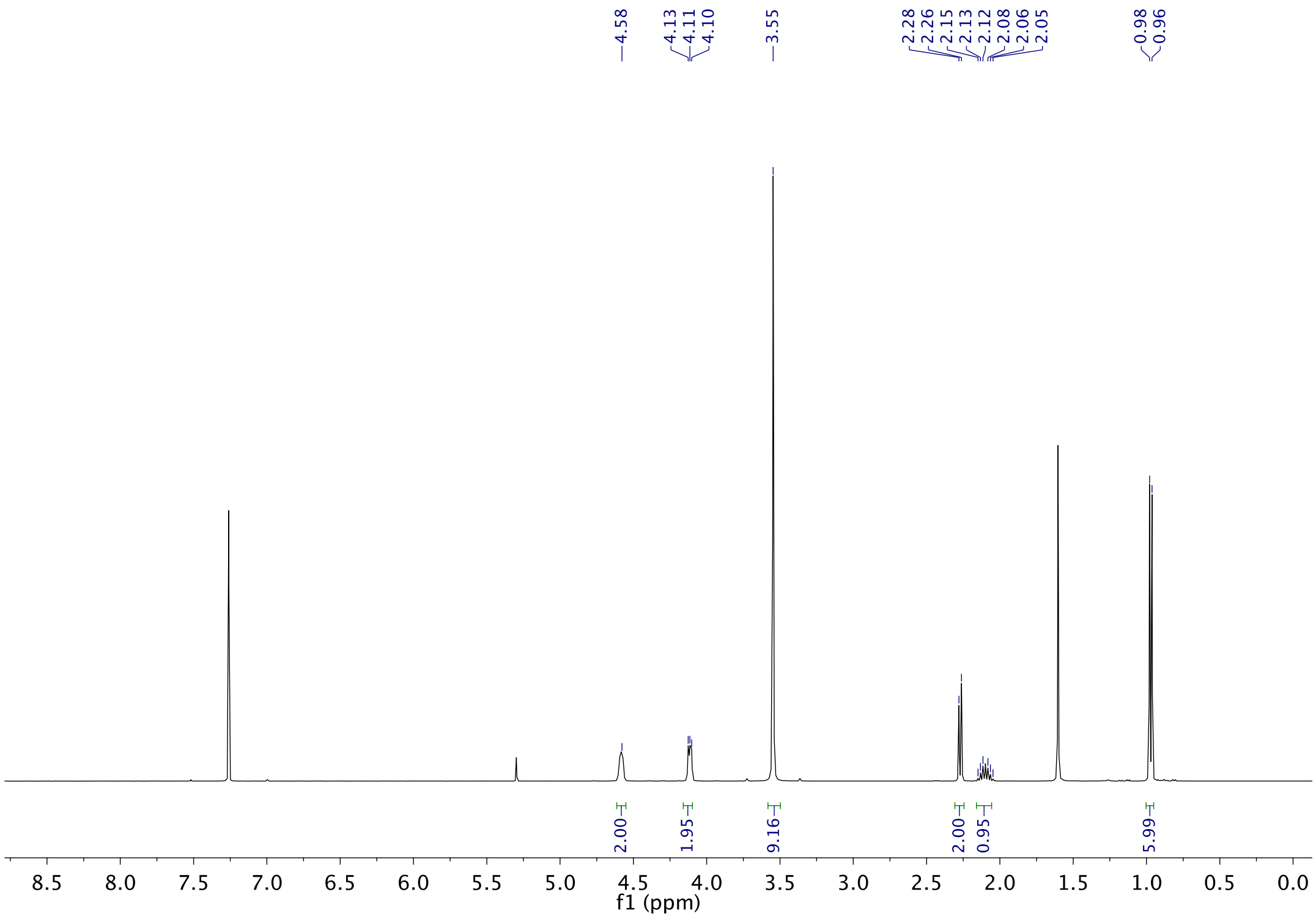

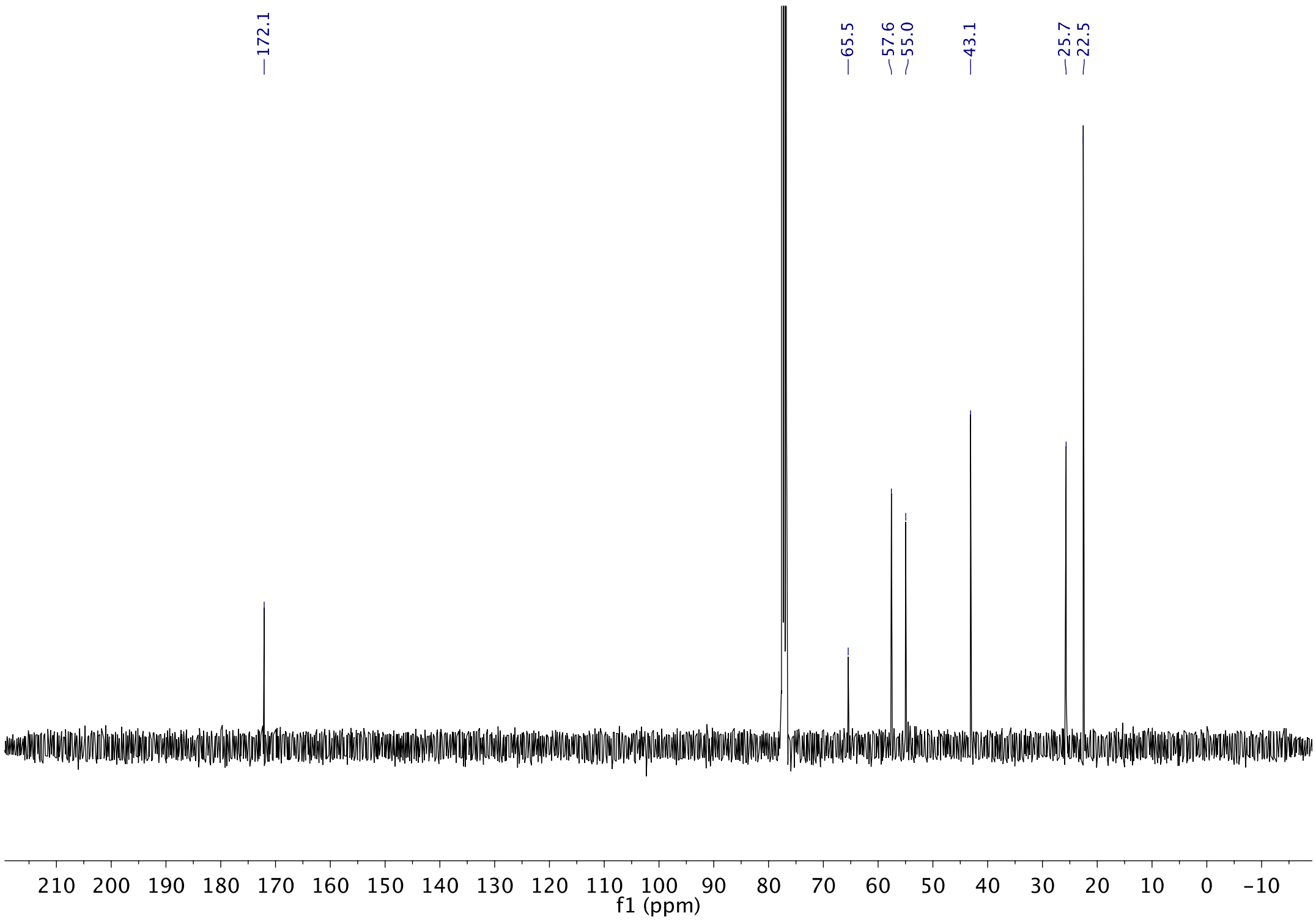

Supplementary Table 1: Details on experimental design for each section of the Results
dpi: days post-infection

Results Section	Data collected	Worm stages	Sample type	# worms (species)	Biological replicates	Analyses	Comparisons	Stat test	Figures	Tables
**Metabolic profiling of filarial worms identifies a unique sex-specific metabolite**	Global/ Untargeted MS	infective L3, MF (L1), adult males (120 dpi), adult females (120 dpi)	Metabolites from whole worms	20 adult female worms, 40 adult males, 2X10^6 microfilaria, and 200 L3 larvae per biological replicate (*Brugia malayi*)	n=3	MS profiles; spectral library searches (NIST17)	cross stages	N/A	Fig 1A Fig 2A	Supp. Data 1 Supp. Data 2
		adult males, adult females	Metabolites from spent media (ES) and unspent media (control)	2 worms per 2 ml (*B. malayi*)	n=3	Spectral library search; Unsupervised clustering (Euclidian distance function); PCA	male vs female vs control media (unspent media)	Two-tailed t-test, z-score transformation, and principle component analysis	Supp. Fig 1A Supp. Fig 1B	Supp. Data 3
		adult males, adult females	Metabolites from spent media (ES)	2 worms per 2 ml (*B. malayi*)	n=3	Hierarchical clustering of significant differences	male vs female spent media	Two-tailed t-test and z-score transformation	Supp Fig 1C	
		adult males, adult females	Metabolites from spent media (ES)	2 worms per 2 ml (*B. malayi, Brugia pahangi, Acanthocheilonema viteae*)	n=3 for *B. malayi* and *A. viteae*; n=6 for *B. pahangi*	Volcano plots; Relative quantification of MS peaks	male vs female spent media	Two-tailed t-test (raw p-values)	Fig 1B	Supp. Data 4 Supp. Data 5 Supp. Data 6
**The filarial metabolites are acetylcholine analogs**	Global/ Untargeted MS	adult males, adult females	Metabolites from spent media (ES)	*B. pahangi*	n=6	Molecular networking	male vs female spent media	N/A	Supp Fig 2	
	Targeted MS	adult males	Metabolites from spent media (ES) with spike-in of synthesized metabolites	*B. pahangi*	N/A	Evaluation of chromatographic co-elution, MS1, MS2 spectra match	Endogenous peaks vs chemical standards	N/A	Supp Fig 3	
**Acetylcholine analogs are produced by male and female *B. malayi* but preferentially secreted by males**	Targeted MS	adult males, adult females	Metabolites from whole worms	10 worms per sample (*B. malayi*, *A. viteae*)	n=3	Quantification of analytes	male vs female	Multiple unpaired t-tests (FDR at 1%)	Fig 2B	
	Targeted MS	adult males, adult females	Metabolites from spent media (ES)	1-4 worms per sample (*B. malayi*)	n=3	Quantification of analytes and MS peaks	male vs female spen	N/A (females do not secrete the metabolites so comparison would be zero)	Fig 2C Supp Fig 4C	
**Biosynthetic pathways of filarial acetylcholine analogs use leucine and isoleucine as substrates**	Targeted MS	adult males	Metabolites from spent media (ES) and unspent media (control)	3 worms per sample (*B. malayi*)	n=2	Isotope tracing and fate	Plus and minus isotopic precursors	N/A	Fig. 3 Supp Fig 5	
**Doxycycline treatment increases, and ethacrynic acid treatment decreases, filarial acetylcholine analog production by male *B. malayi* worms**	Global/ Untargeted MS	adult males	Metabolites from whole worms, treated with doxycycline (12.5 µM)	10 worms per sample (*B. malayi*)	n=3	Quantification of MS peaks	Untreated males vs treated males with doxycycline	Multiple unpaired t-tests (FDR at 1%)	Fig 4A	
	Global/ Untargeted MS	adult males	Metabolites in ES of male worms treated with Ethacrynic Acid (5 µM and 50 µM)	3 worms per sample (*B. malayi*)	n=3	Quantification of analytes	Untreated and treated with different concentratons of ethacrynic acid	Multiple unpaired t-tests (FDR at 1%)	Fig 4B	
**Filarial acetylcholine analogs impact genes involved in worm reproduction**	RNAseq	adult males, adult females	RNA from whole worms untreated or treated with IVC and 2-MBC combined (10 µM)	5 worms per sample (*B. malayi*)	n=2	Differentially expressed genes and enrichment analysis	Untreated vs treated worms with combined metabolites	One-tailed hypergeometric test	Fig 5A	Supp. Data 7 Supp. Data 8
	egg laying	one-day old *C. elegans* Bristol strain N2	Whole worms treated with 1% DMSO (control), IVC (10 µM) or 2-MBC (10 µM)	5 worms per group (*C. elegans*)	n=5	Released eggs per 2 hours counted with a dissection stereomicroscope	*C. elegans* worms treated with IVC or 2-MBC compared to DMSO control	One-way ANOVA, followed by Holm-Šidák's multiple comparisons test	Fig 5B	
	MF release	adult females	Whole worms treated with 0.1% DMSO (control), IVC and 2-MBC combined (10 µM)	2 worms per sample (*B. malayi*)	Control: d1 n=6; d2 n=5; d3 n=4 Treated: d1 and d2 n=7; d3 n=6	Number of released microfilariae per 24 hours	*B. malayi* worms treated with IVC and 2-MBC compared to DMSO control over 3 days	Unpaired t-test	Fig 5C	

Results Section	Data generated	Assay	Sample Type	Species (#animals when applicable)	Biological or technical replicates	Analysis	Comparisons	Stat test	Figures	Data Tables
**Filarial acetylcholine analogs activate mammalian muscarinic acetylcholine receptor M2**	Human GPCR expression	PRESTO-Tango platform, a luciferase reporter-based system that converts activation into luminescence signal	CHRM1-5 Plasmid-transfected HEK293 cells treated with DMSO (control), IVC, 2-MBC, or a combination of both IVC+2-MBC (20 µM)	Human genes	n=3	Differences in fold change luminescence as measured with a Biotek synergy h1 instrument	Differences in fold change between muscarinic receptors and between treatments	One-way ANOVA, followed by Tukey's multiple comparisons test	Fig 6A	
	Molecular docking binding poses and energies	Modeling of molecular interactions	in silico, protein strctures, chemical structure of analogs	*B. malayi* M2 (ID: A0A8L7YLR4), human M2 (PDB ID: 5YC8)	n=10 (docking poses)	Docking and binding energies, molecular interactions	Comparison between human and *B. malayi* M2 when binding is modeled with human Ach, and *B. malayi* IVC, and 2-MBC	N/A	Fig 6B Fig 6C	Supp. Data 9
**Filarial acetylcholine analogs impact the host lymphatics**	RNAseq		RNA from human lymphatic endothelial cells (LEC) treated with IVC and 2-MBC combined (20 µM)	Human LECs	n=3	Differentially expressed genes and KEGG nrichment analysis	Untreated cells vs treated cells with combined metabolites	One-tailed hypergeometric test	Fig 7A Fig 7B	Supp. Data 10
	Size of mouse lymphatic vessels	FITC dextran injected on day 5 day of treatment with or without metabolites (100 µM)	Dissected lymphatic vessels leading from the footpad to the popliteal lymph node of juvenile mice	Female C57BL/6 (8 mice per group)	n=8	Confocal microscope images	Mice treated with combined metabolites compared to mice treated with vehicle	N/A	Fig 7C	
	Mouse lymph flow	Inoculation with 10^4 focus-forming units of ZIKV subcutaneously in the same hind footpad on day 5 of treatment with or without metabolites	RNA from resected popliteal lymph nodes (pLNs)	Female C57BL/6 (5 mice in control group; 4 mice in treatment group)	n=17 for pLNs from control group; n=16 for treatment group	Copy number of the ZIKV capsid gene determined by qPCR	Mice treated with combined metabolites compared to mice treated with vehicle	Two-tailed Mann-Whitney U test	Fig 7D	
